# Loss of Heterozygosity in Pediatric Acute Lymphoblastic Leukemia and Its Prognostic Impact: A Retrospective Study

**DOI:** 10.3390/cancers17152500

**Published:** 2025-07-29

**Authors:** Borys Styka, Gabriela Ręka, Aleksandra Ozygała, Mariola Janiszewska, Magdalena Stelmach, Paulina Skowera, Zuzanna Urbańska, Monika Lejman

**Affiliations:** 1 Independent Laboratory of Genetic Diagnostics, Medical University of Lublin, 20-093 Lublin, Poland; gabrysia.reka@gmail.com (G.R.); aleksandra.ozygala@uszd.lublin.pl (A.O.); magdalena.stelmach@umlub.pl (M.S.); paulina.skowera@umlub.pl (P.S.); monika.lejman@umlub.pl (M.L.); 2 Department of Medical Informatics and Statistics with e-Health Lab, Medical University of Lublin, 20-059 Lublin, Poland; mariola.janiszewska@umlub.pl; 3 Department of Genetic Predisposition to Cancer, Medical University of Lodz, 92-216 Lodz, Poland; zuzanna.urbanska@umed.lodz.pl

**Keywords:** pediatric oncology, SNP arrays, molecular karyotype, *CDKN2A*, *CDKN2B*, hyperdiploidy, minimal residual disease

## Abstract

Background: In childhood acute lymphoblastic leukemia (ALL), in addition to classical chromosomal abnormalities, loss of heterozygosity (LOH), including copy-neutral LOH, is also observed. While LOH has been described in the literature, its clinical relevance in pediatric ALL remains unclear. The aim of this study is to identify and analyze patterns of LOH, assess their frequency, and evaluate their association with clinical characteristics and early treatment response during the induction phase of the ALL protocol. Methods: The study included 853 pediatric ALL patients, of whom 120 had B-ALL LOH+ and 58 had T-ALL LOH+. LOH was analyzed using CytoScan HD SNP microarrays. Patients were stratified using multiple correspondence analysis (MCA) and hierarchical clustering on principal components (HCPC), which identified three genetically and clinically distinct clusters. Results: In B-ALL, two clusters with extensive LOH—particularly involving chromosome 9—were associated with poor prognosis and suboptimal response to therapy. In contrast, Cluster 2, characterized by CDKN2A duplication and rare LOH, showed a favorable clinical course. In T-ALL, Cluster 1 had LOH in CDKN2A but favorable outcomes; Cluster 2 exhibited biallelic CDKN2A deletion and aggressive disease; Cluster 3 lacked CDKN2A alterations and showed a genetically stable profile. LOH was common on chromosomes not typically affected by trisomy and rare on those gained. Conclusions: Our study indicates that LOH profiling can positively influence patient stratification by identifying high-risk subgroups, inform prognosis by highlighting unfavorable genetic alterations, and help predict poor treatment response in specific clinical profiles.

## Simple Summary

In acute lymphoblastic leukemia (ALL), loss of heterozygosity (LOH), including copy-neutral LOH, occurs alongside classic chromosomal aberrations. The aim of this study is to identify and analyze patterns of LOH, assess their frequency, and evaluate their association with clinical characteristics and early treatment response during the induction phase of the ALL protocol. The study included 853 children with ALL: 120 patients with B-ALL LOH+ and 58 patients with T-ALL LOH+. LOH was analyzed using CytoScan HD SNP microarrays. Multiple correspondence analysis and hierarchical clustering identified three clusters based on genetic and clinical profiles. In B-ALL, two clusters showed poor prognosis and treatment response, while Cluster 2—with *CDKN2A* duplication and rare LOH—had favorable outcomes. In T-ALL, Cluster 1 had a good prognosis despite *CDKN2A* LOH; Cluster 2 showed aggressive features with biallelic deletions; Cluster 3 lacked LOH of the *CDKN2A* gene and was characterized by a genetically stable profile. In B-ALL, whole-chromosome LOH, especially of chromosome 9, was frequent, unlike in T-ALL. LOH was less common in chromosomes affected by trisomy.

## 1. Introduction

Acute lymphoblastic leukemia (ALL) is the most common pediatric neoplasm that originates from B- and T-lineage lymphoid precursors. ALL is initiated by a spectrum of genetic mutations that encompass aneuploidy and chromosomal rearrangements, and primarily affect genes involved in the development of lymphoid cells and the intricate regulation of their cell cycle. Mutations and rearrangements impact critical cellular transduction pathways and disturb genes that orchestrate the orderly progression of hematopoiesis. Moreover, they affect tumor suppressor genes, regulators of apoptosis, and oncogenes with the potential to spur uncontrolled cell growth. Consequently, the progression of ALL is marked by the relentless proliferation of abnormal and immature lymphocytes and their precursors [1,2,3].

**Figure 1:**
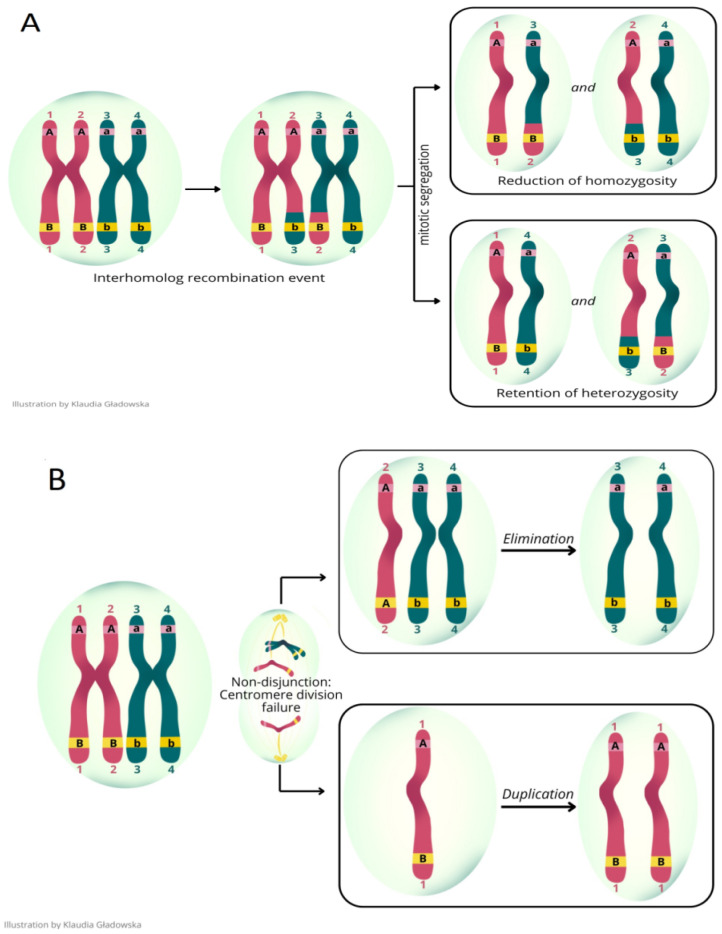
Model of mechanisms of copy-neutral loss of heterozygosity (CNL). (**A**) After an interhomolog recombination event affecting a given allele (i.e., the B allele, but not the A allele), mitotic segregation can result in either a reduction in homozygosity (from Bb in parental cell to BB or bb in daughter cell) or retention of heterozygosity (Bb in both parental and daughter cell). (**B**) A non-disjunction during mitosis can lead to daughter cells either developing an extra paternal chromatid or losing a paternal chromatid. Subsequent elimination of the single-copy chromosome in the former case or duplication of the remaining chromosome in the latter case results in CNN-LOH [7].

In general, there are two types of LOH: one with copy number losses (CNL-LOH) and the other with copy number neutral (CNN-LOH). CNL-LOH is characterized by a deletion of a part or an entire chromosome. CNN-LOH occurrences represent somatic mutations that result in the conversion to homozygosity either through homologous recombination or the duplication of a retained chromosome. In other words, newly formed sections of a chromosome maintain two copies of syntenic loci originating from either the maternal or paternal homolog in the tumor. At the same time, these regions remain biparental in healthy progenitor tissue. The transition to a homozygous state may promote tumor development, especially when it leads to the loss of function of a tumor suppressor gene. However, other genes in the same region may retain two active copies, which helps maintain proper gene dosage. Extensive CNN-LOH arises from at least two distinct molecular mechanisms, including processes relying on homologous recombination and chromosome non-disjunction (Figure 1). The initial mechanism occurs through interhomolog recombination, which, upon mitotic segregation, induces CNN-LOH (Figure 1A). A reduction in homozygosity via homologous recombination mechanisms often serves as an initiating event in tumorigenesis. The second mechanism arises during mitosis due to syntelic attachments and failures in centromere division, which leads both chromatids to segregate towards one pole and none towards the other. To eliminate the homolog or duplicate the chromosome, in order to create a cell with a copy-neutral change (Figure 1B), a second chromosome non-disjunction event is required [4,5,6,7]. These two genetic mechanisms result in CNN-LOH events that typically encompass a substantial portion of a chromosome arm, known as the homologous recombination class, or involve the entire chromosome or chromosome arm, referred to as loss and reduplication. In contrast, chromosome-wide gains and losses are primarily a result of a straightforward chromosome non-disjunction mechanism [8].

In patients with ALL, in addition to the commonly described numerical and structural chromosomal abnormalities, loss of heterozygosity (LOH) changes are also present. Notably, LOH is pervasive, affecting not only non-hematologic tumors but also ALL. This genetic alteration is a ubiquitous feature in the landscape of oncological genetics and deepens our understanding of the disease’s complexity. However, little is currently known about its importance in ALL. LOH has been contributing to leukemogenesis by unmasking pathogenic variants or deregulating gene dosage, thereby affecting apoptosis, cell cycle control, or DNA repair. LOH has been analyzed in the context of ALL to assess its overall frequency and clinical value. Takeuchi et al. proved that individuals with 9p LOH had more frequent central nervous system involvement and T-cell phenotype, suggesting that specific LOH patterns may correlate with disease subtype and clinical characteristics [9].

We hypothesise that specific LOH patterns correlate with distinct clinical features and early indicators of therapeutic efficacy, such as prednisone response and MRD levels, observed during the ALL treatment. This approach would provide a new framework for refining risk stratification in pediatric ALL, beyond conventional cytogenetic markers, and would support the integration of LOH analysis into future diagnostic and therapeutic algorithms. These LOH patterns could define biologically and clinically relevant patient subgroups, providing novel data for more precise risk stratification [10,11,12,13].

Therefore, the main goal of this study is to identify and analyze patterns of LOH, evaluate its frequency and association with clinical characteristics and early response to treatment during the induction phase of the ALL protocol, analyzing the differences in the frequency and type of LOH between B-ALL and T-ALL.

## 2. Materials and Methods

### 2.1. Participants and Study Design

This retrospective study involved bone marrow samples from 853 children younger than 18 years diagnosed with leukemia between 2009 and 2017. Samples were obtained at the time of diagnosis and before the start of treatment. 684 children (80%) were diagnosed with the B-cell acute lymphoblastic leukemia (B-ALL) subtype, while the remaining 169 (20%) were patients with T-cell ALL (T-ALL).

Samples were collected into tubes containing ethylenediaminetetraacetic acid (EDTA). Genomic deoxyribonucleic acid (DNA) was isolated using the QIAamp DNA Blood Mini Kit (Qiagen, Hilden, Germany) and stored at −20 °C until single-nucleotide polymorphism (SNP) array experiments were conducted. The concentration and quality of DNA isolates were assessed using spectrophotometry (NanoDrop One; Thermo Fisher Scientific, Waltham, MA, USA).

Bone marrow samples from all patients underwent cytogenetic analysis with the use of G-banding and fluorescence in situ hybridization (FISH) at the time of diagnosis.

### 2.2. Microarray Analysis

Copy number variation (CNV) analysis was performed using the CytoScan HD microarray (Applied Biosystems, Thermo Fisher Scientific, Waltham, MA, USA). A total of 250 ng of genomic DNA per sample was hybridized, scanned using the GeneChip Scanner 3000 7G (Thermo Fisher Scientific), and analyzed in Chromosome Analysis Suite v4.3 software (ChAS, Thermo Fisher Scientific, Waltham, MA, USA) [14]. A detailed description of all methods can be found in the Supplementary S1. Copy number (CN) states and breakpoints were determined using a hidden Markov model software package. A threshold level of log2 ratio ≥0.3 and ≤−0.5 was used to categorize altered chromosomal regions as CNV gains and losses, respectively. Regions classified as CNVs required a minimum of 50 probes for duplication (gain). Overall, 25 probes for deletion (loss) were applied. Loss of heterozygosity was reported when the length exceeded 15,000 kilobase pairs (kbp). To further identify genes involved in CNV and LOH, two databases were searched: the UCSC database (http://genome.ucsc.edu, accessed on 28 July 2025) and Ensembl (http://www.ensembl.org, accessed on 28 July 2025). Copy number polymorphisms were excluded from microarray results through comparison with the Database of Genomic Variants http://projects.tcag.ca/variation/ (accessed on 28 July 2025).

### 2.3. Experimental Design

Experimental data were divided into two categories for analysis: genome-wide copy number alterations (CNAs) exceeding five megabase pairs (Mbp) and CNAs specific to leukemia-associated regions or genes (leukemia_genes_all_20150505; Fullerton Overlap Map_hg19). A panel consisting of 1276 genes was compiled according to extensive literature on genetic alterations and their involvement in the pathogenesis of ALL. The complete list of genes is provided in Supplementary Table S1.

### 2.4. Criteria of Experimental Group

The experimental group of the study was children with newly diagnosed ALL, treated according to the ALL-IC BFM 2009 and AIEOP-BFM 2017 protocols in 16 centers of the Polish Pediatric Leukemia/Lymphoma Study Group [15,16].

The inclusion criteria were the absence of gene fusion (*BCR*::*ABL1*, *ETV6*::*RUNX1*, *TCF3*::*HLF*, *TCF3*::*PBX1*, any rearrangements of *KMT2A*) and identification of the loss of heterozygosity >15 Mbp. This criterion allowed for the elimination of congenital and polymorphic changes. B-ALL patients with the loss of heterozygosity >15 Mbp comprised 120 (B-ALL LOH+) patients, including 59 (49%) girls and 61 (51%) boys. The T-ALL group consisted of 58 (T-ALL LOH+) patients, including 24 (41%) girls and 34 (59%) boys [17,18].

**Figure 2:**
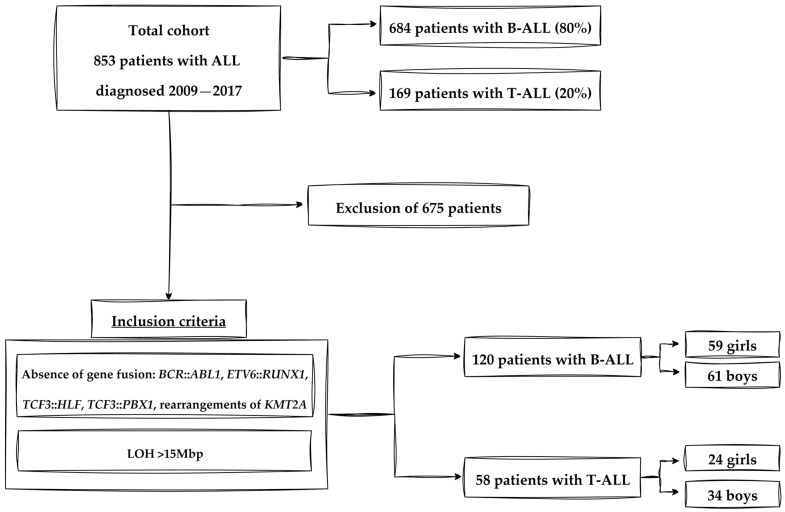
Flowchart of patient selection for the study cohort. From the total cohort of 853 pediatric patients diagnosed with acute lymphoblastic leukemia (ALL) between 2009 and 2017, 684 were diagnosed with B-cell ALL (B-ALL) and 169 with T-cell ALL (T-ALL). After applying exclusion criteria—including the presence of gene fusions (e.g., *BCR*::*ABL1*, *ETV6*::*RUNX1*, *TCF3*::*HLF*, *TCF3*::*PBX1*, and *KMT2A* rearrangements) and low levels of loss of heterozygosity (LOH ≤ 15 Mbp)—a final study group of 178 patients was selected: 120 with B-ALL and 58 with T-ALL. Among the B-ALL group, there were 59 girls and 61 boys; the T-ALL group consisted of 24 girls and 34 boys.

Figure 2 shows a flowchart of patient selection for the study cohort.

The cohort of 120 patients with B-ALL with LOH (B-ALL LOH+) was divided into two distinct groups: hyperdiploid karyotype (*n* = 99, 82.5%) and normal diploid karyotype (ND-LOH+) (*n* = 21, 17.5%).

Distances between individual patients or parameter categories were computed in a multidimensional space defined by selected clinical variables, providing a quantitative measure of their similarity or dissimilarity.

All patients provided written consent for their involvement in the research and for the publication of their data. All procedures conducted as part of the research involving human participants adhered to the ethical guidelines established by the institutional and/or national research committee. These procedures conformed to the principles outlined in the 1964 Helsinki Declaration and its subsequent amendments, or equivalent ethical standards. The study received approval from the ethics committee under reference numbers KE-0254/43/02/2022 and KE-0245/74/03/2023.

### 2.5. Statistical Analysis

#### 2.5.1. Significance Level and Selection of Analytical Methods

In the conducted study, a significance level of α = 0.05 was established, allowing for a 5% risk of Type I error. Analyses were conducted using the R Statistical language (version 4.3.3; R Core Team, 2024; Vienna, Austria) on Windows 11 Pro 64-bit (build 22631).

The selection of Multiple Correspondence Analysis (MCA) followed by Hierarchical Clustering on Principal Components (HCPC) for analyzing pediatric T-cell and B-cell lymphoblastic leukemia cohorts was driven by the need to effectively characterize complex clinical profiles defined by categorical variables, such as gender, age, white blood cell count at diagnosis, percentage of blasts in bone marrow at diagnosis, prednisone response, minimal residual disease (MRD) levels on day 15 and 33, and genetic alterations (e.g., LOH 9p, del *CDKN2A*). These variables are critical for understanding disease behavior and guiding treatment decisions in young patients.

The results are presented through a combination of descriptive statistics, visualizations, and statistical test outputs. Key findings from the MCA and HCPC analyses are illustrated using biplots and two-dimensional cluster visualizations to clearly depict the relationships among clinical and genetic variables, as well as the distribution of patients within identified clusters. Tables summarize the composition of each cluster and highlight the most discriminative variables based on v-test values. Supplementary Materials provide detailed statistical outputs, including inertia values and chi-square test results, to support the robustness of the clustering approach.

#### 2.5.2. Patient Clustering

HCPC was employed to group patients into clusters based on MCA’s principal components, utilizing hierarchical clustering to delineate distinct clinical profiles while maintaining analytical stability through the use of reduced dimensions, thus facilitating the stratification of patients for tailored therapeutic strategies.

Within-cluster inertia, calculated as the sum of squared distances between individual patient data points and their respective cluster centroids, was assessed across multiple partition scenarios. The optimal configuration of the clusters was determined by maximizing the relative loss of inertia. Additionally, the absolute loss of inertia, defined as the difference in inertia between consecutive cluster solutions (i(cluster *n*) − i(cluster *n* + 1)), provided appropriate findings into the balance between cluster granularity and interpretability, ensuring partitions were clinically actionable. Evaluation of the statistical significance of associations between variables and clusters, confirming robust differentiation based on clinical and genetic characteristics essential for prognosis and treatment planning, was conducted by Pearson chi-square tests. A chi-squared goodness-of-fit test confirmed non-random LOH distribution (*p* < 0.001). Furthermore, the v-test was used to measure category-specific differences, quantifying the prevalence of specific clinical and genetic features within clusters compared to the overall cohort, with higher absolute v-test values indicating stronger associations relevant to patient outcomes. Visual validation was achieved through biplots and two-dimensional cluster visualizations, which illustrated the distribution of patients and parameters, reinforcing the separation of clusters and their alignment with clinical expectations.

#### 2.5.3. Role and Interpretation of Clusters and Dimensions

A dimension in MCA represents a principal component derived from the transformation of categorical variables into a lower-dimensional space, capturing the underlying structure of associations among variables. In the T-cell leukemia study, MCA transformed 11 categorical variables (e.g., gender, prednisone response, del *CDKN2A*, LOH 9p) into an 18-dimensional framework, while in the B-cell study, 9 variables (e.g., LOH 9/9p, hyperdiploidy, del *CDKN2A*) were mapped into a 23-dimensional framework, with the variance fully explained in each case.

A cluster, in contrast, refers to a group of patients identified through HCPC, where individuals are grouped based on their similarity across the principal components derived from MCA. In both studies, HCPC applied to MCA’s principal components yielded three clusters: 12, 36, and 10 patients in the T-cell cohort, and 30, 54, and 36 patients in the B-cell cohort. These clusters explained 26.3% of the total variance in the T-cell study (14.7% in Dimension 1, 11.6% in Dimension 2) and 22.5% in the B-cell study (12.1% in Dimension 1, 10.4% in Dimension 2).

The difference between dimensions and clusters lies in their roles and outputs within the analytical pipeline. Dimensions are analytical constructs that reduce the complexity of categorical data into orthogonal axes, each explaining a portion of the variance and highlighting variable associations.

The data analyzed are multidimensional, which means that they can be represented in a ten-dimensional space to fully explain all their variance. Each dimension is made up of the original variables of the analyzed study, creating values for Dimension 1 and Dimension 2. They allow the interpretation of the study results and their impact on the division of patients into clusters. The Dimensions create new categories independent of each other. The Dimensions are visualisations of multivariate data in the main components of statistical analysis. Dimensions from MCA are statistical data for HCPC.

#### 2.5.4. Analysis Objective and Input Variables

The statistical analysis carried out aimed to identify these dimensions and then visualize the clusters in these spaces.

In our study, we employed MCA followed by HCPC to explore complex relationships among both genetic and clinical variables. The input variables included the presence or absence of LOH in several categories, copy number variation of the *CDKN2A* gene, ploidy status, age, count of blasts in bone marrow, early response to corticosteroids, and minimal residual disease (MRD) levels on days 15 and 33. These categorical variables were analyzed simultaneously to reduce dimensionality and to identify new synthetic Dimensions that best represent the structure of the data.

These dimensions were then used for unsupervised clustering using HCPC, which grouped patients based on shared genetic and clinical profiles, independently of predefined risk groups. This approach allowed us to uncover novel patient subgroups characterized by specific combinations of molecular alterations and treatment response patterns.

#### 2.5.5. Clustering Results: B-ALL and T-ALL Cohorts

The algorithm employing HCPC to MCA identified an optimal number of three clusters in B-ALL with LOH: cluster 1 comprised 30 patients (25%), cluster 2 included 54 patients (45%), and cluster 3 contained 36 patients (30%) (Supplementary Figure S8).

The composition of the clusters in T-ALL was as follows: Cluster 1 comprised 12 patients (21%), Cluster 2 comprised 36 patients (62%), and Cluster 3 comprised 10 patients (17%) (Supplementary Figure S16).

A full description of the statistical analyses is presented in Supplementary S1.2 and S1.3, including the results of the chi-square test, clustering, and specific genetic alterations in the subgroups. Supplementary S2.3.1 is the statistical description of the results in the B-ALL group, and Supplementary S2.3.3 is the statistical description of the results in the T-ALL group. The results presented in Files S2 and S3 were subjected to statistical analysis. 

## 3. Results

We selected 120 patients with B-ALL and 58 patients with T-ALL, all with LOH >15 Mbp, from an initial cohort of 853 children participating. Table 1 shows the clinical and laboratory data of patients with B- and T-ALL.

### 3.1. Genetics Characteristics of B-ALL with LOH

**Figure 3:**
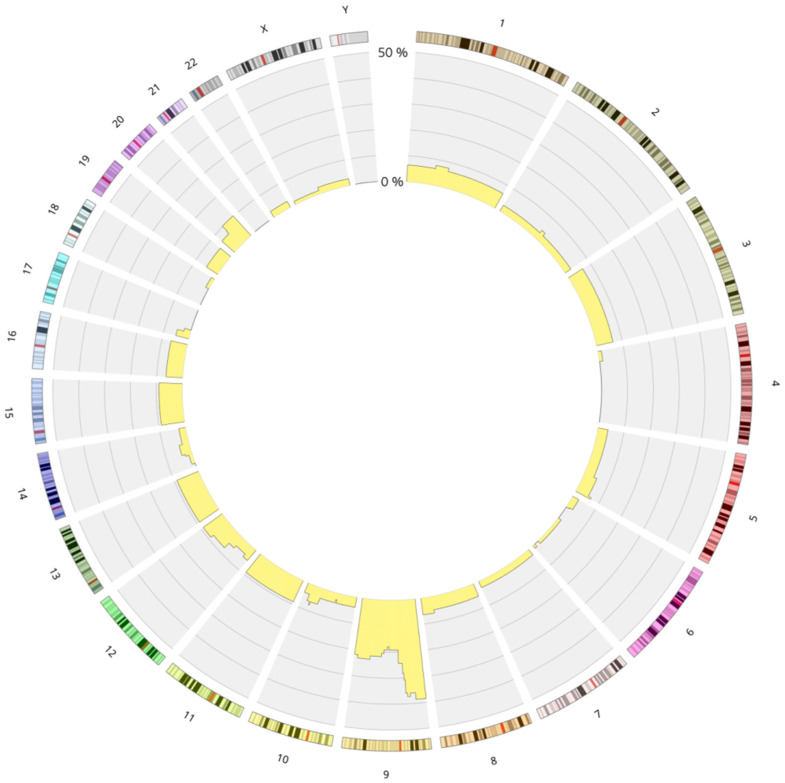
Circos plot presenting the landscape of the loss of heterozygosity in B-ALL divided into chromosomes. Circos plot showing the distribution of loss of heterozygosity (LOH) across chromosomes in pediatric B-ALL patients with LOH (*n* = 120). Yellow bars represent the percentage of patients with LOH in each genomic region (scale: 0–50%). Chromosome 9 shows the highest burden of LOH. Abbreviations: LOH—loss of heterozygosity; B-ALL—B-cell acute lymphoblastic leukemia.

Figure 3 and Supplementary Figure S1 and Table S2 represent the distribution of LOH in the whole group of B-ALL cases.

The chromosome 9 was the most affected by the LOH events in the hyperdiploid group (*n* = 49, 49.5%). In this group, most patients (*n* = 33, 67%) exhibited LOH involving the entire chromosome, while the remaining patients (*n* = 16, 33%) showed segmental LOH. Among those with segmental LOH, 12 patients (*n* = 12, 24.5%) exhibited changes restricted to the short arm (9p), and 4 patients (*n* = 4, 8%) exhibited changes limited to the long arm (9q). The remaining hyperdiploid group comprised 50 patients (50.5%) presenting LOH on other chromosomes without chromosome 9 in the form of LOH on the entire chromosome or segmental. The most frequent alterations involved LOH affecting entire chromosomes only (*n* = 29, 58%), observed exclusively in the high-hyperdiploid group. Segmental LOH was observed in both high and low hyperdiploidy cases (*n* = 15, 30%). The last pattern was the presence of both segmental LOH and LOH involving entire chromosomes (*n* = 6, 12%), with these patients predominantly belonging to the high-hyperdiploidy group. The normal diploid patients exhibited a distinct genetic profile compared to those with hyperdiploidy. One-third of patients (*n* = 7, 33%) presented intrachromosomal amplification of chromosome 21 (iAMP21), which was accompanied by a loss of heterozygosity in the 21q21.1q22.3 region. Four of them presented additional LOH in the 12q14.1q24.33 region containing the *SH2B3* gene. The only LOH events were observed in this subgroup. Isolated LOH events across different chromosomes and LOH concerning the short arm region of chromosome 9 were characteristic of the remaining patients (*n* = 14, 66%) in the normal diploid group. Six of them had the deletion of the *CDKN2A* and *CDKN2B* genes.

**Figure 4:**
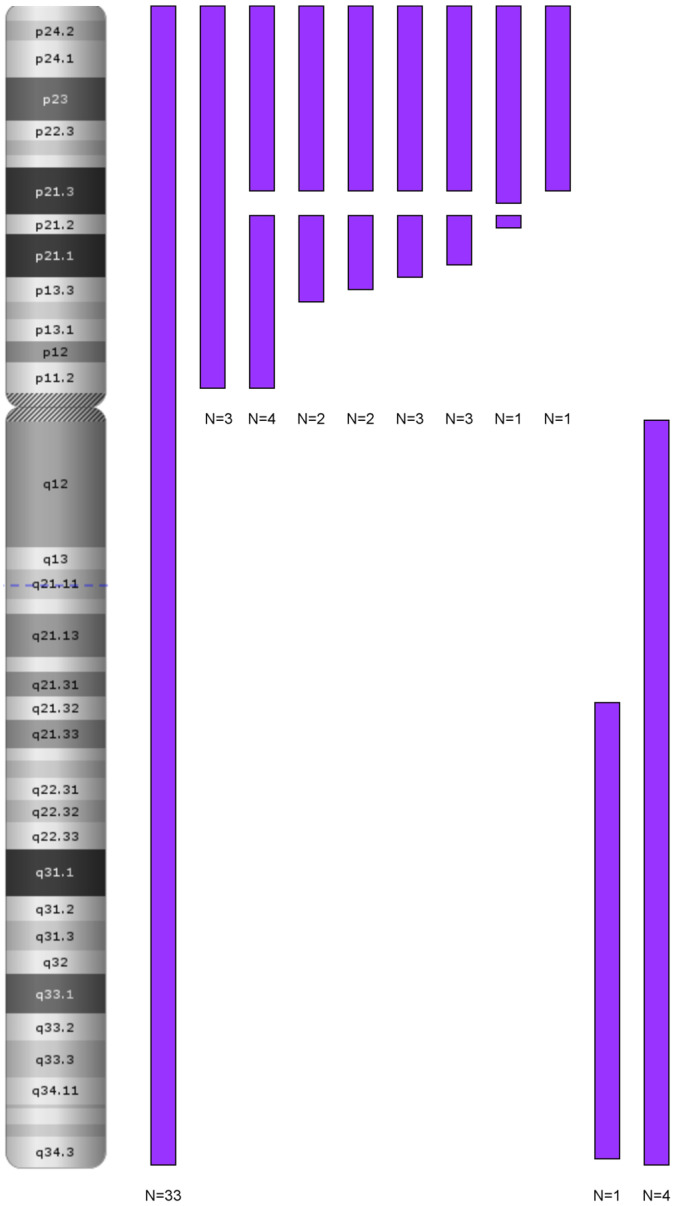
LOH in chromosome 9 in the B-ALL group. Genomic locations of loss of heterozygosity (LOH) events on chromosome 9 in pediatric B-ALL patients (*n* = 120). Each vertical bar represents a distinct LOH event mapped to the corresponding chromosomal band. The majority of LOH events affect the short arm (9p), particularly the CDKN2A/2B locus (9p21.3). Fewer events are observed on the long arm (9q). LOH mapping was performed using high-resolution SNP microarrays. No overlapping whole-chromosome LOH was detected in this group. Abbreviations: LOH—loss of heterozygosity; B-ALL—B-cell acute lymphoblastic leukemia.

The B-ALL cohort demonstrates considerable genetic diversity, with chromosome 9 being the most frequently altered, primarily through LOH and deletions in the *CDKN2A* gene region. The most common LOH alteration was observed across the entire chromosome 9, followed by LOH in the 9p region, with LOH in the 9q region being the least frequent (Figure 4).

Regarding *CDKN2A* deletions, the frequencies were as follows: monoallelic deletions, biallelic deletions, and duplications. In Table 2, we presented characteristics of LOH on chromosome 9 and anomalies of *CDKN2A* in the B-ALL group.

### 3.2. Clinical Characteristics of B-ALL with LOH

The analysis indicated that loss of heterozygosity at chromosome 9p and *CDKN2A* deletions (del *CDKN2A*) were significantly associated with both Dimension 1 and Dimension 2. Hyperdiploidy showed a strong association with Dimension 1. Clinically, the response to prednisone correlated with Dimension 1, while minimal residual disease (MRD) on day 15 correlated with Dimension 2. Age exhibits a weak association with both dimensions. Other variables did not show significant links with the specified dimensions, Supplementary Tables S3–S5 and Figure S2.

Patients with similar clinical characteristics or disease trajectories, such as LOH 9/9p, LOH segments, age, level of MRD on day 15, response to prednisone, deletion of the *CDKN2A* gene, hyperdiploidy, and percentage of blasts in bone marrow at diagnosis, were positioned closer together on the factor map, as depicted in Supplementary Figure S3.

The distribution of patients across the first and second dimensions, categorized by individual parameters, was illustrated in Supplementary Figures S4–S7.

Cluster 1 has been identified as a subgroup that is distinguished by a high prevalence of genetic alterations. Notably, 88% of patients in this cluster exhibit LOH on chromosome 9 or its short arm (LOH 9/9p). All patients in this cluster are characterized by a monoallelic deletion within the *CDKN2A* gene. Only 34% of patients in Cluster 1 exhibit high hyperdiploidy. Within cluster 1, no statistically significant influence of clinical factors was observed, but only a highly significant influence of genetic factors (Supplementary Table S5).

Cluster 2 presents a more favorable genetic and clinical profile, such as high hyperdiploidy, good response to therapy (low levels of MRD), and absence of LOH 9/9p. A hallmark of this cluster is the absence of LOH 9/9p in 75% of patients. Moreover, 82% of Cluster 2 patients retain a normal *CDKN2A* status. Another defining feature is the complete presence of *CDKN2A* duplication in all Cluster 2 patients, a genetic alteration that, while uncommon in the whole patients’ group, appears to characterize this subgroup. Hyperdiploidy is observed in 57% of Cluster 2 patients. Only 2% of patients in this cluster have MRD levels ≥10% on day 15, compared to 9% globally, suggesting a robust initial response to therapy. Demographically, Cluster 2 skews toward younger patients (59% ≤ 5 years) (Supplementary Table S5).

Cluster 3 represents a poor outcomes profile with a characteristic genetic and clinical profile. The defining genetic feature of this cluster is the common presence of biallelic *CDKN2A* deletions in 100% of patients. LOH with a deletion-related “gap” in the *CDKN2A* gene encompassing the short arm of chromosome 9 occurs in all patients in Cluster 3. Common genomic instability in Cluster 3 is underlined by the high incidence of segmented LOH (94%) and low hyperdiploidy (92%). The chromosomal profile of Cluster 3 is dominated by diploid karyotypes, occurring in 86% of patients, which is in sharp contrast to the high hyperdiploidy. A significant proportion of Cluster 3 patients show a poor response to prednisone (57%). Bone marrow infiltration at diagnosis is also notable, with 39% of patients showing blasts >91%. In contrast to Cluster 2, which includes a larger proportion of younger patients, only 22% of Cluster 3 patients are ≤5 years old (Supplementary Table S5).

A summary of the deletions and duplications in B-ALL was provided in Supplementary Figures S9 and S10.

**Figure 5:**
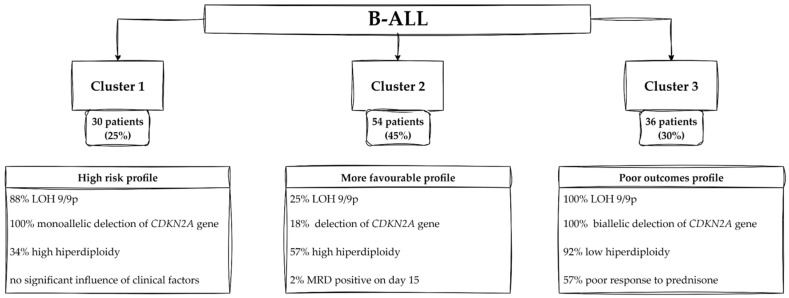
Characteristics of Clusters in B-ALL. Abbreviations: LOH—loss of heterozygosity; B-ALL—B-cell acute lymphoblastic leukemia; MRD—minimal residual disease.

The characteristics of Clusters in B-ALL are presented in Figure 5.

### 3.3. Genetic Characteristics of T-ALL with LOH

The T-ALL LOH+ subtype is characterized by a significantly lower frequency of chromosomal copy number alterations (CNA). Unlike for B-ALL LOH+, we did not observe hyperdiploid karyotypes (Supplementary Figure S11). Most individuals presented a karyotype with microdeletions and microduplications or a karyotype with segmental deletions or duplications.

**Figure 6:**
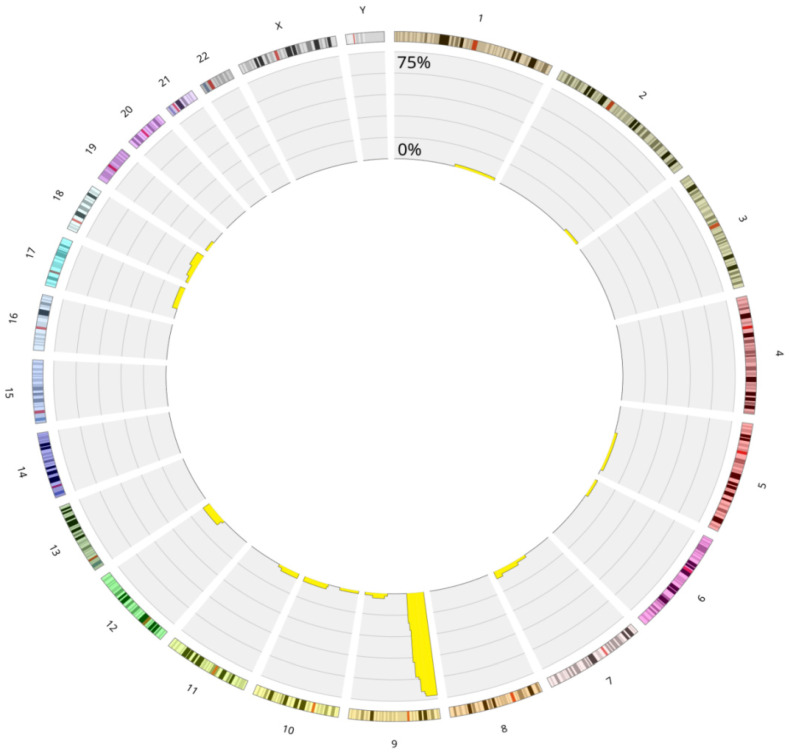
Circos plot presenting the landscape of the loss of heterozygosity in T-ALL divided into chromosomes. Circos plot showing the distribution of loss of heterozygosity (LOH) across chromosomes in pediatric T-ALL (*n* = 58). Yellow bars indicate the percentage of patients with LOH in each genomic region (scale: 0–75%). Abbreviations: LOH—loss of heterozygosity; T-ALL—T-cell acute lymphoblastic leukemia.

In terms of LOH occurrence, the group was notably homogenous, with most patients displaying LOH on the short arm of chromosome 9 (*n* = 44, 76%). LOH was also detected on other chromosomes, but these changes were segmental and distributed randomly throughout the genome (Figure 6). The extent of LOH on chromosome 9p varied among patient groups, ranging from the smallest affected region, 9p24.3p21.3 (192,128–20,407,934), to the largest, 9p24.3p13.2 (192,128–36,815,056), with certain cases exhibiting LOH across the entire 9p arm.

**Figure 7:**
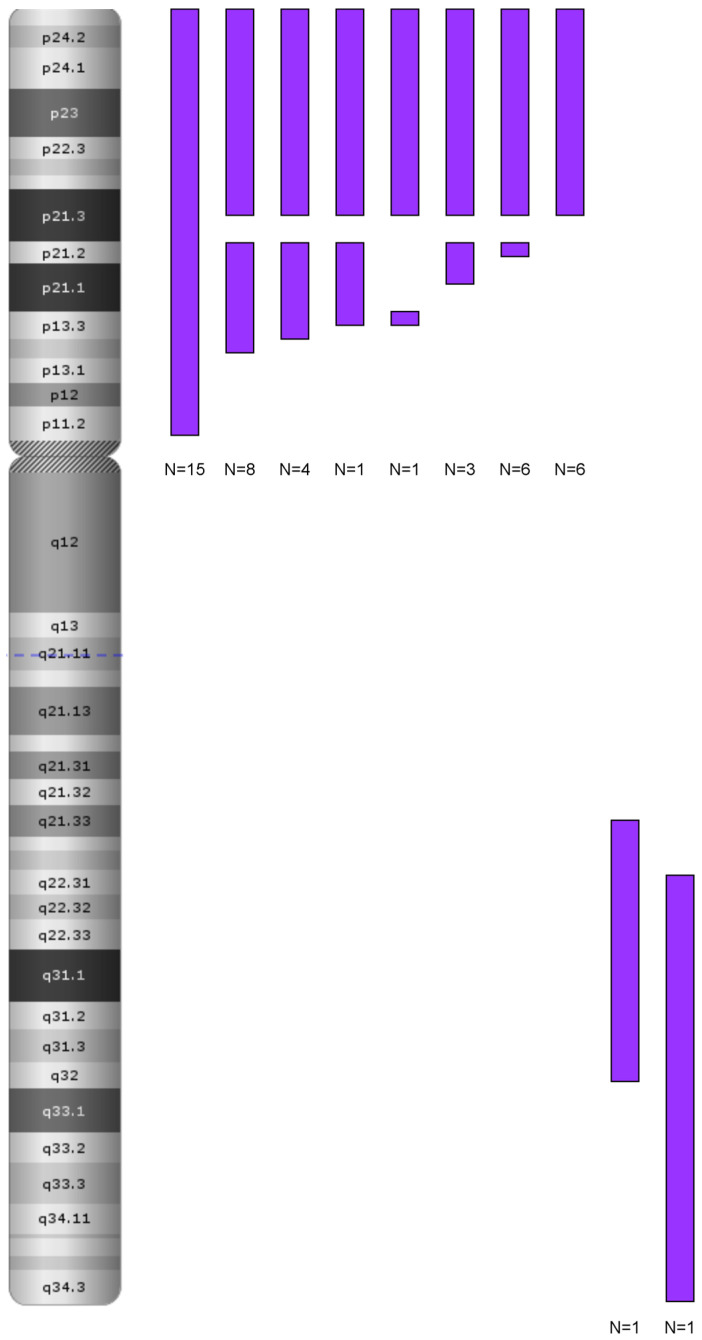
LOH in chromosome 9 in the T-ALL group. Distribution of loss of heterozygosity (LOH) on chromosome 9 in pediatric T-ALL patients (*n* = 58). Each vertical purple bar represents an individual LOH event mapped to a chromosomal band. LOH was predominantly localized to the short arm (9p), including the CDKN2A/2B locus (9p21.3), while fewer events were observed on the long arm (9q). Data were obtained from SNP-array profiling. LOH was mostly segmental; no whole-chromosome LOH was detected. Abbreviations: LOH—loss of heterozygosity; T-ALL—T-cell acute lymphoblastic leukemia.

The largest group of patients was characterized by LOH separated by deletions of various sizes within the *MLLT3*, *IFNA*, *MTAP*, and *CDKN2A/2B* genes. The second group had LOH involving the entire 9p arm, while the smallest group showed smaller segmental LOH within 9p (Figure 7).

The characteristic pattern of deletions primarily involved genes that separated the LOH regions on 9p, with *CDKN2A* being the most frequently deleted gene. Among these, biallelic deletions accounted for, while monoallelic deletions were observed in. In Table 3, we present characteristics of LOH on chromosome 9 and anomalies of *CDKN2A* in the T-ALL group.

### 3.4. Clinical Characteristics of T-ALL Group

Analysis indicates that LOH on chromosome 9p, *CDKN2A* deletions, and early treatment response are significantly associated only with Dimension 1. In Dimension 2, neither LOH on 9p nor follow-up duration exhibited significant associations (Supplementary Tables S6 and S7 and Figure S12).

Patients with similar clinical characteristics or disease trajectories, such as LOH 9p, age, gender, level of MRD on day 15 and 33, response to prednisone, deletion of the *CDKN2A* gene, relapse, percentage of blasts in bone marrow at diagnosis, and white blood cell count at diagnosis, are positioned closer together on the factor map. The visualization of the clusters in a two-dimensional space on Supplementary Figure S13 accounted for 26% of the total variance in the data, with 15% explained in the first dimension and 12% in the second dimension. The distribution of patients across the first and second dimensions, categorized by individual parameters, is illustrated in Supplementary Figures S14 and S15.

In terms of genetic alterations, Cluster 1 is characterized by complete loss of heterozygosity at the 9p, which also means complete loss of heterozygosity at the *CDKN2A* gene. Specifically, patients in Cluster 1 show a notably high prevalence of certain features, such as low levels of MRD on days 15 and 33, good response to treatment, and exhibition of LOH at the *CDKN2A* gene. The percentage of patients showing flow cytometry-MRD (MRD FMC) at 15 days with levels less than 0.1% is strikingly high at 90%, compared to a global prevalence of only 20%. A response to prednisone in this cluster is predominantly classified as “good”, with 33% of patients responding positively, in sharp contrast to the global response rate of 62%. The data also indicate that 67% of patients in Cluster 1 are MRD detected by polymerase chain reaction (MRD/PCR) negative at 33 days, compared to only 15.5% globally.

In terms of genetic features, the presence of LOH at the *CDKN2A* locus is much more prevalent in Cluster 1, with 100% of patients exhibiting this alteration, while only 5% are observed globally. Furthermore, the prevalence of patients with MRD FMC levels of 10% or more is also low in Cluster 1, at 8%, which is in stark contrast to the global prevalence of 45%. In Cluster 1, none of the patients is classified as having a poor response. In comparison, the global sample indicates a poor response rate of 38% (Supplementary Table S8).

Patients in Cluster 2 present a different clinical and genetic profile, characterized by a high prevalence of biallelic deletion of *CDKN2A*, with 80% of patients exhibiting this alteration, compared to 60% in the global sample. Furthermore, the positive MRD/PCR results at 33 days are also noteworthy, with 78% of patients in Cluster 2 testing positive and surpassing the global prevalence of 64%. Additionally, the prevalence of MRD FMC levels between 0.1% and 10% is strikingly high in Cluster 2 at 86%, in contrast to the global prevalence of 36%. The gender distribution reveals that 76% of patients in Cluster 2 are male, compared to 59% in the global sample. Conversely, the analysis of negative v-test results provides further insights. The absence of loss of heterozygosity at the 9p locus is observed in 14% of patients in Cluster 2, which is lower than the global prevalence of 24%. The negative MRD/PCR result at 33 days is notable, with 6% of patients in Cluster 2 achieving this outcome, compared to 15.5% globally. Prevalence of female patients in Cluster 2 stands at 28%, significantly less than the global prevalence of 41%. The prevalence of MRD FMC levels lower than 0.1% is notably low in Cluster 2 at 3%, compared to 19% globally (Supplementary Table S8).

The clinical profile of patients in Cluster 3 is characterized by a high prevalence of a normal profile of *CDKN2A*, a notable absence of LOH at 9p, and a concerning rate of MRD. A characteristic feature of this cluster is that 100% of patients exhibit a normal *CDKN2A* profile, which sharply contrasts with the global average of 14%. In 64% of patients, there was no loss of heterozygosity at 9p, which is a percentage significantly higher than the average of the research group (24%). The assessment of MRD at 15 days post-treatment reveals that 31% of patients in Cluster 3 have MRD levels of 10% or greater. This prevalence is lower than the global average of 45%. In terms of treatment response, 50% of patients in Cluster 3 have an MRD/PCR result indicating no detectable markers at 33 days, which is significantly higher than the average of 21%. The clinical profile includes a notable prevalence of patients with WBC counts of 117.86 × 10^3^/μL or less, observed in 28% of individuals, which is lower than the global average of 50%. The prevalence of positive MRD/PCR results at 33 days post-treatment is observed in 30% of patients, which is significantly lower than the global average of 64%. The absence of biallelic deletion of *CDKN2A*, with none of the patients in Cluster 3 exhibiting this genetic alteration, contrasts sharply with the global prevalence of 60% (Supplementary Table S8).

**Figure 8:**
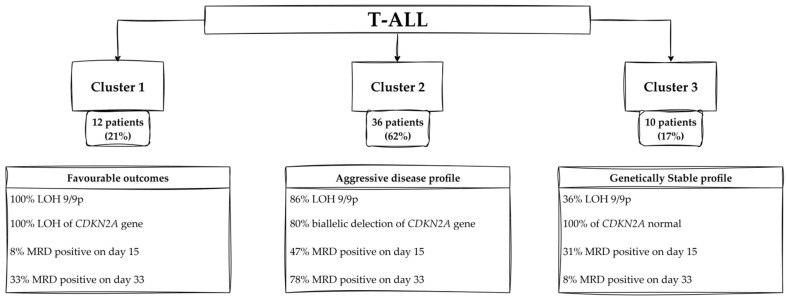
Characteristics of Clusters in T-ALL. Abbreviations: LOH—loss of heterozygosity; T-ALL—T-cell acute lymphoblastic leukemia; MRD—minimal residual disease.

A summary of the clinical and genetic characteristics of Clusters in T-ALL is presented in Figure 8.

The supplement provides a detailed description of the chi-square test results, clustering, and specific genetic alterations in the subgroups. The results of all molecular karyotypes were systematically compiled in File S1. The results presented in Files S2 and S3 revealed significant associations. These associations were described in the clinical characteristics of both groups.

### 3.5. Chromothripsis

In the B-ALL group, the phenomenon of chromothripsis was found in two patients. The first patient exhibited chromothripsis within chromosome 7 and presented a high hyperdiploid karyotype with additional 4, 5, 8, 14, 18, and X chromosomes and two additional copies of chromosome 21. Moreover, the only loss of heterozygosity was LOH within the short arms of chromosome 9, but a deletion was also found in the *IKZF1* gene (region 7p13p12.1) and a deletion of the 12p13.2p12.3 region in the *ETV6* gene. The second patient presented with chromothripsis within the chromosome 12 region. He was also diagnosed with a deletion within chromosome 3, LOH in the region 5p, a duplication of the region 5p, deletions within the chromosome 8, a deletion in the *CDKN2A*, *CDKN2B*, and *PAX5* genes in 9p, monosomy of chromosome 13, a deletion in the *TP53* gene region 17p, and additional copies of chromosomes 21 and X (Supplementary Table S9).

In the T-ALL group, chromothripsis at chromosome 7 was observed in one patient. The karyotype was characterized by many aberrations. Changes like chromothripsis were also noticeable on other chromosomes and concerned chromosomes 1 and 9, on which the following deletions were also found: a biallelic deletion of *CDKN2A* and *CDKN2B* genes, a deletion within chromosome 5 5q23.3q35.3(130,287,637_180,719,789), a deletion in the *ETV6* gene on chromosome 12, LOH in the 12q24.11q24.33(111,548,325_133,778,166) region, and an intrachromosomal deletion of the 13q13.3q21.33(40,010,663_70,747,804) region in the *RB1* gene (Supplementary Table S9).

## 4. Discussion

To the best of our knowledge, this is the first comprehensive analysis of LOH in pediatric leukemia. The aim of this study is to identify and analyze patterns of loss of heterozygosity (LOH), assess their frequency, and evaluate their association with clinical characteristics and early treatment response during the induction phase of the ALL protocol. This study demonstrates notable genetic and clinical diversity among pediatric ALL patients, with chromosome 9p LOH and *CDKN2A* deletions being the most significant alterations. Three distinct genetic clusters were identified. Cluster 1: Favorable profile—complete 9p LOH, 100% *CDKN2A* deletion, low MRD levels, good early response. Cluster 2: Intermediate/poor response—high biallelic *CDKN2A* deletion, elevated MRD, mostly male patients. Cluster 3: Biologically distinct—normal *CDKN2A*, no 9p LOH, variable MRD status, poor genetic risk markers absent. In T-ALL, LOH was predominantly limited to chromosome 9p, with CDKN2A as the most frequently affected gene, but no hyperdiploidy was observed. Overall, LOH at 9p and *CDKN2A* deletions emerged as critical markers for risk stratification and treatment response prediction in pediatric ALL.

Although prior studies have described the frequency and chromosomal distribution of LOH in pediatric ALL, the clinical significance of these findings—particularly in relation to early treatment response measured by MRD—has remained incompletely understood. Our study bridges this gap by providing a comprehensive analysis of LOH patterns in relation to MRD clearance and other prognostic markers, demonstrating that specific configurations of LOH, especially on chromosome 9p, have a consistent and measurable impact on disease trajectory.

Compared to our study, Chambon-Pautas et al. detected LOH at one or more loci in 64% of 63 patients. 9p (36%), 12p (31%), 20q (15%), 6q (12%), 5p (10%), and 10p (10%) were the most commonly involved chromosomal arms [19]. In the analysis of LOH within known or putative regions harboring tumor suppressor genes in 29 patients (24 pre-B and five of T-cell lineage), the highest frequencies of LOH on chromosomes 9p and 12p were observed in 29% and 32% of informative cases, respectively. Other LOH were noted less often on chromosomes 6p, 6q, 9q, 17p, and 17q, without LOH at chromosomes 3p, 5q, 11p, 11q, 13q and 18q [20]. This finding is only partially consistent with our results, as in our study, LOH on chromosomes 12 and 20 was more commonly observed to involve entire chromosomes.

### 4.1. B-ALL

#### 4.1.1. LOH 9/9p and Deletion of CDKN2A/2B

##### LOH and Biallelic Deletion of CDKN2A

A study confirming the occurrence of LOH most frequent on chromosome 9 found four patients with biallelic deletion 9p21.3 (three of them had LOH) of the tumor suppressor gene cluster (*CDKN2A*, *CDKN2BAS1*, *CDKN2B*, and *DMRTA1*), which is fully consistent with our research [21]. Another study identified CN-LOH on chromosome 9p in 9% of cases and deletions of *CDKN2A/2B* in 36%, emphasizing the pivotal role of this region in the pathogenesis of B-ALL [22]. Similarly, our study revealed comparable rates of LOH restricted to the short arm of chromosome 9 (9p) and deletions of *CDKN2A/2B* [23].

##### Role in Clonal Evolution of LOH on the Entire Chromosome 9

Notably, we also observed LOH on the entire chromosome 9 in an additional 27.5% of cases, which suggests a greater role of this chromosome in clonal evolution. Biallelic deletion of *CDKN2A/2B* in pediatric B-ALL is an important prognostic factor, associated with poorer treatment outcomes, higher relapse rates, and lower event-free survival. Our findings support these observations, suggesting that this alteration may act as a secondary change contributing to B-ALL progression [23].

##### LOH in Other Chromosomal Regions: 6p and 12p/q

A separate study highlighted that LOH on the human leukocyte antigen (HLA) gene locus (6p) may significantly influence the donor search in hematopoietic stem cell transplantation [24]. LOH in the chromosomal region 12p12—13 in at least one locus was described in the group of 11 of 61 patients with ALL (52 B-ALL, nine T-ALL) [25]. It is suggested that LOH can be measured at the onset of the disease and be used as a prognostic factor (for poor outcomes in B-ALL, but not in T-ALL) [26]. In our study, LOH on chromosome 6 was rare because it was observed in only two patients, similar to LOH within the p arm of chromosome 12. Reported the presence of loss of heterozygosity (LOH) in the *SH2B3* gene, which was associated with *RUNX1* (iAMP21) gene amplification. Our findings are in agreement with and further substantiate these observations, as we also identified LOH in the 12q14.1q24.33 region, encompassing the *SH2B3* gene, in four out of seven patients with iAMP21 in our cohort [27].

##### The Role of LOH 9p in the Pathogenesis of ALL

LOH of the short arm of chromosome 9, including the *CDKN2A* gene, is known in the literature as one of the most frequent aberrations in pediatric ALL. Study suggested that such deletion is a significant secondary abnormality in childhood ALL, strongly correlated with phenotype and genotype, while CNN-LOH without *CDKN2A* inactivation indicates the presence of other relevant genes in this region, which is consistent with our results [28].

Our findings are partly consistent with the observations of Sandmann et al., who highlighted the critical role of alterations on chromosome 9 in T-ALL. Frequent CNVs and LOH in this region, involving tumor suppressor genes such as *CDKN2A/2B* and *MTAP*, have been described as early events in clonal evolution. Similarly, in our cohort, we observed frequent deletions and LOH on chromosome 9p, particularly affecting *CDKN2A/2B*. These results underline the importance of this chromosomal region in the pathogenesis of T-ALL and indicate its early involvement in disease progression [29].

#### 4.1.2. Clinical Implications of LOH 9/9p

In a clinical context, our study describes the prognostic implications of loss of heterozygosity in childhood leukemia, identifying three subgroups of patients with distinct genetic and clinical profiles. The results highlight the key role of LOH, particularly on chromosome 9p, in influencing disease outcome, which provides new insights into the genetic heterogeneity of childhood leukemia. On the basis of our research, three clusters were generated for each type of leukemia.

##### Profiles of Patients—Cluster 1

Cluster 1, focused on B-ALL (high-risk subgroup with LOH 9p), stands out as a high-risk subgroup with a striking predominance of LOH on chromosome 9p (88% of patients). This region contains key tumor suppressor genes, including *CDKN2A*, *CDKN2B*, and *MTAP*, with all patients in this cluster displaying monoallelic deletions of *CDKN2A*. The strong association between LOH 9p and poor outcome is consistent with multiple studies [30,31]. Interestingly, high hyperdiploidy, a feature typically associated with good prognosis [32], is underrepresented in this cluster (34%). This suggests that ubiquitous LOH 9p deletions and associated *CDKN2A* may outweigh the protective effects of hyperdiploidy, underlining the adverse impact of LOH.

##### Profiles of Patients—Cluster 2

In contrast, Cluster 2 is characterized by a more favorable prognosis, with 75% of patients lacking LOH 9p. The preservation of chromosome 9p integrity, including intact *CDKN2A* in 82% of patients, likely contributes to the better outcomes observed in this group [33]. Curiously, *CDKN2A* duplication, a rare genetic alteration in the entire cohort, is present in all patients in Cluster 2. This duplication may enhance tumor suppressor activity, potentially reducing the risk of disease progression. The lower incidence of MRD and the predominance of younger patients (<5 years of age) further suggest a favorable prognosis for this subgroup. These results are consistent with previous studies [12], particularly those identifying *CDKN2A* alterations as key prognostic factors in childhood leukemia, reinforcing the notion that preserved *CDKN2A* function correlates with better clinical outcomes.

##### Profiles of Patients—Cluster 3

Cluster 3 of B-ALL was categorized as a high-risk subgroup with extensive genomic instability. Previous research identified loss of heterozygosity (LOH) on chromosome 9p and biallelic deletions of *CDKN2A* as critical factors for aggressive disease in childhood leukemia. Our findings support these results, as Cluster 3 represents a poor outcomes subgroup dominated by extensive LOH and genomic instability. All patients in this cluster exhibit LOH involving chromosome 9p and biallelic deletions of *CDKN2A*, which result in the complete loss of function of the tumor suppressor gene. This genetic profile underscores the role of *CDKN2A* alterations in promoting poor clinical outcomes, which further underscores the association between 9p LOH and aggressive disease. In contrast to Cluster 2, low hyperdiploidy—a hallmark of Cluster 3—has been associated with impaired genomic stability and poor outcomes. The predominance of diploid karyotypes and clinical markers, such as poor response to prednisone and significant bone marrow infiltration, further underscores the aggressive nature of this subgroup. Intriguingly, the identification of *CDKN2A* duplications as a hallmark of favorable prognosis in Cluster 2 provides novel insights. This finding suggests that *CDKN2A* status plays a dual role: its deletion bears a high risk, whereas its duplication may enhance the suppressive effect.

In B-ALL, both Cluster 1 and Cluster 3 are associated with a poor prognosis and demonstrate a high prevalence of LOH involving chromosome 9 or its short arm (9p). In Cluster 1, 94% of patients exhibit LOH 9/9p, closely associated with the complete deletion of *CDKN2A*, a key tumor suppressor gene that regulates the cell cycle. The co-occurrence of *CDKN2A* deletions and LOH establishes a dual pathway for disease progression, whereby unchecked proliferation and resistance to therapy are promoted. Similarly, in Cluster 3, LOH 9p is observed in all cases, and its association with diploid karyotypes and other markers of chromosomal instability compounds the aggressive nature of the disease. These high-risk clusters illustrate how LOH in B-ALL can serve as a biomarker of genetic instability and a driver of poor outcomes. Conversely, Cluster 2 in B-ALL, associated with more favorable outcomes, exhibits a markedly different LOH profile. A mere 25% of patients in this cluster display LOH 9/9p, indicative of a diminished burden of chromosomal instability. The absence of pervasive LOH correlates with the preservation of *CDKN2A* and other genetic features that maintain cellular control mechanisms. This stability is reflected in the clinical outcomes of Cluster 2, where patients demonstrate lower levels of MRD and better responses to therapy.

### 4.2. T-ALL

This study sheds light on the prognostic significance of LOH in pediatric T-ALL through identifying three distinct patient clusters with unique genetic and clinical characteristics. These findings underscore the role of LOH, particularly on chromosome 9p, as a crucial factor influencing disease outcomes and providing insights into the genetic landscape of T-ALL.

#### 4.2.1. Profiles of Patients—Cluster 1

The first cluster, referred to as the favourable outcomes’ subgroup with LOH at 9p, is uniquely characterized by a complete loss of heterozygosity at both the *CDKN2A* and 9p loci in all patients. This prevalence, markedly exceeding the global averages of 5% and 76%, respectively, suggests a critical role for these loci in defining the cluster’s clinical profile. Associated alterations in these regions with disease progression and poorer survival, our findings reveal a different perspective. Despite their high-risk genetic profile, 90% of Cluster 1 patients achieve MRD levels below 0.1% by day 15, significantly surpassing the global prevalence of 20% [34]. Similarly, by day 33, 67% achieve negative MRD/PCR results, compared to a global rate of 15.5%. This remarkable response aligns with the hypothesis of Wang et al. [35], who proposed that specific genetic alterations traditionally linked to worse outcomes might enhance sensitivity to targeted therapies. Additionally, 33% of these patients were classified as “good responders” to corticosteroids, with no poor prednisone responders reported, in contrast to the global rate of 38% for poor response. These findings align with a study, which identified corticosteroid sensitivity as a robust prognostic marker in T-ALL [36]. The interplay of LOH and treatment-related factors suggests that the specific configuration of LOH in this cluster facilitates effective therapeutic responses.

#### 4.2.2. Profiles of Patients—Cluster 2

Cluster 2, identified as an aggressive disease profile with High MRD, exhibits a different genetic and clinical landscape. This group is defined by a high prevalence of biallelic deletions at the *CDKN2A* locus (80%), which exceeds the global rate of 60%, and persistent residual disease. By day 15, 86% of patients exhibit MRD levels between 0.1% and 10%, while only 3% achieve MRD levels below 0.1%, compared to global averages of 36% and 19%, respectively. Furthermore, 78% remained MRD/PCR positive by day 33, reflecting the cluster’s aggressive disease profile. Notably, LOH at 9p is less prevalent in this group (14%) than globally, suggesting that other mechanisms, mainly biallelic deletion at the *CDKN2A* locus and other epigenetic changes, may drive disease persistence. Our data also support the finding that previously noted co-occurring *CDKN2A* deletions and 9p LOH exacerbate disease progression [34]. The demographic profile of this cluster, with a predominance of male patients (76%), aligns with the findings that highlighted a higher prevalence of T-ALL among males [36]. This cluster underscores the importance of intensified treatment strategies to address the dual challenges of genetic risk factors and clinical aggressiveness.

#### 4.2.3. Profiles of Patients—Cluster 3

The third cluster, termed the Genetically Stable Subgroup with Better Treatment Response, stands out for its lack of biallelic *CDKN2A* deletions and a 100% prevalence of normal *CDKN2A* profiles, compared to the global prevalence of 14%. This genetic stability correlates with relatively favorable clinical outcomes. By day 33, 50% of patients achieved negative MRD/PCR results, significantly surpassing the global rate of 21%. Additionally, 64% of patients in this cluster lack LOH at 9p, compared to the global prevalence of 24%. However, MRD levels ≥10% are still observed in 31% of patients at day 15, reflecting the complexity of treatment responses even in genetically stable subgroups. These findings are consistent with observations by Gonzalez-Gil et al., who proposed that intact *CDKN2A* loci bear a protective effect [37].

In the T-ALL group, in Cluster 1, which is characterized by favorable outcomes, LOH is observed at the *CDKN2A* locus in all cases. However, the absence of LOH at chromosome 9p, a distinguishing feature of this cluster, serves to mitigate the destabilizing effects that are typically associated with chromosomal losses. This distinctive combination enables patients of Cluster 1 to experience rapid disease resolution, accompanied by remarkably low MRD levels and consistently favorable prednisone responses. The interplay of LOH at specific loci, such as *CDKN2A*, without broader chromosomal instability may elucidate the superior therapeutic outcomes observed in this group. In contrast, Cluster 2 in T-ALL represents a more challenging subgroup, characterized by an interplay of genetic alterations, including a high frequency of biallelic *CDKN2A* deletions, but minimal LOH at the *CDKN2A* locus or 9p. Finally, T-ALL Cluster 3 presents a genetic and clinical profile characterized by the absence of LOH at both *CDKN2A* and 9p, which reflects a relatively stable chromosomal landscape. Nevertheless, despite this genetic stability, patients in Cluster 3 experience significant clinical challenges, including elevated levels of MRD and suboptimal early treatment responses. This discrepancy between genetic stability and clinical outcomes highlights the complex nature of T-ALL.

Results of the study confirm that B-ALL is heterogeneous, while T-ALL is homogeneous in terms of LOH. What is new in our study and not seen in the literature is the fact that the LOH was less frequent or absent in chromosomes affected by trisomy, and more often in chromosomes not typically with an additional copy number.

This study has several limitations. First, it is a retrospective analysis, which may limit the strength of causal inferences. Second, the sample size, particularly in the T-ALL subgroup, is relatively small, which restricts the statistical power of subgroup comparisons and may affect the detection of less frequent LOH patterns. Moreover, technical constraints of SNP-array resolution and interpretation algorithms could influence the accuracy of LOH identification, especially in complex karyotypes. To address these limitations, future studies should consider prospective designs that integrate LOH analysis with other molecular and clinical risk factors. Incorporating whole-genome or whole-exome sequencing could further improve the resolution and biological interpretation of LOH events in pediatric ALL.

## 5. Conclusions

This study underscores the critical role of LOH in shaping the genetic and clinical landscape of childhood ALL, with LOH at chromosome 9p emerging as a hallmark of high-risk subgroups and a promising prognostic marker and therapeutic target. A key finding of this study is the distinct pattern of LOH in patients with and without hyperdiploidy. In hyperdiploid patients, LOH predominantly involves entire chromosomes, either singly or in groups, whereas patients without hyperdiploidy display primarily segmental LOH affecting specific chromosomal regions. Segmental LOH of chromosome 9 forms a unique subgroup, further highlighting the complexity of these alterations. Moreover, LOH patterns differ significantly between B-ALL and T-ALL. In B-ALL, LOH affects entire chromosomes more frequently and is associated with a higher number of deletions and duplications, while in T-ALL, LOH is most common on the short arm of chromosome 9, with whole-chromosome LOH being exceedingly rare. Using MCA and HCPC, we identified distinct LOH profiles that correlate with MRD, emphasizing the necessity of integrating LOH analysis into risk stratification and treatment planning. These findings provide novel insights into the pathogenesis of childhood ALL and warrant further studies in larger cohorts to explore the full clinical significance of LOH.

This study underscores the relevance of LOH profiling and supports a combined diagnostic approach integrating SNP-array–based analysis with next-generation sequencing (NGS) panels. Such integration would not only preserve the ability to detect CNV, CNN-LOH events but also enrich the genomic landscape by identifying co-existing somatic point mutations, thereby enhancing diagnostic precision and risk stratification—particularly in patients with inconclusive cytogenetic findings or atypical clinical features.

## Figures and Tables

**Table 1:** Demographic and clinical characteristics of pediatric patients with B- and T-ALL with LOH.

Characteristic	B-ALL (*N* = 120)	T-ALL (*N* = 58)
	*n* (%)	*n* (%)
Age, years	5.39 (3.38, 9.23) ^a^	10.33 (4.90, 13.94) ^a^
Gender:		
female	59 (49.17%)	24 (41.38%)
male	61 (50.83%)	34 (58.62%)
WBC [×10^3^/μL]	7.04 (3.69, 25.00) ^a^	117.86 (43.93, 247.06) ^a^
BM, %	91.00 (84.50, 95.38) ^a^	90.00 (80.00, 94.95) ^a^
Response to prednisone:		
good	90 (75.00%)	36 (62.07%)
poor	30 (25.00%)	22 (37.93%)
MRD FMC 15 day:		
<0.1%	40 (33.33%)	11 (18.97%)
0.1–10%	69 (57.50%)	21 (36.21%)
≥10%	11 (9.17%)	26 (44.83%)
MRD/PCR 33 day:		
negative	48 (40.00%)	9 (15.52%)
positive	72 (60.00%)	37 (63.79%)
other (no marker)		12 (20.69%)

Legend: ^a^ Mdn (Q1, Q3). *n* (%) for categorical variables and median (Q1, Q3) for continuous variables; WBC—white blood cell count at diagnosis (×10^3^/μL); BM—percentage of blasts in bone marrow at diagnosis (%); MRD FMC 15 day—minimal residual disease assessed by flow cytometry on day 15; MRD/PCR 33 day—minimal residual disease assessed by polymerase chain reaction on day 33; good/poor prednisone response—determined by blast count in bone marrow on day 8 of treatment; other (no marker)—minimal residual disease not assessed due to lack of polymerase chain reaction target marker. *N*—number of patients included in each group (B-ALL: 120; T-ALL: 58).

**Table 2:** Genetic characteristics of patients with B-ALL.

LOH 9/9p	*N*	*n* (%)
No evidence of LOH	63	52.5%
Patients with 9p LOH extending to 9p13	6	5%
LOH 9	33	27.5%
LOH 9p	3	2.5%
LOH 9q	5	4.2%
Patients with interrupted 9p LOH	10	8.3%
***CDKN2A***	***N***	***n* (%)**
del monoallelic	24	20%
del biallelic	19	15.8%
duplication	14	11.7%
LOH	24	20%
No alterations	39	32.5%

Legend: LOH—loss of heterozygosity; *N*—number of patients included in each subgroup (B-ALL: 120; T-ALL: 58); *n* (%)—number of patients and corresponding percentage within the category.

**Table 3:** Genetic characteristics of patients with T-ALL.

LOH 9/9p	*N*	*n* (%)
No evidence of LOH	14	24%
Patients with 9p LOH extending to 9p13	6	10%
LOH 9	0	0%
LOH 9p	15	26%
Patients with interrupted 9p LOH	23	40%
***CDKN2A***	***N***	***n* (%)**
del monoallelic	12	21%
del biallelic	35	60%
LOH	3	5%
No alterations	8	14%

Legend: LOH—loss of heterozygosity; *N*—number of patients included in each subgroup (B-ALL: 120; T-ALL: 58); *n* (%)—number of patients and corresponding percentage within the category.

## Data Availability

The original contributions presented in the study are included in the article/Supplementary Materials; further inquiries can be directed to the corresponding author.

## Supplementary Materials

The following supporting information can be downloaded at: https://www.mdpi.com/article/10.3390/cancers17152500/s1. Supplementary S1: Methods, Supplementary S2: Results, File S1: Molecular karyotype, File S2: CNA, File S3: CNA in genes.

## Abbreviations

The following abbreviations are used in this manuscript:

ALL	acute lymphoblastic leukemia
B-ALL	B-cell acute lymphoblastic leukemia
T-ALL	T-cell acute lymphoblastic leukemia
LOH	loss of heterozygosity
CNL-LOH	loss of heterozygosity with copy number losses
CNN-LOH	copy number neutral loss of heterozygosity
DNA	deoxyribonucleic acid
FMC MRD	flow cytometry-minimal residual disease
MRD/PCR	minimal residual disease detected by polymerase chain reaction
SNP	single-nucleotide polymorphism
CNV	copy number variant
CNAs	copy numbers of altered regions
ChAS	Chromosome Analysis Suite
CN	copy number
kbp	kilobase pair
Mbp	megabase pair
BAF	biallelic frequency
p	short arm of a chromosome
q	long arm of a chromosome
MRD	minimal residual disease
WBC	white blood cells
NGS	next-generation sequencing
