# Supplementary material for: Loss of Heterozygosity in Pediatric Acute Lymphoblastic Leukemia and Its Prognostic Impact: A Retrospective Study
Source: Cancers (Basel). 2025 Jul 29;17(15):2500. doi: 10.3390/cancers17152500 (PMC12346631; doi:10.3390/cancers17152500)
Supplement: Supplementary file 1 [file cancers-17-02500-s001.zip › Supplementary materials.pdf]

Supplementary Materials

Article

Loss of heterozygosity in pediatric acute lymphoblastic leukemia and its prognostic impact: a retrospective study

Borys Styka [1,*], Gabriela Ręka [1], Aleksandra Ozygała [1], Mariola Janiszewska [2], Magdalena Stelmach [1], Paulina Skowera [1], Zuzanna Urbańska [3] and Monika Lejman [1]

[1] Independent Laboratory of Genetic Diagnostics, Medical University of Lublin, Lublin, Poland; borys.styka@umlub.pl (B.S.); gabrysia.reka@gmail.com (G.R.); aleksandra.ozygala@uszd.lublin.pl (A.O.); magdalena.stelmach@umlub.pl (M.S.); paulina.skowera@umlub.pl (P.S.); monika.lejman@umlub.pl (M.L.)
[2] Department of Medical Informatics and Statistics with e-Health Lab, Medical University of Lublin, Lublin, Poland; mariola.janiszewska@umlub.pl (M.J.)
[3] Department of Genetic Predisposition to Cancer, Medical University of Lodz, Lodz, Poland; zuzanna.urbanska@umed.lodz.pl (Z.U.)

* borys.styka@umlub.pl, +48 605-577-272

Supplementary S1. METHODS

Supplementary S1.1 Laboratory methods

Bone marrow samples were collected in anticoagulant (EDTA)-containing tubes. Genomic deoxyribonucleic acid (DNA) was isolated using the QIAamp DNA Blood Mini Kit (Qiagen, Hilden, Germany) and stored at -20°C until single nucleotide polymorphism (SNP) array experiments were conducted. The concentration and quality of DNA isolates were assessed using spectrophotometry (NanoDrop One; Thermo Fisher Scientific, Waltham, MA, USA). Microarray analyses were performed using a CytoScan HD array, which included 750,000 SNP markers and 1,900,000 non-polymorphic copy number variant (CNV) markers (Applied Biosystems, part of Thermo Fisher Scientific). All laboratory procedures adhered to the manufacturer's protocols. For the assessment of CNV, a previously standardized microarray method in our laboratory, by the AIEOP-BFM array screening strategy recommendations, was employed. A total of 250 ng of genomic DNA was analyzed following the manufacturer's protocols. Data were generated using a GeneChip Scanner 3000 7G (Thermo Fisher Scientific) and analyzed using Chromosome Analysis Suite v 4.3 (ChAS; Thermo Fisher Scientific). Copy numbers of altered regions (CNAs) and LOH were calculated and normalized to a reference model (Thermo Fisher Scientific) with baseline reference intensities, NA 33 (hg19/CRCh37). Copy number (CN) states and breakpoints were determined using a hidden Markov model software package. A threshold level of log2 ratio ≥0.3 and ≤-0.5 was used to categorize altered chromosomal regions as CNV gains and losses, respectively. The identification of normal diploid markers in the neoplasm samples played a crucial role in the algorithm, especially in cases with significant sample-induced aberrations. Additionally, unaltered diploid markers were utilized for signal calibration, resulting in a log2 ratio of 0 (e.g., copy number 2). The algorithm also adjusted the log2 ratio when identified unaltered diploid markers corresponded to CN=4, reporting a chromosomal ploidy of 4. Loss of heterozygosity was determined based on SNP probe analysis, utilizing allelic difference and biallelic frequency (BAF).

Supplementary S1.2 Statistical methods

In the conducted study, a significance level of $\alpha = 0.05$ was established, allowing for a 5% risk of Type I error.

Descriptive statistics were employed to summarize the data characteristics. For continuous variables, the median (Mdn) was utilized as a measure of central tendency due to its robustness against outliers, along with the first (Q1) and third (Q3) quartiles, which delineate the interquartile range (IQR) of the sample. Categorical variables were reported as frequency (n) and percentage, providing insight into their distribution within the cohort.

The associations between variables were conducted by the multiple correspondence analysis (MCS) [1]. Association between individual parameters and specified dimensions are estimated by the factor analysis using the one-way ANOVA. The effect size of the association was measured by the determination coefficient R2 and the p-values.

To identify distinct clinical profiles of patients, the Hierarchical Clustering on Principal Components method (HCPC) for categorical data was employed, with MCA utilized as a preprocessing step.

The estimation of the number of clusters involves constructing a hierarchical clustering tree from the dataset. Within-cluster inertia, defined as the sum of squared distances between points and their cluster centroids, is calculated for various partitions. The optimal number of clusters n is determined by evaluating the relative loss of inertia i, represented by the ratio:

$$\frac{i(clustersn + 1)}{i(clustersn)}$$

with higher values indicating better clustering quality. A higher relative loss of inertia indicates a more significant improvement in clustering quality, suggesting a preferable partition. The absolute loss of inertia, calculated as i (cluster n)−i (cluster n+1) assists in assessing the trade-offs between the number of clusters and the associated inertia, providing further insight into the clustering structure.

A dimension in MCA represents a principal component derived from the transformation of categorical variables into a lower-dimensional space, capturing the underlying structure of associations among variables. In the T-cell leukemia study, MCA transformed 11 categorical variables (e.g., gender, prednisone response, del *CDKN2A*, LOH 9p) into an 18-dimensional framework, while in the B-cell study, 9 variables (e.g., LOH 9/9p, hyperdiploidy, del *CDKN2A*) were mapped into a 23-dimensional framework, with the variance fully explained in each case. Each dimension is a linear combination of the original variables, ordered by the amount of variance explained, as visualized in scree plots showing the explained variance for the first 10 dimensions. For instance, Dimension 1 in the T-cell study was strongly associated with genetic alterations like del *CDKN2A* ($R^2 = 0.56$, $p<0.001$) and LOH 9p ($R^2 = 0.50$, $p<0.001$), reflecting their significant contribution to the data's structure.

Similarly, in the B-cell study, Dimension 1 was driven by LOH segments (R^2 = 0.63, p<0.001) and hyperdiploidy (R^2 = 0.59, p<0.001). Dimensions thus serve as axes that summarize the variability and relationships among categorical variables, facilitating the identification of patterns without assuming linear dependencies.
A cluster, in contrast, refers to a group of patients identified through HCPC, where individuals are grouped based on their similarity across the principal components derived from MCA. In both studies, HCPC applied to MCA's principal components yielded three clusters: 12, 36, and 10 patients in the T-cell cohort, and 30, 54, and 36 patients in the B-cell cohort. These clusters explained 26.3% of the total variance in the T-cell study (14.7% in Dimension 1, 11.6% in Dimension 2) and 22.5% in the B-cell study (12.1% in Dimension 1, 10.4% in Dimension 2). Clusters were validated using within-cluster inertia, the sum of squared distances between patients and their cluster centroids, with the optimal three-cluster solution determined by maximizing the relative loss of inertia. For example, T-cell Cluster 1 was characterized by low MRD FMC 15 day (<0.1%, v-test = 5.68, p<0.001), while B-cell Cluster 1 was defined by LOH 9/9p: LOH 9 (v-test = 9.73, p<0.001). Clusters thus represent homogeneous subgroups of patients with shared clinical and genetic profiles, as visualized in two-dimensional plots.

The association between the studied variables and the identified clusters was assessed using the Pearson chi-square test. Additionally, the difference in prevalence of specific characteristics among patients within the designated clusters compared to the overall study population was evaluated using the v-test.

Supplementary S1.3 Characteristics of the applied statistical tool

Analyses were conducted using the R Statistical language (version 4.3.3) [2] on Windows 11 Pro 64 bit (build 22631), using the packages factoextra (version 1.0.7) [3], FactoMineR (version 2.11) [4], report (version 0.5.8) [5], gtsummary (version 1.7.2) [6], ggplot2 (version 3.5.0) [7], readxl (version 1.4.3) [8] and dplyr (version 1.1.4) [9].

1. Husson, F.; Le, S.; Pages, J. Exploratory Multivariate Analysis by Example Using R, Chapman and Hall. **2010.**

2. R Core Team. R: A Language and Environment for Statistical Computing. R Foundation for Statistical Computing, Vienna, Austria. **2024**, https://www.R-project.org/.

3. Kassambara, A.; Mundt, F. factoextra: Extract and Visualize the Results of Multivariate Data Analyses. R package version 1.0.7. **2020,** https://CRAN.R-project.org/package=factoextra.

4. Lê, S.; Josse, J.; Husson, F. FactoMineR: A Package for Multivariate Analysis, *Journal of Statistical Software.* **2020**, 1-18. doi:10.18637/jss.v025.i01.

5. Makowski, D.; Lüdecke, D.; Patil, I.; Thériault, R.; Ben-Shachar, M.; Wiernik, B. Automated Results Reporting as a Practical Tool to Improve Reproducibility and Methodological Best Practices Adoption, *CRAN*. **2023**, https://easystats.github.io/report/.

6. Sjoberg, D.; Whiting, K.; Curry, M.; Lavery, J.; Larmarange, J. Reproducible Summary Tables with the gtsummary Package, *The R Journal.* **2021**, 570-580. doi:10.32614/RJ-2021-053

7. Wickham, H. ggplot2: Elegant Graphics for Data Analysis. *Springer-Verlag New York*. **2016**, https://ggplot2.tidyverse.org.

8. Wickham, H.; Bryan, J. Read Excel Files. R package version 1.4.3, **2023**, https://CRAN.R-project.org/package=readxl.

9. Wickham, H.; François, R.; Henry, L.; Müller, K.; Vaughan, D. dplyr: A Grammar of Data Manipulation. R package version 1.1.4, **2023**, https://CRAN.R-project.org/package=dplyr.

Supplementary S2. RESULTS

Supplementary S2.1 Tables

Supplementary Table S1. The list of genes included in cytoregions in the microarray analysis.

No.	Gene name	Chromosome localization
**1**	*DVL1*	chr1 (1270658-1284492)
**2**	*MIB2*	chr1 (1550795-1565990)
**3**	*PRDM16*	chr1 (2985741-3355185)
**4**	*RPL22*	chr1 (6245079-6259679)
**5**	*CAMTA1*	chr1 (6845384-6932107)
**6**	*ERRFI1*	chr1 (8071779-8086393)
**7**	*ENO1*	chr1 (8921059-8931635)
**8**	*PIK3CD*	chr1 (9711790-9789172)
**9**	*MTOR*	chr1 (11166588-11322608)
**10**	*MTHFR*	chr1 (11845787-11866160)
**11**	*PRDM2*	chr1 (14075876-14114574)
**12**	*CASP9*	chr1 (15818769-15850940)
**13**	*SDHB*	chr1 (17345225-17380665)
**14**	*PLA2G2A*	chr1 (20301924-20306932)
**15**	*CDA*	chr1 (20915444-20945400)
**16**	*RAP1GAP*	chr1 (21922708-21995856)
**17**	*HSPG2*	chr1 (22148737-22263750)
**18**	*CDC42*	chr1 (22379120-22419436)
**19**	*HNRNPR*	chr1 (23636276-23670853)
**20**	*MDS2*	chr1 (23953823-23967056)
**21**	*IL22RA1*	chr1 (24446261-24469775)
**22**	*CLIC4*	chr1 (25071760-25170815)
**23**	*RPS6KA1*	chr1 (26872343-26901520)
**24**	*PPP1R8*	chr1 (28157325-28178183)
**25**	*RPA2*	chr1 (28218049-28241236)
**26**	*COL16A1*	chr1 (32117848-32169768)
**27**	*LCK*	chr1 (32739711-32751768)
**28**	*SFPQ*	chr1 (35649200-35658743)
**29**	*CSF3R*	chr1 (36931643-36948915)
**30**	*RSPO1*	chr1 (38076951-38100595)
**31**	*CITED4*	chr1 (41326728-41328018)
**32**	*YBX1*	chr1 (43148066-43168020)
**33**	*MPL*	chr1 (43803474-43820135)
**34**	*CDC20*	chr1 (43824626-43828873)
**35**	*PTCH2*	chr1 (45285516-45308616)
**36**	*MUTYH*	chr1(45794914-45806142)
**37**	*PRDX1*	chr1 (45976707-45988562)
**38**	*TSPAN1*	chr1 (46640749-46651634)
**39**	*PDZK1IP1*	chr1 (47649261-47655771)
**40**	*TAL1*	chr1 (47681961-47697387)
**41**	*STIL*	chr1 (47715810-47779819)
**42**	*CDKN2C*	chr1 (51434366-51440306)
**43**	*EPS15*	chr1 (51819934-51887793)
**44**	*JUN*	chr1 (59246463-59249785)

**45**	*JAK1*	chr1 (65298905-65432187)
**46**	*MIER1*	chr1 (67390578-67454302)
**47**	*DIRAS3*	chr1 (68511645-68516460)
**48**	*LPHN2*	chr1 (82266082-82458107)
**49**	*BCL10*	chr1 (85731460-85742587)
**50**	*SEP15*	chr1 (87328128-87380107)
**51**	*GBP1*	chr1 (89517987-89531043)
**52**	*TGFBR3*	chr1 (92145900-92351836)
**53**	*GLMN*	chr1 (92711955-92764566)
**54**	*GFI1*	chr1 (92940318-92949356)
**55**	*RPL5*	chr1 (93297593-93307481)
**56**	*VAV3*	chr1 (108113782-108231126)
**57**	*RBM15*	chr1 (110881944-110889303)
**58**	*CD53*	chr1 (111415722-111442558)
**59**	*WDR77*	chr1 (111982512-111991830)
**60**	*RAP1A*	chr1 (112162405-112256101)
**61**	*RHOC*	chr1 (113243749-113250025)
**62**	*SLC16A1*	chr1 (113454470-113498685)
**63**	*BCL2L15*	chr1 (114419436-114430169)
**64**	*HIPK1*	chr1 (114471995-114514698)
**65**	*NRAS*	chr1 (115247085-115259515)
**66**	*VTCN1*	chr1 (117686209-117753549)
**67**	*FAM46C*	chr1 (118148603-118171011)
**68**	*REG4*	chr1 (120336641-120354203)
**69**	*NOTCH2*	chr1 (120477736-120612317)
**70**	*PDE4DIP*	chr1 (144951760-144995033)
**71**	*PIAS3*	chr1 (145575988-145586546)
**72**	*BCL9*	chr1 (147013270-147098020)
**73**	*ECM1*	chr1 (150480487-150486265)
**74**	*ARNT*	chr1 (150782180-150849244)
**75**	*MLLT11*	chr1 (151032150-151040973)
**76**	*S100A10*	chr1 (151955386-151966714)
**77**	*S100A9*	chr1 (153330330-153333503)
**78**	*S100A8*	chr1 (153362508-153363664)
**79**	*S100A7*	chr1 (153430220-153433137)
**80**	*S100A4*	chr1 (153516098-153518282)
**81**	*S100A13*	chr1 (153591276-153606568)
**82**	*S100A1*	chr1 (153600873-153604513)
**83**	*CRTC2*	chr1 (153920148-153931132)
**84**	*RPS27*	chr1 (153963239-153964631)
**85**	*TPM3*	chr1 (154127780-154155725)
**86**	*SHC1*	chr1 (154934774-154943223)
**87**	*CKS1B*	chr1 (154947118-154951725)
**88**	*ARHGEF2*	chr1 (155916630-155948336)
**89**	*MEF2D*	chr1 (156433519-156470529)
**90**	*PRCC*	chr1 (156737274-156770609)
**91**	*IGSF8*	chr1 (160061130-160068618)
**92**	*USF1*	chr1 (161009041-161015757)
**93**	*PVRL4*	chr1 (161040781-161059385)
**94**	*SDHC*	chr1 (161284166-161334535)
**95**	*FCGR2B*	chr1 (161632904-161648444)
**96**	*PBX1*	chr1 (164528596-164821060)
**97**	*GPA33*	chr1 (167022082-167059868)
**98**	*CREG1*	chr1 (167510250-167523056)
**99**	*RCSD1*	chr1 (167599473-167675486)

**100**	*SELE*	chr1 (169691781-169703220)
**101**	*PRRX1*	chr1 (170633312-170708541)
**102**	*TNFSF18*	chr1 (173010360-173020103)
**103**	*GAS5*	chr1 (173833039-173837125)
**104**	*TNN*	chr1 (175036994-175117202)
**105**	*ABL2*	chr1 (179068461-179198819)
**106**	*DHX9*	chr1 (182808439-182857117)
**107**	*TPR*	chr1 (186280785-186344457)
**108**	*PTGS2*	chr1 (186640944-186649559)
**109**	*PLA2G4A*	chr1 (186798032-186958113)
**110**	*RGS2*	chr1 (192778169-192781407)
**111**	*TROVE2*	chr1 (193028551-193055115)
**112**	*GLRX2*	chr1 (193065600-193075244)
**113**	*CDC73*	chr1 (193091088-193223942)
**114**	*B3GALT2*	chr1 (193147859-193155743)
**115**	*ASPM*	chr1 (197053257-197115824)
**116**	*PTPRC*	chr1 (198608097-198664300)
**117**	*KIF14*	chr1 (200520625-200589862)
**118**	*PTPN7*	chr1 (202116141-202130716)
**119**	*ADIPOR1*	chr1 (202909961-202927700)
**120**	*MAPKAPK2*	chr1 (206858289-206907626)
**121**	*KCNH1*	chr1 (210851657-211307457)
**122**	*ATF3*	chr1 (212738697-212794116)
**123**	*SMYD2*	chr1 (214454565-214510477)
**124**	*CENPF*	chr1 (214776532-214837914)
**125**	*ESRRG*	chr1 (216676596-217311097)
**126**	*DUSP10*	chr1 (221874764-221915516)
**127**	*TP53BP2*	chr1 (223967595-224033674)
**128**	*ENAH*	chr1 (225674534-225840845)
**129**	*MIXL1*	chr1 (226411383-226413513)
**130**	*PSEN2*	chr1 (227058273-227083804)
**131**	*EGLN1*	chr1 (231499497-231560790)
**132**	*FH*	chr1 (241660857-241683085)
**133**	*AKT3*	chr1 (243663021-244006886)
**134**	*SMYD3*	chr1 (245912642-246670644)
**135**	*SOX11*	chr2 (5832799-5841517)
**136**	*ADAM17*	chr2 (9629411-9695917)
**137**	*E2F6*	chr2 (11584501-11606297)
**138**	*DDX1*	chr2 (15731770-15771225)
**139**	*MYCN*	chr2 (16080683-16087129)
**140**	*SDC1*	chr2 (20400558-20425194)
**141**	*RHOB*	chr2 (20646835-20649201)
**142**	*DNMT3A*	chr2 (25455829-25564784)
**143**	*BRE*	chr2 (28113557-28561767)
**144**	*ALK*	chr2 (29415640-30144477)
**145**	*NLRC4*	chr2 (32449518-32489949)
**146**	*BIRC6*	chr2 (32582096-32843965)
**147**	*STRN*	chr2 (37064841-37193615)
**148**	*EML4*	chr2 (42396490-42559688)
**149**	*MTA3*	chr2 (42795671-42936353)
**150**	*THADA*	chr2 (43457974-43823113)
**151**	*EPCAM*	chr2 (47596287-47614167)
**152**	*MSH2*	chr2 (47630205-47710367)
**153**	*MSH6*	chr2 (48010221-48034092)
**154**	*FBXO11*	chr2 (48034058-48115858)

**155**	*LHCGR*	chr2 (48913913-48982880)
**156**	*RTN4*	chr2 (55199327-55237470)
**157**	*BCL11A*	chr2 (60678301-60780633)
**158**	*REL*	chr2 (61108752-61150178)
**159**	*XPO1*	chr2 (61705068-61765418)
**160**	*LOXL3*	chr2 (74759946-74781062)
**161**	*TMSB10*	chr2 (85132763-85133799)
**162**	*KCMF1*	chr2 (85198231-85286595)
**163**	*CAPG*	chr2 (85621871-85637676)
**164**	*IGK*	chr2 (89156674-90274235)
**165**	*MAL*	chr2 (95691479-95719735)
**166**	*AFF3*	chr2 (100163715-100759037)
**167**	*TGFBRAP1*	chr2 (105883540-105946148)
**168**	*FHL2*	chr2 (105977283-106015575)
**169**	*RANBP2*	chr2 (109335936-109402267)
**170**	*BUB1*	chr2 (111395409-111435684)
**171**	*BCL2L11*	chr2 (111878491-111926022)
**172**	*MERTK*	chr2 (112656191-112786945)
**173**	*TTL*	chr2 (113239742-113290222)
**174**	*IL1B*	chr2 (113587337-113594356)
**175**	*PAX8*	chr2 (113973574-114036498)
**176**	*BIN1*	chr2 (127805608-127864864)
**177**	*ERCC3*	chr2 (128014866-128051752)
**178**	*ZEB2*	chr2 (145141941-145277958)
**179**	*ACVR2A*	chr2 (148602570-148688393)
**180**	*ATF2*	chr2 (175939006-176032897)
**181**	*HOXD13*	chr2 (176957531-176960666)
**182**	*HOXD11*	chr2 (176972083-176974316)
**183**	*MIR10B*	chr2 (177015031-177015140)
**184**	*PDE11A*	chr2 (178487977-178753466)
**185**	*DIRC1*	chr2 (189598465-189654831)
**186**	*PMS1*	chr2 (190648811-190742355)
**187**	*SF3B1*	chr2 (198283520-198299771)
**188**	*HSPD1*	chr2 (198351308-198364640)
**189**	*CFLAR*	chr2 (201983269-202037411)
**190**	*ADAM23*	chr2 (207308368-207482679)
**191**	*MAP2*	chr2 (210288771-210598834)
**192**	*IKZF2*	chr2 (213864410-214016333)
**193**	*BARD1*	chr2 (215593275-215674428)
**194**	*ATIC*	chr2 (216176679-216214496)
**195**	*DIRC3*	chr2 (218148746-218621316)
**196**	*AAMP*	chr2 (219128852-219134893)
**197**	*PAX3*	chr2 (223158349-223163715)
**198**	*PTMA*	chr2 (232573235-232578250)
**199**	*INPP5D*	chr2 (233924676-234116549)
**200**	*CXCR7*	chr2 (237478380-237490994)
**201**	*SEPT2*	chr2 (242254723-242293441)
**202**	*BOK*	chr2 (242498192-242513553)
**203**	*FEV*	chr2 (219850379-219845808)
**204**	*GIGYF2*	chr2 (233562014-233725289)
**205**	*FANCD2*	chr3 (10068113-10143614)
**206**	*VHL*	chr3 (10183319-10195354)
**207**	*GHRL*	chr3 (10327434-10332419)
**208**	*PPARG*	chr3 (12329349-12475855)
**209**	*RAF1*	chr3 (12625100-12705700)

**210**	*XPC*	chr3 (14186648-14220172)
**211**	*SATB1*	chr3 (18389133-18480265)
**212**	*MLH1*	chr3 (37034840-37092337)
**213**	*PLCD1*	chr3 (38048987-38066278)
**214**	*CTNNB1*	chr3 (41240942-41281939)
**215**	*LIMD1*	chr3 (45636323-45722755)
**216**	*CCR9*	chr3 (45927996-45944667)
**217**	*CCR1*	chr3 (46243200-46249832)
**218**	*CCR2*	chr3 (46395235-46402413)
**219**	*SMARCC1*	chr3 (47627377-47823405)
**220**	*MAP4*	chr3 (47892179-48130769)
**221**	*CDC25A*	chr3 (48198668-48229801)
**222**	*PLXNB1*	chr3 (48445261-48471460)
**223**	*NCKIPSD*	chr3 (48711278-48723334)
**224**	*RHOA*	chr3 (49396579-49449526)
**225**	*TCTA*	chr3 (49449639-49453909)
**226**	*MST1R*	chr3 (49924436-49941306)
**227**	*RBM5*	chr3 (50126352-50156392)
**228**	*SEMA3F*	chr3 (50192848-50226508)
**229**	*SEMA3B*	chr3 (50305040-50314572)
**230**	*HYAL1*	chr3 (50337321-50341004)
**231**	*HYAL2*	chr3 (50355220-50360281)
**232**	*RASSF1*	chr3 (50367216-50378367)
**233**	*HEMK1*	chr3 (50606908-50622421)
**234**	*CISH*	chr3 (50643884-50649262)
**235**	*BAP1*	chr3 (52435025-52444009)
**236**	*PRKCD*	chr3 (53195223-53226733)
**237**	*WNT5A*	chr3 (55499743-55521331)
**238**	*FAM107A*	chr3 (58549845-58563491)
**239**	*FHIT*	chr3 (59735035-61237133)
**240**	*PTPRG*	chr3 (61547243-62280573)
**241**	*LRIG1*	chr3 (66429221-66550845)
**242**	*FOXP1*	chr3 (71247033-71633140)
**243**	*ROBO1*	chr3 (78646388-79068609)
**244**	*EPHA3*	chr3 (89156674-89531284)
**245**	*MINA*	chr3 (97660661-97691224)
**246**	*TFG*	chr3 (100428134-100467811)
**247**	*CBLB*	chr3 (105377108-105587887)
**248**	*KIAA1524*	chr3 (108268718-108308491)
**249**	*CD200*	chr3 (112051915-112081658)
**250**	*BTLA*	chr3 (112182812-112218408)
**251**	*GSK3B*	chr3 (119540802-119813264)
**252**	*CSTA*	chr3 (122044011-122060815)
**253**	*HSPBAP1*	chr3 (122458846-122512650)
**254**	*DIRC2*	chr3 (122513901-122599986)
**255**	*MUC13*	chr3 (124624289-124653595)
**256**	*RUVBL1*	chr3 (127799800-127842671)
**257**	*GATA2*	chr3 (128198264-128207373)
**258**	*RPN1*	chr3 (128338812-128369719)
**259**	*MBD4*	chr3 (129149793-129158852)
**260**	*FAIM*	chr3 (138327542-138352213)
**261**	*RNF7*	chr3 (141457051-141465645)
**262**	*ATR*	chr3 (142168077-142297668)
**263**	*HLTF*	chr3 (148747904-148804341)
**264**	*SIAH2*	chr3 (150458910-150481263)

**265**	*RAP2B*	chr3 (152880029-152886263)
**266**	*MME*	chr3 (154797436-154901518)
**267**	*GMPS*	chr3 (155588324-155655520)
**268**	*MLF1*	chr3 (158288952-158324249)
**269**	*RARRES1*	chr3 (158422440-158450275)
**270**	*MECOM*	chr3 (168801286-168864093)
**271**	*PLD1*	chr3 (171318618-171528273)
**272**	*TNFSF10*	chr3 (172223298-172241297)
**273**	*TBL1XR1*	chr3 (176738541-176915048)
**274**	*PIK3CA*	chr3 (178866311-178952497)
**275**	*SOX2*	chr3 (181429712-181432223)
**276**	*EIF4A2*	chr3 (186501361-186507685)
**277**	*BCL6*	chr3 (187439164-187454285)
**278**	*LPP*	chr3 (187943193-188608460)
**279**	*MUC4*	chr3 (195473638-195538844)
**280**	*TFRC*	chr3 (195776155-195809032)
**281**	*PAK2*	chr3 (196466728-196559518)
**282**	*DLG1*	chr3 (196769431-197025447)
**283**	*ARL8B*	chr3 (5163929-5222601)
**284**	*EDEM1*	chr3 (5229358-5261650)
**285**	*MECOM*	chr3 (169381563-168801286)
**286**	*TACC3*	chr4 (1723266-1746897)
**287**	*FGFR3*	chr4 (1795038-1810599)
**288**	*WHSC1*	chr4 (1873122-1983934)
**289**	*S100P*	chr4 (6695566-6698897)
**290**	*RHOH*	chr4 (40198527-40246281)
**291**	*FRYL*	chr4 (48499380-48782316)
**292**	*RASL11B*	chr4 (53728495-53733002)
**293**	*CHIC2*	chr4 (54875957-54930815)
**294**	*PDGFRA*	chr4 (55095264-55164412)
**295**	*KIT*	chr4 (55524095-55606881)
**296**	*REST*	chr4 (57775079-57802010)
**297**	*RASSF6*	chr4 (74438862-74486134)
**298**	*AREG*	chr4 (75310853-75320726)
**299**	*RCHY1*	chr4 (76404247-76439640)
**300**	*HNRNPD*	chr4 (83274467-83295149)
**301**	*MAPK10*	chr4 (86936276-87374283)
**302**	*PTPN13*	chr4 (87515468-87736328)
**303**	*AFF1*	chr4 (87856153-88062206)
**304**	*SPP1*	chr4 (88896802-88904563)
**305**	*RAP1GDS1*	chr4 (99182526-99365012)
**306**	*EIF4E*	chr4 (99799607-99850243)
**307**	*NFKB1*	chr4 (103422486-103538459)
**308**	*TET2*	chr4 (106067841-106200960)
**309**	*LEF1*	chr4 (108968700-109090112)
**310**	*SYNPO2*	chr4 (119809996-119959669)
**311**	*MAD2L1*	chr4 (120980579-120988013)
**312**	*FGF2*	chr4 (123747863-123819390)
**313**	*NUDT6*	chr4 (123813799-123843759)
**314**	*NR3C2*	chr4 (148999914-149363672)
**315**	*FBXW7*	chr4 (153242409-153303664)
**316**	*NPY1R*	chr4 (164245117-164253947)
**317**	*ING2*	chr4 (184426220-184432249)
**318**	*SORBS2*	chr4 (186506598-186733410)
**319**	*FAT1*	chr4 (187508937-187644987)

**320**	*FIP1L1*	chr4 (54243819-54326103)
**321**	*MAML3*	chr4 (141075233-140637545)
**322**	*DUX4*	chr4 (190992289-190993669)
**323**	*PDCD6*	chr5 (271736-315089)
**324**	*TRIO*	chr5 (14143829-14509458)
**325**	*PRLR*	chr5 (35055802-35230823)
**326**	*IL7R*	chr5 (35856976-35879705)
**327**	*LIFR*	chr5 (38475065-38556748)
**328**	*DAB2*	chr5 (39371780-39425335)
**329**	*SEPP1*	chr5 (42799982-42812024)
**330**	*FST*	chr5 (52776264-52782304)
**331**	*GZMA*	chr5 (54398474-54406080)
**332**	*ERCC8*	chr5 (60169659-60240905)
**333**	*CCNB1*	chr5 (68462913-68474070)
**334**	*ENC1*	chr5 (73923234-73937249)
**335**	*IQGAP2*	chr5 (75843233-76003957)
**336**	*MSH3*	chr5 (79950294-80172634)
**337**	*SSBP2*	chr5 (80713178-81047072)
**338**	*VCAN*	chr5 (82767493-82878122)
**339**	*TSLP*	chr5 (110405777-110413722)
**340**	*APC*	chr5 (112073556-112181936)
**341**	*LOX*	chr5 (121398890-121412918)
**342**	*SNX2*	chr5 (122110690-122170234)
**343**	*FNIP1*	chr5 (130977407-131132756)
**344**	*ACSL6*	chr5 (131285667-131347355)
**345**	*IL3*	chr5 (131396347-131398896)
**346**	*IRF1*	chr5 (131817301-131826465)
**347**	*AFF4*	chr5 (132211070-132299354)
**348**	*TGFBI*	chr5 (135364584-135399507)
**349**	*EGR1*	chr5 (137801181-137805004)
**350**	*HDAC3*	chr5 (141000443-141016423)
**351**	*ARHGAP26*	chr5 (142150291-142608572)
**352**	*NR3C1*	chr5 (142657495-142783254)
**353**	*SPINK7*	chr5 (147691990-147695481)
**354**	*CSNK1A1*	chr5 (148874845-148931007)
**355**	*CSF1R*	chr5 (149432853-149492935)
**356**	*PDGFRB*	chr5 (149493401-149535422)
**357**	*TNIP1*	chr5 (150409503-150467221)
**358**	*ITK*	chr5 (156607907-156682109)
**359**	*EBF1*	chr5 (158122922-158526788)
**360**	*PTTG1*	chr5 (159848865-159855746)
**361**	*RANBP17*	chr5 (170288885-170727019)
**362**	*TLX3*	chr5 (170736287-170739138)
**363**	*NPM1*	chr5 (170814707-170833731)
**364**	*NKX2-5*	chr5 (172659107-172662315)
**365**	*NSD1*	chr5 (176560832-176727214)
**366**	*MAPK9*	chr5 (179673028-179719071)
**367**	*GNB2L1*	chr5 (180663928-180670906)
**368**	*MEF2C*	chr5 (88119744-88014057)
**369**	*IRF4*	chr6 (391738-411443)
**370**	*CAGE1*	chr6 (7326887-7389942)
**371**	*TFAP2A*	chr6 (10396916-10412607)
**372**	*DEK*	chr6 (18224399-18264799)
**373**	*ID4*	chr6 (19837617-19840915)
**374**	*E2F3*	chr6 (20404034-20493945)

**375**	*SOX4*	chr6 (21593972-21598849)
**376**	*HFE*	chr6 (26087509-26095469)
**377**	*HIST1H4F*	chr6 (26240653-26241021)
**378**	*HIST1H4G*	chr6 (26246838-26247205)
**379**	*HIST1H3F*	chr6 (26250369-26250835)
**380**	*HIST1H2BH*	chr6 (26251878-26252303)
**381**	*IER3*	chr6 (30710976-30712327)
**382**	*DDR1*	chr6 (30856465-30867933)
**383**	*LTA*	chr6 (31540093-31542098)
**384**	*TNF*	chr6 (31543350-31546112)
**385**	*AGER*	chr6 (32148745-32152099)
**386**	*DAXX*	chr6 (33286335-33290793)
**387**	*BAK1*	chr6 (33540323-33548070)
**388**	*HMGA1*	chr6 (34204577-34214008)
**389**	*PPARD*	chr6 (35310335-35395968)
**390**	*FANCE*	chr6 (35420138-35434881)
**391**	*MAPK14*	chr6 (35995454-36079013)
**392**	*MAPK13*	chr6 (36098262-36107842)
**393**	*CDKN1A*	chr6 (36646456-36655116)
**394**	*PIM1*	chr6 (37137922-37143204)
**395**	*ZFAND3*	chr6 (37787306-38122399)
**396**	*TFEB*	chr6 (41651716-41703997)
**397**	*CCND3*	chr6 (41902670-42016632)
**398**	*POLH*	chr6 (43543878-43588260)
**399**	*NFKBIE*	chr6 (44225902-44233525)
**400**	*RUNX2*	chr6 (45296054-45518819)
**401**	*IL17A*	chr6 (52051185-52055436)
**402**	*SMAP1*	chr6 (71377479-71571716)
**403**	*DDX43*	chr6 (74104285-74127289)
**404**	*EEF1A1*	chr6 (74225473-74230755)
**405**	*CD109*	chr6 (74405508-74538041)
**406**	*CASP8AP2*	chr6 (90539619-90584155)
**407**	*BACH2*	chr6 (90636247-91006627)
**408**	*MAP3K7*	chr6 (91225353-91296907)
**409**	*EPHA7*	chr6 (93949740-94129300)
**410**	*CCNC*	chr6 (99990262-100016690)
**411**	*LIN28B*	chr6 (105404923-105531206)
**412**	*PRDM1*	chr6 (106534195-106557814)
**413**	*FOXO3*	chr6 (108882068-109005971)
**414**	*ARMC2*	chr6 (109169618-109295675)
**415**	*SESN1*	chr6 (109307639-109330758)
**416**	*WISP3*	chr6 (112375278-112390887)
**417**	*HDAC2*	chr6 (114257320-114292359)
**418**	*CEP85L*	chr6 (118781934-119031238)
**419**	*RNF217-AS1*	chr6 (125229391-125284173)
**420**	*CTGF*	chr6 (132269317-132272518)
**421**	*MYB*	chr6 (135502452-135540311)
**422**	*hsa-mir-548a-2*	chr6 (135560297-135560394)
**423**	*AHI1*	chr6 (135605109-135818903)
**424**	*BCLAF1*	chr6 (136578001-136610989)
**425**	*TNFAIP3*	chr6 (138188581-138204449
**426**	*ECT2L*	chr6 (139117247-139225207)
**427**	*CITED2*	chr6 (139693397-139695499)
**428**	*PLAGL1*	chr6 (144261437-144385735)
**429**	*LATS1*	chr6 (149982051-150039392)

**430**	*AKAP12*	chr6 (151561134-151679694)
**431**	*IGF2R*	chr6 (160390131-160527583)
**432**	*RNASET2*	chr6 (167343004-167370077)
**433**	*FGFR1OP*	chr6 (167412804-167455906)
**434**	*MLLT4*	chr6 (168227670-168365793)
**435**	*THBS2*	chr6 (169615875-169654137)
**436**	*JARID2*	chr6 (15249085-15522273)
**437**	*CYP2W1*	chr7 (1022835-1029276)
**438**	*GPER*	chr7 (1126443-1133451)
**439**	*ACTB*	chr7 (5566779-5570232)
**440**	*FSCN1*	chr7 (5632454-5646286)
**441**	*THSD7A*	chr7 (11410061-11871824)
**442**	*TWIST1*	chr7 (19155091-19157295)
**443**	*ABCB5*	chr7 (20655245-20796637)
**444**	*IL6*	chr7 (22766766-22771621)
**445**	*GPNMB*	chr7 (23286316-23314729)
**446**	*NPY*	chr7 (24323807-24331484)
**447**	*HOXA9*	chr7 (27202056-27205149)
**448**	*HOXA11*	chr7 (27220775-27224835)
**449**	*HOXA13*	chr7 (27236498-27239725)
**450**	*JAZF1*	chr7 (27870193-28220437)
**451**	*ANLN*	chr7 (36429432-36493400)
**452**	*SFRP4*	chr7 (37945535-37956525)
**453**	*TRG2*	chr7 (38279625-38407656)
**454**	*POU6F2*	chr7 (39017609-39504390)
**455**	*HUS1*	chr7 (48002885-48019222)
**456**	*IKZF1*	chr7 (50344264-50472798)
**457**	*DDC*	chr7 (50526134-50628768)
**458**	*GRB10*	chr7 (50657760-50800050)
**459**	*AUTS2*	chr7 (69063904-70258054)
**460**	*POM121*	chr7 (72349905-72421979)
**461**	*BCL7B*	chr7 (72950683-72972065)
**462**	*CLDN4*	chr7 (73245193-73247015)
**463**	*ELN*	chr7 (73442118-73484236)
**464**	*LIMK1*	chr7 (73507486-73536855)
**465**	*POM121C*	chr7 (75046059-75115565)
**466**	*HIP1*	chr7 (75162618-75368290)
**467**	*HSPB1*	chr7 (75931875-75933614)
**468**	*HGF*	chr7 (81331444-81399452)
**469**	*DMTF1*	chr7 (86781677-86825648)
**470**	*ABCB1*	chr7 (87133179-87342639)
**471**	*STEAP1*	chr7 (89783689-89794141)
**472**	*STEAP2*	chr7 (89841174-89866992)
**473**	*AKAP9*	chr7 (91570189-91739987)
**474**	*ERVW-1*	chr7 (91935631-91936674)
**475**	*GATAD1*	chr7 (92076764-92088742)
**476**	*PEX1*	chr7 (92116336-92157845)
**477**	*CDK6*	chr7 (92234234-92465941)
**478**	*COL1A2*	chr7 (94023873-94060544)
**479**	*PEG10*	chr7 (94285637-94299006)
**480**	*TAC1*	chr7 (97361271-97369784)
**481**	*ASNS*	chr7 (97481429-97501854)
**482**	*MUC17*	chr7 (100663364-100702140)
**483**	*RELN*	chr7 (103112231-103629963)
**484**	*HBP1*	chr7 (106809460-106842974)

**485**	*NRCAM*	chr7 (107788071-107880614)
**486**	*CAV1*	chr7 (116165063-116201239)
**487**	*MET*	chr7 (116312459-116438440)
**488**	*SPAM1*	chr7 (123565286-123600100)
**489**	*POT1*	chr7 (124462439-124570037)
**490**	*NRF1*	chr7 (129269919-129396922)
**491**	*CREB3L2*	chr7 (137559725-137686846)
**492**	*TRIM24*	chr7 (138145079-138270332)
**493**	*ZC3HAV1*	chr7 (138728265-138794465)
**494**	*TTC26*	chr7 (138818489-138876732)
**495**	*HIPK2*	chr7 (139246316-139477693)
**496**	*BRAF*	chr7 (140433813-140624564)
**497**	*TRB*	chr7 (141999017-142511084)
**498**	*TRB@*	chr7 (142495143-142500202)
**499**	*TRPV6*	chr7 (142568960-142583477)
**500**	*EPHA1*	chr7 (143088205-143105985)
**501**	*SHH*	chr7 (155595558-155604967)
**502**	*MNX1*	chr7 (156797546-156802129)
**503**	*EZH2*	chr7 (148581441-148504463)
**504**	*MCPH1*	chr8 (6264113-6304940)
**505**	*DEFB1*	chr8 (6728097-6735529)
**506**	*TNKS*	chr8 (9413445-9639856)
**507**	*CTSB*	chr8 (11700034-11725646)
**508**	*DLC1*	chr8 (12940872-12973753)
**509**	*MTUS1*	chr8 (17501303-17658426)
**510**	*PCM1*	chr8 (17780365-17887457)
**511**	*NAT1*	chr8 (18067618-18081198)
**512**	*NAT2*	chr8 (18248755-18258723)
**513**	*KIAA1967*	chr8 (22462539-22477983)
**514**	*RHOBTB2*	chr8 (22844930-22877710)
**515**	*LOXL2*	chr8 (23154410-23261722)
**516**	*NKX3-1*	chr8 (23536206-23540450)
**517**	*PTK2B*	chr8 (27168998-27316908)
**518**	*CLU*	chr8 (27454434-27472328)
**519**	*WRN*	chr8 (30890778-31031277)
**520**	*EIF4EBP1*	chr8 (37888020-37917883)
**521**	*ASH2L*	chr8 (37963349-37997228)
**522**	*WHSC1L1*	chr8 (38173934-38239790)
**523**	*FGFR1*	chr8 (38268655-38326352)
**524**	*TACC1*	chr8 (38644722-38710546)
**525**	*ADAM9*	chr8 (38854505-38962779)
**526**	*IDO1*	chr8 (39771328-39786309)
**527**	*IDO2*	chr8 (39792474-39873910)
**528**	*KAT6A*	chr8 (41786996-41909505)
**529**	*SNAI2*	chr8 (49830239-49833999)
**530**	*PLAG1*	chr8 (57073468-57123859)
**531**	*CYP7A1*	chr8 (59402737-59412720)
**532**	*TOX*	chr8 (59717976-60031767)
**533**	*GGH*	chr8 (63927639-63951610)
**534**	*CYP7B1*	chr8 (65508529-65711348)
**535**	*MYBL1*	chr8 (67474410-67525480)
**536**	*NCOA2*	chr8 (71024266-71316020)
**537**	*TPD52*	chr8 (80947105-80993010)
**538**	*PAG1*	chr8 (81880045-82024303)
**539**	*WWP1*	chr8 (87354994-87480178)

**540**	*NBN*	chr8 (90945564-90996899)
**541**	*RUNX1T1*	chr8 (92967194-93115454)
**542**	*TP53INP1*	chr8 (95938200-95961615)
**543**	*COX6C*	chr8 (100890223-100906242)
**544**	*BAALC*	chr8 (104152921-104242533)
**545**	*CTHRC1*	chr8 (104383786-104395217)
**546**	*EBAG9*	chr8 (110552310-110577391)
**547**	*RAD21*	chr8 (117858172-117887105)
**548**	*EXT1*	chr8 (118811602-119124058)
**549**	*TNFRSF11B*	chr8 (119935796-119964383)
**550**	*ENPP2*	chr8 (120569319-120651106)
**551**	*HAS2*	chr8 (122625271-122653630)
**552**	*RNF139*	chr8 (125487008-125500859)
**553**	*MYC*	chr8 (128748314-128753680)
**554**	*NDRG1*	chr8 (134249414-134309547)
**555**	*PTK2*	chr8 (141668481-142011412)
**556**	*PSCA*	chr8 (143761874-143764145)
**557**	*MAFA*	chr8 (144510230-144512602)
**558**	*BOP1*	chr8 (145486056-145515120)
**559**	*RECQL4*	chr8 (145736667-145743210)
**560**	*CEBPD*	chr8 (48650726-48649475)
**561**	*JAK2*	chr9 (4985244-5128183)
**562**	*RLN2*	chr9 (5299868-5304580)
**563**	*KDM4C*	chr9 (6757641-7170920)
**564**	*PTPRD*	chr9 (8314246-8733946)
**565**	*PSIP1*	chr9 (15464064-15511003)
**566**	*SH3GL2*	chr9 (17578953-17797122)
**567**	*MLLT3 (AF9)*	chr9 (20341662-20621986)
**568**	*IFNA1*	chr9 (21440452-21441315)
**569**	*MTAP*	chr9 (21802634-21865969)
**570**	*CDKN2A*	chr9 (21967750-21975132)
**571**	*CDKN2B*	chr9 (22002901-22009312)
**572**	*TEK*	chr9 (27109147-27230172)
**573**	*TOPORS*	chr9 (32540542-32552626)
**574**	*BAG1*	chr9 (33252470-33264759)
**575**	*SIGMAR1*	chr9 (34634718-34637823)
**576**	*IL11RA*	chr9 (34652181-34661898)
**577**	*CCL27*	chr9 (34661879-34662689)
**578**	*FANCG*	chr9 (35073835-35080013)
**579**	*STOML2*	chr9 (35099889-35103154)
**580**	*RMRP*	chr9 (35657748-35658015)
**581**	*PAX5*	chr9 (36833271-37034476)
**582**	*TJP2*	chr9 (71736224-71870124)
**583**	*ANXA1*	chr9 (75766781-75785307)
**584**	*GNAQ*	chr9 (80335191-80646219)
**585**	*HNRNPK*	chr9 (86582998-86595569)
**586**	*MIR7-1*	chr9 (86584663-86584772)
**587**	*NTRK2*	chr9 (87284626-87430621)
**588**	*DAPK1*	chr9 (90112756-90323549)
**589**	*CTSL1*	chr9 (90340974-90346384)
**590**	*CDK20*	chr9 (90581359-90589695)
**591**	*CKS2*	chr9 (91926113-91931618)
**592**	*SEMA4D*	chr9 (91975706-92094611)
**593**	*SYK*	chr9 (93563961-93660842)
**594**	*ROR2*	chr9 (94484878-94712444)

**595**	*WNK2*	chr9 (95947212-96082854)
**596**	*FAM22F*	chr9 (97080477-97090926)
**597**	*FANCC*	chr9 (97861336-98079991)
**598**	*PTCH1*	chr9 (98205264-98270831)
**599**	*XPA*	chr9 (100437191-100459691)
**600**	*FOXE1*	chr9 (100615537-100618997)
**601**	*NR4A3*	chr9 (102584137-102629173)
**602**	*ALDOB*	chr9 (104182842-104198062)
**603**	*TAL2*	chr9 (108424737-108425385)
**604**	*KLF4*	chr9 (110247133-110252047)
**605**	*TXN*	chr9 (113006310-113018778)
**606**	*LPAR1*	chr9 (113636054-113800365)
**607**	*TNFSF15*	chr9 (117546915-117554810)
**608**	*TNC*	chr9 (117781854-117880536)
**609**	*CNTRL*	chr9 (123850573-123939886)
**610**	*DAB2IP*	chr9 (124329399-124547809)
**611**	*HSPA5*	chr9 (127997127-128003666)
**612**	*SET*	chr9 (131445933-131458675)
**613**	*PPP2R4*	chr9 (131873967-131911225)
**614**	*PRRX2*	chr9 (132427920-132484951)
**615**	*FNBP1*	chr9 (132649465-132805473)
**616**	*ABL1*	chr9 (133589267-133763062)
**617**	*NUP214*	chr9 (134000947-134110057)
**618**	*RAPGEF1*	chr9 (134452157-134612925)
**619**	*TSC1*	chr9 (135766735-135820020)
**620**	*GFI1B*	chr9 (135854098-135867084)
**621**	*PAEP*	chr9 (138453604-138458622)
**622**	*NOTCH1*	chr9 (139388895-139440238)
**623**	*MIR126*	chr9 (139565054-139565138)
**624**	*RFX3*	chr9 (3525983-3224646)
**625**	*KLF6*	chr10 (3818188-3827473)
**626**	*AKR1C3*	chr10 (5136568-5149878)
**627**	*NET1*	chr10 (5454518-5500426)
**628**	*GATA3*	chr10 (8096667-8117164)
**629**	*MRC1*	chr10 (17851362-17953178)
**630**	*MLLT10*	chr10 (21823573-22032559)
**631**	*BMI1*	chr10 (22610139-22620414)
**632**	*ABI1*	chr10 (27035524-27150016)
**633**	*RET*	chr10 (43572517-43622952)
**634**	*ALOX5*	chr10 (45869629-45941563)
**635**	*MAPK8*	chr10 (49609687-49643183)
**636**	*ERCC6*	chr10 (50664491-50747147)
**637**	*NCOA4*	chr10 (51565108-51590734)
**638**	*DKK1*	chr10 (54074041-54077417)
**639**	*CCDC6*	chr10 (61548505-61666414)
**640**	*RHOBTB1*	chr10 (62629198-62704033)
**641**	*SIRT1*	chr10 (69644939-69678147)
**642**	*TET1*	chr10 (70320116-70454239)
**643**	*AIFM2*	chr10 (71872023-71892690)
**644**	*PSAP*	chr10 (73576055-73611082)
**645**	*KAT6B*	chr10 (76586170-76792380)
**646**	*ZMIZ1*	chr10 (80828791-81076285)
**647**	*SNCG*	chr10 (88718288-88723017)
**648**	*PTEN*	chr10 (89623195-89728532)
**649**	*LGI1*	chr10 (95517566-95557916)

**650**	*BLNK*	chr10 (97951454-98031333)
**651**	*LOXL4*	chr10 (100007443-100028007)
**652**	*PAX2*	chr10 (102505467-102589698)
**653**	*TLX1*	chr10 (102891060-102897546)
**654**	*BTRC*	chr10 (103113825-103317070)
**655**	*FGF8*	chr10 (103529887-103540126)
**656**	*LDB1*	chr10 (103867325-103880210)
**657**	*NFKB2*	chr10 (104154229-104162281)
**658**	*NT5C2*	chr10 (104847773-104953063)
**659**	*ADD3*	chr10 (111767710-111895323)
**660**	*MXI1*	chr10 (111969989-112047123)
**661**	*PDCD4*	chr10 (112631553-112659764)
**662**	*EIF3A*	chr10 (120794541-120840334)
**663**	*BAG3*	chr10 (121410882-121437329)
**664**	*FGFR2*	chr10 (123237844-123357972)
**665**	*HTRA1*	chr10 (124221041-124274424)
**666**	*DMBT1*	chr10 (124320181-124403252)
**667**	*ADAM12*	chr10 (127702902-128077127)
**668**	*BNIP3*	chr10 (133781204-133795435)
**669**	*ARID5B*	chr10 (63661012-63856707)
**670**	*HRAS*	chr11 (532242-535550)
**671**	*CD151*	chr11 (832952-838835)
**672**	*MUC6*	chr11 (1012824-1036706)
**673**	*MUC2*	chr11 (1074875-1104417)
**674**	*CARS*	chr11 (3022152-3078681)
**675**	*NUP98*	chr11 (3733058-3819022)
**676**	*RRM1*	chr11 (4115924-4160106)
**677**	*EIF3F*	chr11 (8008867-8017719)
**678**	*LMO1*	chr11 (8245850-8290182)
**679**	*HTATIP2*	chr11 (20385289-20405329)
**680**	*FANCF*	chr11 (22644079-22647387)
**681**	*PAX6*	chr11 (31806340-31832879)
**682**	*WT1*	chr11 (32409322-32457081)
**683**	*KIAA1549L (C11orf41)*	chr11 (33563876-33695646)
**684**	*LMO2*	chr11 (33880122-33891509)
**685**	*CD44*	chr11 (35160417-35253949)
**686**	*RAG1*	chr11 (36589562-36601310)
**687**	*RAG2*	chr11 (36613492-36619829)
**688**	*EXT2*	chr11 (44117747-44266980)
**689**	*CD82*	chr11 (44587141-44641315)
**690**	*DDB2*	chr11 (47236493-47260769)
**691**	*SPI1*	chr11 (47376409-47400127)
**692**	*PTPRJ*	chr11 (48002110-48154266)
**693**	*APLNR*	chr11 (57001052-57004927)
**694**	*CLP1*	chr11 (57425215-57429337)
**695**	*CTNND1*	chr11 (57529234-57586652)
**696**	*FEN1*	chr11 (61560109-61564714)
**697**	*MACROD1*	chr11 (63766030-63933585)
**698**	*STIP1*	chr11 (63953586-63972020)
**699**	*FERMT3*	chr11 (63974151-63991363)
**700**	*TRPT1*	chr11 (63991270-63993341)
**701**	*NUDT22*	chr11 (63993737-63997488)
**702**	*DNAJC4*	chr11 (63997752-64001753)
**703**	*VEGFB*	chr11 (64002265-64006259)
**704**	*FKBP2*	chr11 (64008603-64011607)

**705**	*PPP1R14B*	chr11 (64011950-64014413)
**706**	*PLCB3*	chr11 (64018994-64036924)
**707**	*BAD*	chr11 (64037300-64052176)
**708**	*ESRRA*	chr11 (64073698-64084212)
**709**	*MEN1*	chr11 (64570986-64578188)
**710**	*FAU*	chr11 (64888099-64889672)
**711**	*SIPA1*	chr11 (65405578-65418391)
**712**	*RELA*	chr11 (65421067-65430443)
**713**	*KAT5*	chr11 (65479473-65487077)
**714**	*CST6*	chr11 (65779462-65780976)
**715**	*CD248*	chr11 (66081958-66084515)
**716**	*BRMS1*	chr11 (66104804-66112582)
**717**	*RAD9A*	chr11 (67159423-67165883)
**718**	*CHKA*	chr11 (67820326-67888858)
**719**	*LRP5*	chr11 (68080108-68216743)
**720**	*MYEOV*	chr11 (69061622-69064754)
**721**	*CCND1*	chr11 (69455872-69469242)
**722**	*ORAOV1*	chr11 (6948033269490165)
**723**	*NUMA1*	chr11 (71713909-71791739)
**724**	*INPPL1*	chr11 (71935882-71950188)
**725**	*NEU3*	chr11 (74699950-74718743)
**726**	*C11orf30*	chr11 (76156069-76262589)
**727**	*B3GNT6*	chr11 (76745435-76753005)
**728**	*PAK1*	chr11 (77033060-77185108)
**729**	*RSF1*	chr11 (77377274-77531880)
**730**	*THRSP*	chr11 (77774907-77779403)
**731**	*GAB2*	chr11 (77926336-78052926)
**732**	*PICALM*	chr11 (85668213-85780139)
**733**	*EED*	chr11 (85955806-85989785)
**734**	*MRE11A*	chr11 (94150469-94227040)
**735**	*MAML2*	chr11 (95711440-96076344)
**736**	*YAP1*	chr11 (101981192-102104154)
**737**	*BIRC3*	chr11 (102188180-102210135)
**738**	*BIRC2*	chr11 (102217966-102249401)
**739**	*CASP1*	chr11 (104896237-104972158)
**740**	*RAB39*	chr11 (107799276-107834208)
**741**	*NPAT*	chr11 (108028118-108093365)
**742**	*ATM*	chr11 (108093559-108239826)
**743**	*DDX10*	chr11 (108535751-108811657)
**744**	*ARHGAP20*	chr11 (110447766-110583451)
**745**	*POU2AF1*	chr11 (111222981-111250157)
**746**	*CRYAB*	chr11 (111779350-111782473)
**747**	*SDHD*	chr11 (111957571-111966518)
**748**	*ZBTB16*	chr11 (113931287-114121397)
**749**	*NNMT*	chr11 (114166535-114183238)
**750**	*TAGLN*	chr11 (117070040-117075508)
**751**	*TMPRSS4*	chr11 (117947727-117990556)
**752**	*MLL (KMT2A)*	chr11 (118307204-118397539)
**753**	*H2AFX*	chr11 (118964585-118966177)
**754**	*CBL*	chr11 (119076985-119178859)
**755**	*THY1*	chr11 (119288655-119294246)
**756**	*ARHGEF12*	chr11 (120207617-120360645)
**757**	*MIR125B1*	chr11 (121970465-121970552)
**758**	*SPA17*	chr11 (124543740-124564687)
**759**	*ETS1*	chr11 (128328656-128457453)

**760**	*FLI1*	chr11 (128563811-128683162)
**761**	*OPCML*	chr11 (132284875-133402403)
**762**	*EED*	chr11 (85955805-85989785)
**763**	*KDM5A*	chr12 (389222-498621)
**764**	*ERC1*	chr12 (1100404-1605099)
**765**	*FOXM1*	chr12 (2966847-2986321)
**766**	*CCND2*	chr12 (4382901-4414522)
**767**	*VWF*	chr12 (6058039-6233836)
**768**	*CD9*	chr12 (6309482-6347437)
**769**	*ING4*	chr12 (6759704-6772308)
**770**	*ZNF384*	chr12 (6775642-6798738)
**771**	*MIR200C*	chr12 (7072862-7072929)
**772**	*GABARAPL1*	chr12 (10365488-10375724)
**773**	*KLRK1*	chr12 (10524952-10542653)
**774**	*ETV6*	chr12 (11802787-12048325)
**775**	*BCL2L14*	chr12 (12224401-12252627)
**776**	*CDKN1B*	chr12 (12870203-12875316)
**777**	*ATF7IP*	chr12 (14518565-14655869)
**778**	*GUCY2C*	chr12 (14765568-14849519)
**779**	*EPS8*	chr12 (15773075-15942510)
**780**	*RECQL*	chr12 (21621844-21654603)
**781**	*ETNK1*	chr12 (22778075-22797349)
**782**	*SOX5*	chr12 (23685230-24715383)
**783**	*KRAS*	chr12 (25358180-25403854)
**784**	*PPFIBP1*	chr12 (27677044-27848497)
**785**	*PTHLH*	chr12 (28115254-28124916)
**786**	*ATF1*	chr12 (51157789-51214943)
**787**	*HOXC13*	chr12 (54332575-54340328)
**788**	*HOXC11*	chr12 (54366909-54370203)
**789**	*HNRNPA1*	chr12 (54674488-54679030)
**790**	*SARNP*	chr12 (56151054-56211540)
**791**	*PA2G4*	chr12 (56498103-56507694)
**792**	*SMARCC2*	chr12 (56555635-56583351)
**793**	*IL23A*	chr12 (56732663-56734194)
**794**	*GLI1*	chr12 (57853918-57866047)
**795**	*DDIT3*	chr12 (57910371-57914300)
**796**	*AGAP2*	chr12 (58118994-58132029)
**797**	*CDK4*	chr12 (581420030-58146164)
**798**	*USP15*	chr12 (62654187-62799898)
**799**	*HMGA2*	chr12 (66218240-66360071)
**800**	*IL22*	chr12 (68642025-68647281)
**801**	*RAP1B*	chr12 (69004652-69054374)
**802**	*MDM2*	chr12 (69201971-69239212)
**803**	*TSPAN8*	chr12 (71518877-71551779)
**804**	*PAWR*	chr12 (79985745-80084790)
**805**	*KITLG*	chr12 (88886570-88974250)
**806**	*DUSP6*	chr12 (89741837-89746296)
**807**	*BTG1*	chr12 (92534053-92539673)
**808**	*SOCS2*	chr12 (93963598-93969978)
**809**	*METAP2*	chr12 (95867822-95909613)
**810**	*ELK3*	chr12 (96649237-96663613)
**811**	*APAF1*	chr12 (99039078-99129211)
**812**	*SLC5A8*	chr12 (101549994-101604016)
**813**	*DRAM1*	chr12 (102271105-102317401)
**814**	*ASCL1*	chr12 (103351452-103354294)

**815**	*SH2B3*	chr12 (111872665-111889427
**816**	*ALDH2*	chr12 (112204691-112247789)
**817**	*PTPN11*	chr12 (112856535-112947717)
**818**	*HRK*	chr12 (117299027-117319232)
**819**	*PEBP1*	chr12 (118573870-118583390)
**820**	*PRKAB1*	chr12 (120105761-120119429)
**821**	*PXN*	chr12 (120648250-120703563)
**822**	*TRIAP1*	chr12 (120881764-120884215)
**823**	*P2RX7*	chr12 (121570631-121624354)
**824**	*DENR*	chr12 (123237371-123255953)
**825**	*RAN*	chr12 (131356617-131360826)
**826**	*EP400*	chr12 (132434465-132565011)
**827**	*FBRSL1*	chr12 (133067156-133161773)
**828**	*CHFR*	chr12 (133416937-133464204)
**829**	*ZMYM2*	chr13 (20532847-20665984)
**830**	*LATS2*	chr13 (21547176-21635722)
**831**	*PDX1*	chr13 (28494168-28500451)
**832**	*CDX2*	chr13 (28536204-28543505)
**833**	*FLT3*	chr13 (28577410-28674729)
**834**	*PAN3*	chr13 (28712642-28869475)
**835**	*HSPH1*	chr13 (31710763-31736117)
**836**	*BRCA2*	chr13 (32889617-32973809)
**837**	*STARD13*	chr13 (33677272-33780187)
**838**	*LHFP*	chr13 (39917029-40177356)
**839**	*FOXO1*	chr13 (41129801-41240734)
**840**	*ELF1*	chr13 (41506054-41593508)
**841**	*DGKH*	chr13 (42614171-42817033)
**842**	*SERP2*	chr13 (44947977-44971850)
**843**	*LCP1*	chr13 (46700058-46756459)
**844**	*RB1*	chr13 (48877882-49056026)
**845**	*DLEU2*	chr13 (50556687-50699677)
**846**	*TRIM13*	chr13 (50571142-50592603)
**847**	*KCNRG*	chr13 (50589389-50595058)
**848**	*hsa-mir-16-1*	chr13 (50623108-50623197)
**849**	*hsa-mir-15a*	chr13 (50623254-50623337)
**850**	*DLEU1*	chr13 (50656413-50679433)
**851**	*ST13P4*	chr13 (50746153-50747751)
**852**	*DLEU7*	chr13 (51286758-51417885)
**853**	*INTS6*	chr13 (51935701-52027275)
**854**	*OLFM4*	chr13 (53602876-53626196)
**855**	*DACH1*	chr13 (72012097-72441330)
**856**	*KLF5*	chr13 (73633142-73651676)
**857**	*POU4F1*	chr13 (79173230-79177695)
**858**	*GPC5*	chr13 (92050935-93519487)
**859**	*RAP2A*	chr13 (98086475-98120252)
**860**	*ERCC5*	chr13 (103498191-103528351)
**861**	*ANG*	chr14 (21156932-21162345)
**862**	*NDRG2*	chr14 (21484922-21493935)
**863**	*TRA*	chr14 (22090057-23021075)
**864**	*TRA@*	chr14 (22111109-22111806)
**865**	*TRD*	chr14 (22392314-22938606)
**866**	*TRD@*	chr14 (22918107-22925700)
**867**	*MMP14*	chr14 (23305793-23316803)
**868**	*PRKD1*	chr14 (30045687-30396899)
**869**	*STRN3*	chr14 (31363004-31495607)

**870**	*NKX2-1*	chr14 (36985604-36989430)
**871**	*PAX9*	chr14 (37126773-37147011)
**872**	*FOXA1*	chr14 (38059191-38064489)
**873**	*SAV1*	chr14 (51100360-51135023)
**874**	*NIN*	chr14 (51192545-51297839)
**875**	*LGALS3*	chr14 (55595935-55612148)
**876**	*OTX2*	chr14 (57267425-57277184)
**877**	*SIX1*	chr14 (61111417-61116155)
**878**	*ESR2*	chr14 (64693751-64761128)
**879**	*FUT8*	chr14 (65877310-66210839)
**880**	*GPHN*	chr14 (66974124-67648525)
**881**	*ZFP36L1*	chr14 (69254375-69259785)
**882**	*DPF3*	chr14 (73136659-73360809)
**883**	*NUMB*	chr14 (73741918-73925286)
**884**	*DIO2*	chr14 (80663868-80697397)
**885**	*TSHR*	chr14 (81421869-81612646)
**886**	*SEL1L*	chr14 (81939239-82000205)
**887**	*PTPN21*	chr14 (88932122-89021123)
**888**	*GPR68*	chr14 (91698876-91710852)
**889**	*TRIP11*	chr14 (92434242-92506403)
**890**	*TCL6*	chr14 (96117514-96139789)
**891**	*TCL1B*	chr14 (96152754-96158980)
**892**	*TCL1A*	chr14 (96176303-96180533)
**893**	*VRK1*	chr14 (97263684-97347951)
**894**	*BCL11B*	chr14 (99635624-99738050)
**895**	*EML1*	chr14 (100259744-100408395)
**896**	*MEG3*	chr14 (101292445-101327360)
**897**	*TRAF3*	chr14 (103243816-103377837)
**898**	*XRCC3*	chr14 (104163954-104181823)
**899**	*AKT1*	chr14 (105235687-105262080)
**900**	*JAG2*	chr14 (105608076-105635161)
**901**	*MTA1*	chr14 (105886186-105937057)
**902**	*IGH@*	chr14 (106053226-106330470)
**903**	*IGH*	chr14 (106054733-107287769)
**904**	*CEBPE*	chr14 (23588820-23586514)
**905**	*UBE3A*	chr15 (25582396-25684175)
**906**	*ATP10A*	chr15 (25923859-26108349)
**907**	*TRPM1*	chr15 (31293551-31393924)
**908**	*C15orf55*	chr15 (34638066-34649931)
**909**	*SPRED1*	chr15 (38545051-38649450)
**910**	*THBS1*	chr15 (39873280-39889668)
**911**	*BMF*	chr15 (40380091-40401075)
**912**	*BUB1B*	chr15 (40453210-40513337)
**913**	*PLCB2*	chr15 (40580098-40600174)
**914**	*CASC5*	chr15 (40886446-40954881)
**915**	*SPINT1*	chr15 (41136643-41149853)
**916**	*ITPKA*	chr15 (41786055-41795757)
**917**	*LTK*	chr15 (41795839-41806085)
**918**	*TYRO3*	chr15 (41851219-41871536)
**919**	*hsa-mir-626*	chr15 (41983782-41983876)
**920**	*SHC4*	chr15 (49115934-49255641)
**921**	*COPS2*	chr15 (49417471-49447854)
**922**	*MAPK6*	chr15 (52311411-52358462)
**923**	*TCF12*	chr15 (57210833-57580714)
**924**	*ADAM10*	chr15 (58888510-59042177)

**925**	*GCNT3*	chr15 (59903982-59912210)
**926**	*KIAA0101*	chr15 (64657211-64673702)
**927**	*ITGA11*	chr15 (68594042-68724492)
**928**	*PKM2*	chr15 (72491370-72523727)
**929**	*PML*	chr15 (74287013-74335717)
**930**	*GOLGA6A*	chr15 (74362197-74374891)
**931**	*NEIL1*	chr15 (75639331-75647588)
**932**	*CTSH*	chr15 (79214092-79237420)
**933**	*RASGRF1*	chr15 (79252289-79383215)
**934**	*NTRK3*	chr15 (88419987-88799962)
**935**	*IDH2*	chr15 (90627212-90645708)
**936**	*BLM*	chr15 (91260579-91358686)
**937**	*IGF1R*	chr15 (99192761-99507759)
**938**	*AXIN1*	chr16 (337440-402676)
**939**	*TSC2*	chr16 (2097990-2138713)
**940**	*PKD1*	chr16 (2138711-2185899)
**941**	*TRAP1*	chr16 (3708038-3767598)
**942**	*CREBBP*	chr16 (3775055-3930121)
**943**	*USP7*	chr16 (8985951-9057341)
**944**	*CIITA*	chr16 (10971055-11018840)
**945**	*SOCS1*	chr16 (11348274-11350039)
**946**	*ERCC4*	chr16 (14014014-14046205)
**947**	*MYH11*	chr16 (15796991-15950887)
**948**	*ABCC1*	chr16 (16043434-16236930)
**949**	*PLK1*	chr16 (23690201-23701688)
**950**	*IL21R*	chr16 (27438579-27463363)
**951**	*EIF3C*	chr16 (28722782-28747050)
**952**	*MVP*	chr16 (29831715-29859360)
**953**	*MAPK3*	chr16 (30125426-30134630)
**954**	*FUS*	chr16 (31191430-31206192)
**955**	*CYLD*	chr16 (50775961-50835846)
**956**	*RBL2*	chr16 (53468351-53525560)
**957**	*MMP2*	chr16 (55515474-55540586)
**958**	*AMFR*	chr16 (56395364-56459444)
**959**	*CBFB*	chr16 (67063049-67134958)
**960**	*NOL3*	chr16 (67207765-67209643)
**961**	*FAM65A*	chr16 (67562719-67580691)
**962**	*CTCF*	chr16 (67596309-67673088)
**963**	*RLTPR*	chr16 (67679029-67691472)
**964**	*ACD*	chr16 (67691414-67694718)
**965**	*GFOD2*	chr16 (67714618-67753273)
**966**	*RANBP10*	chr16 (67757004-67840555)
**967**	*TSNAXIP1*	chr16 (67841009-67861971)
**968**	*CENPT*	chr16 (67862059-67881361)
**969**	*CDH1*	chr16 (68771195-68869444)
**970**	*TERF2*	chr16 (69389463-69419891)
**971**	*NQO1*	chr16 (69743304-69760533)
**972**	*PHLPP2*	chr16 (71678852-71748704)
**973**	*ZFHX3*	chr16 (72816786-73092534)
**974**	*WWOX*	chr16 (78133327-79246564)
**975**	*MAF*	chr16 (79627744-79634622)
**976**	*CDH13*	chr16 (82660399-83830215)
**977**	*WFDC1*	chr16 (84328401-84363450)
**978**	*FBXO31*	chr16 (87362942-87417394)
**979**	*CDT1*	chr16 (88870186-88875666)

**980**	*CBFA2T3*	chr16 (88941262-89043504)
**981**	*FANCA*	chr16 (89803959-89883065)
**982**	*FAM57A*	chr17 (635847-646075)
**983**	*HIC1*	chr17 (1959604-1962981)
**984**	*TRPV1*	chr17 (3468740-3500336)
**985**	*MYBBP1A*	chr17 (4442191-4458681)
**986**	*ALOX15*	chr17 (4534214-4544960)
**987**	*USP6*	chr17 (5031687-5078324)
**988**	*RABEP1*	chr17 (5185558-5289132)
**989**	*XAF1*	chr17 (6659156-6678964)
**990**	*ALOX12*	chr17 (6899384-6914055)
**991**	*CLDN7*	chr17 (7163222-7166512)
**992**	*SHBG*	chr17 (7533453-7536700)
**993**	*TP53*	chr17 (7571719-7590868)
**994**	*WRAP53*	chr17 (7589389-7606820)
**995**	*KDM6B*	chr17 (7743235-7758118)
**996**	*PER1*	chr17 (8043787-8055753)
**997**	*RPL26*	chr17 (8280834-8286565)
**998**	*GAS7*	chr17 (9813925-9929623)
**999**	*ELAC2*	chr17 (12894929-12921381)
**1000**	*NCOR1*	chr17 (15994883-16097953)
**1001**	*FLCN*	chr17 (17124485-17140502)
**1002**	*MAPK7*	chr17 (19281774-19286857)
**1003**	*SPECC1*	chr17 (19990334-20218072)
**1004**	*KSR1*	chr17 (25799036-25950718)
**1005**	*TRAF4*	chr17 (27071023-27077976)
**1006**	*SUZ12P*	chr17 (29058723-29085353)
**1007**	*CRLF3*	chr17 (29109701-29151778)
**1008**	*NF1*	chr17 (29421944-29704695)
**1009**	*SUZ12*	chr17 (30264044-30328057)
**1010**	*RAD51D*	chr17 (33426811-33446888)
**1011**	*TAF15*	chr17 (34136488-34174238)
**1012**	*AATF*	chr17 (35306175-35414171)
**1013**	*MLLT6*	chr17 (36861872-36886056)
**1014**	*LASP1*	chr17 (37026111-37078023)
**1015**	*PPP1R1B*	chr17 (37784751-37792878)
**1016**	*STARD3*	chr17 (37793333-37820454)
**1017**	*ERBB2*	chr17 (37844393-37884915)
**1018**	*IKZF3*	chr17 (37913967-38020441)
**1019**	*GSDMA*	chr17 (38119226-38134019)
**1020**	*CASC3*	chr17 (38296507-38328431)
**1021**	*CDC6*	chr17 (38444146-38459413)
**1022**	*RARA*	chr17 (38465422-38513895)
**1023**	*GAST*	chr17 (39868613-39872221)
**1024**	*STAT5B*	chr17 (40351194-40428424)
**1025**	*STAT3*	chr17 (40465342-40540513)
**1026**	*BRCA1*	chr17 (41196312-41277500)
**1027**	*ETV4*	chr17 (41605211-41623762)
**1028**	*PYY*	chr17 (42030107-42081837)
**1029**	*SLC4A1*	chr17 (42325758-42345502)
**1030**	*GRN*	chr17 (42422491-42430470)
**1031**	*NMT1*	chr17 (43138680-43186382)
**1032**	*IGF2BP1*	chr17 (47074774-47133507)
**1033**	*DLX4*	chr17 (48050130-48051951)
**1034**	*COL1A1*	chr17 (48261457-48279000)

**1035**	*HLF*	chr17 (53342320-53402426)
**1036**	*MSI2*	chr17 (55333930-55757299)
**1037**	*TRIM37*	chr17 (57060000-57184266)
**1038**	*CLTC*	chr17 (57697050-57774317)
**1039**	*MIR21*	chr17 (57918627-57918698)
**1040**	*PPM1D*	chr17 (58677544-58743640)
**1041**	*BCAS3*	chr17 (58755172-59470199)
**1042**	*TBX2*	chr17 (59477257-59486827)
**1043**	*DDX5*	chr17 (62494374-62502484)
**1044**	*AXIN2*	chr17 (63524683-63557740)
**1045**	*PRKAR1A*	chr17 (66508543-66528910)
**1046**	*SLC9A3R1*	chr17 (72744763-72765499)
**1047**	*GRB2*	chr17 (73314157-73401789)
**1048**	*RECQL5*	chr17 (73646443-73663269)
**1049**	*ST6GALNAC1*	chr17 (74620845-74639894)
**1050**	*SRSF2*	chr17 (74730196-74733493)
**1051**	*SEPT9*	chr17 (75277492-75496678)
**1052**	*TIMP2*	chr17 (76849059-76921472)
**1053**	*ENPP7*	chr17 (77704882-77716021)
**1054**	*RNF213*	chr17 (78234667-78370086)
**1055**	*ASPSCR1*	chr17 (79935426-79975282)
**1056**	*RAC3*	chr17 (79989532-79992080)
**1057**	*SLC16A3*	chr17 (80186282-80197375)
**1058**	*SYNRG*	chr17 (35969486-35874899)
**1059**	*SPAG9*	chr17 (49198226-49039534)
**1060**	*EPB41L3*	chr18 (5392388-5543986)
**1061**	*PTPN2*	chr18 (12785476-12884334)
**1062**	*GATA6*	chr18 (19749416-19782227)
**1063**	*RBBP8*	chr18 (20513295-20606449)
**1064**	*ZNF521*	chr18 (22641887-22932214)
**1065**	*SS18*	chr18 (23596217-23670611)
**1066**	*DSG2*	chr18 (29078027-29128814)
**1067**	*SLC39A6*	chr18 (33690963-33709357)
**1068**	*SETBP1*	chr18 (42260137-42457379)
**1069**	*SMAD2*	chr18 (45359466-45457512)
**1070**	*MAPK4*	chr18 (48086484-48258196)
**1071**	*SMAD4*	chr18 (48556583-48611411)
**1072**	*DCC*	chr18 (49866542-51062273)
**1073**	*MBD2*	chr18 (51677971-51751158)
**1074**	*MALT1*	chr18 (56338618-56417370)
**1075**	*PHLPP1*	chr18 (60382672-60647666)
**1076**	*BCL2*	chr18 (60790578-60986613)
**1077**	*SERPINB5*	chr18 (61144144-61172318)
**1078**	*FSTL3*	chr19 (676388-683392)
**1079**	*STK11*	chr19 (1205798-1228434)
**1080**	*TCF3*	chr19 (1609288-1650286)
**1081**	*GNA11*	chr19 (3094408-3121454)
**1082**	*ZBTB7A*	chr19 (4045216-4066816)
**1083**	*SH3GL1*	chr19 (4360363-4400565)
**1084**	*MLLT1*	chr19 (6210391-6279959)
**1085**	*VAV1*	chr19 (6772722-6857371)
**1086**	*FCER2*	chr19 (7753643-7764365)
**1087**	*ELAVL1*	chr19 (8023457-8070529)
**1088**	*MUC16*	chr19 (8959520-9092018)
**1089**	*DNMT1*	chr19 (10244022-10305755)

**1090**	*ICAM1*	chr19 (10381517-10397291)
**1091**	*TYK2*	chr19 (10461203-10491248)
**1092**	*DNM2*	chr19 (10828728-10942586)
**1093**	*SMARCA4*	chr19 (11071598-11172958)
**1094**	*EPOR*	chr19 (11487880-11495018)
**1095**	*JUNB*	chr19 (12902310-12904125)
**1096**	*CALR*	chr19 (13049413-13055304)
**1097**	*GADD45GIP1*	chr19 (13064972-13068050)
**1098**	*LYL1*	chr19 (13209841-13213974)
**1099**	*CD97*	chr19 (14491956-14519537)
**1100**	*NOTCH3*	chr19 (15270444-15311792)
**1101**	*BRD4*	chr19 (15357847-15391262)
**1102**	*TPM4*	chr19 (16178317-16213813)
**1103**	*JAK3*	chr19 (17935592-17958841)
**1104**	*SLC5A5*	chr19 (17982782-18005983)
**1105**	*JUND*	chr19 (18390563-18392432)
**1106**	*GDF15*	chr19 (18496968-18499986)
**1107**	*ELL*	chr19 (18553472-18632937)
**1108**	*CRTC1*	chr19 (18794425-18893143)
**1109**	*CEBPA*	chr19 (33790839-33793470)
**1110**	*GPI*	chr19 (34856031-34893318)
**1111**	*UBA2*	chr19 (34919267-34960798)
**1112**	*WTIP*	chr19 (34972879-34992085)
**1113**	*FXYD5*	chr19 (35645845-35660788)
**1114**	*ZNF146*	chr19 (36705504-36729675)
**1115**	*PAF1*	chr19 (39876270-39881679)
**1116**	*DYRK1B*	chr19 (40315990-40324841)
**1117**	*AKT2*	chr19 (40736224-40791302)
**1118**	*CYP2A6*	chr19 (41349443-41356352)
**1119**	*AXL*	chr19 (41725108-41767671)
**1120**	*CEACAM1*	chr19 (43011458-43032661)
**1121**	*LYPD3*	chr19 (43964946-43969831)
**1122**	*PLAUR*	chr19 (44150247-44174498)
**1123**	*BCL3*	chr19 (45251977-45263301)
**1124**	*CBLC*	chr19 (45281125-45303903)
**1125**	*RELB*	chr19 (45504707-45541456)
**1126**	*MARK4*	chr19 (45754516-45808541)
**1127**	*ERCC2*	chr19 (45854649-45873845)
**1128**	*PPP1R13L*	chr19 (45882892-45909607)
**1129**	*ERCC1*	chr19 (45910591-45927177)
**1130**	*GRLF1*	chr19 (47421932-47508333)
**1131**	*NPAS1*	chr19 (47524142-47549017)
**1132**	*TMEM160*	chr19 (47549166-47551882)
**1133**	*ZC3H4*	chr19 (47567446-47617009)
**1134**	*BBC3*	chr19 (47724081-47736023)
**1135**	*GLTSCR2*	chr19 (48248793-48260323)
**1136**	*CARD8*	chr19 (48711343-48744320)
**1137**	*BAX*	chr19 (49458117-49464519)
**1138**	*RUVBL2*	chr19 (49497156-49519182)
**1139**	*SCAF1*	chr19 (50145382-50161906)
**1140**	*BCL2L12*	chr19 (50168399-50177173)
**1141**	*ATF5*	chr19 (50432400-50437193)
**1142**	*KLK4*	chr19 (51409608-51413994)
**1143**	*KLK5*	chr19 (51446559-51456344)
**1144**	*KLK7*	chr19 (51479735-51487320)

**1145**	*KLK10*	chr19 (51516000-51523431)
**1146**	*KLK11*	chr19 (51525487-51529872)
**1147**	*MIR125A*	chr19 (52196507-52196592)
**1148**	*TFPT*	chr19 (54610319-54619055)
**1149**	*CNOT3*	chr19 (54641435-54659446)
**1150**	*PEG3*	chr19 (57321445-57352094)
**1151**	*DAZAP1*	chr19 (1407583-1435682)
**1152**	*CEBPG*	chr19 (33864574-33873592)
**1153**	*HNRNPUL1*	chr19 (41768390-41813811)
**1154**	*ZNF274*	chr19 (58694395-58724927)
**1155**	*RASSF2*	chr20 (4760670-4804291)
**1156**	*PLCB1*	chr20 (8113296-8865547)
**1157**	*C20orf94*	chr20 (10415950-10604027)
**1158**	*JAG1*	chr20 (10618332-10654694)
**1159**	*NKX2-2*	chr20 (21491660-21494664)
**1160**	*BCL2L1*	chr20 (30252261-30310656)
**1161**	*PLAGL2*	chr20 (30780307-30795546)
**1162**	*ASXL1*	chr20 (30946146-30960352)
**1163**	*NOL4L (C20orf112)*	chr20 (31030861-31172875)
**1164**	*MAPRE1*	chr20 (31407699-31438211)
**1165**	*CBFA2T2*	chr20 (32077927-32237837)
**1166**	*E2F1*	chr20 (32263292-32274210)
**1167**	*SRC*	chr20 (35973088-36033821)
**1168**	*BLCAP*	chr20 (36145819-36156333)
**1169**	*MAFB*	chr20 (39314487-39317880)
**1170**	*TOP1*	chr20 (39657461-39753126)
**1171**	*MYBL2*	chr20 (42295709-42345122)
**1172**	*WISP2*	chr20 (43343885-43356452)
**1173**	*STK4*	chr20 (43595120-43708593)
**1174**	*UBE2C*	chr20 (44441255-44445596)
**1175**	*MMP9*	chr20 (44637547-44645200)
**1176**	*NCOA3*	chr20 (46130601-46285621)
**1177**	*CSE1L*	chr20 (47662838-47713486)
**1178**	*PTGIS*	chr20 (48120411-48184707)
**1179**	*SNAI1*	chr20 (48599513-48605420)
**1180**	*BCAS4*	chr20 (49411467-49493714)
**1181**	*ZNF217*	chr20 (52183610-52199636)
**1182**	*AURKA*	chr20 (54944445-54967351)
**1183**	*SS18L1*	chr20 (60718822-60757566)
**1184**	*EEF1A2*	chr20 (62119366-62130505)
**1185**	*TNFRSF6B*	chr20 (62328004-62330051)
**1186**	*CEBPB*	chr20 (48807119-48809227)
**1187**	*NRIP1*	chr21 (16333556-16437126)
**1188**	*MIR125B2*	chr21 (17962557-17962645)
**1189**	*ADAMTS1*	chr21 (28208606-28217728)
**1190**	*TIAM1*	chr21 (32490736-32931290)
**1191**	*OLIG2*	chr21 (34398215-34401503)
**1192**	*RUNX1*	chr21 (36160097-36421595)
**1193**	*DYRK1A*	chr21 (38792602-38887679)
**1194**	*ERG*	chr21 (39751949-39870428)
**1195**	*ETS2*	chr21 (40177849-40196878)
**1196**	*TMPRSS2*	chr21 (42836478-42879992)
**1197**	*TFF3*	chr21 (43731777-43735706)
**1198**	*TFF2*	chr21 (43766467-43771208)
**1199**	*TFF1*	chr21 (43782391-43786644)

**1200**	*TMPRSS3*	chr21 (43791996-43816200)
**1201**	*U2AF1*	chr21 (44513065-44527688)
**1202**	*CSTB*	chr21 (45193831-45196259)
**1203**	*PTTG1IP*	chr21 (46269500-46293818)
**1204**	*S100B*	chr21 (48018531-48025035)
**1205**	*ERG*	chr21 (40033618-39739182)
**1206**	*CLTCL1*	chr22 (19166987-19279239)
**1207**	*SEPT5*	chr22 (19705958-19710845)
**1208**	*MAPK1*	chr22 (22113947-22221970)
**1209**	*IGL@*	chr22 (22380474-23265085)
**1210**	*VPREB1*	chr22 (22599191-22599927)
**1211**	*BCR*	chr22 (23522551-23660224)
**1212**	*MMP11*	chr22 (24115036-24126503)
**1213**	*SMARCB1*	chr22 (24129150-24176705)
**1214**	*MIF*	chr22 (24236565-24237409)
**1215**	*MN1*	chr22 (28144264-28197486)
**1216**	*CHEK2*	chr22 (29083731-29137822)
**1217**	*EWSR1*	chr22 (29663998-29696515)
**1218**	*NF2*	chr22 (29999545-30094589)
**1219**	*MCM5*	chr22 (35796116-35820495)
**1220**	*MYH9*	chr22 (36677322-36784112)
**1221**	*IL2RB*	chr22 (37521879-37545962)
**1222**	*RAC2*	chr22 (37621310-37640305)
**1223**	*SOX10*	chr22 (38368319-38380539)
**1224**	*PDGFB*	chr22 (39619685-39640957)
**1225**	*ATF4*	chr22 (39916569-39918691)
**1226**	*MKL1*	chr22 (40806284-40859444)
**1227**	*RBX1*	chr22 (41347351-41369019)
**1228**	*EP300*	chr22 (41488613-41576081)
**1229**	*XRCC6*	chr22 (42017295-42060052)
**1230**	*PARVB*	chr22 (44395173-44565103)
**1231**	*FBLN1*	chr22 (45898719-45959242)
**1232**	*BRD1*	chr22 (50166925-50221196)
**1233**	*MAPK12*	chr22 (50691331-50700089)
**1234**	*TYMP*	chr22 (50964182-50968514)
**1235**	*IGLL1*	chr22 (23922495-23915312)
**1236**	*CRLF2*	chrX (1314893-1331616)
**1237**	*IL3RA*	chrX (1455509-1501582)
**1238**	*P2RY8*	chrX (1581465-1656037)
**1239**	*ZRSR2*	chrX (15808573-15841382)
**1240**	*RBBP7*	chrX (16862775-16887978)
**1241**	*REPS2*	chrX (16964814-17171403)
**1242**	*BCOR*	chrX (39910498-40036582)
**1243**	*KDM6A*	chrX (44732420-44971857)
**1244**	*MIR221*	chrX (45605585-45605694)
**1245**	*MIR222*	chrX (45606421-45606530)
**1246**	*GATA1*	chrX (48644982-48652717)
**1247**	*TFE3*	chrX (48886242-48900990)
**1248**	*FOXP3*	chrX (49106897-49121288)
**1249**	*SSX2*	chrX (52780308-52790617)
**1250**	*FAM123B*	chrX (63404997-63425624)
**1251**	*MSN*	chrX (64887511-64961793)
**1252**	*AR*	chrX (66763874-66944119)
**1253**	*FOXO4*	chrX (70315998-70323384)
**1254**	*NONO*	chrX (70503042-70521018)

**1255**	*CXCR3*	chrX (70835766-70838367)
**1256**	*BRWD3*	chrX (79924987-80065233)
**1257**	*DACH2*	chrX (85403454-86087605)
**1258**	*BTK*	chrX (100604435-100641212)
**1259**	*ARMCX1*	chrX (100805514-100809675)
**1260**	*ARMCX3*	chrX (100878120-100882831)
**1261**	*ARMCX2*	chrX (100910268-100914863)
**1262**	*AMOT*	chrX (112018105-112066354)
**1263**	*SEPT6*	chrX (118750909-118827333)
**1264**	*RHOXF2*	chrX (119206240-119211707)
**1265**	*ZBTB33*	chrX (119384610-119392251)
**1266**	*STAG2*	chrX (123094409-123236505)
**1267**	*ELF4*	chrX (129198894-129244688)
**1268**	*AIFM1*	chrX (129263338-129272015)
**1269**	*GPC3*	chrX (132669776-133119673)
**1270**	*PHF6*	chrX (133507341-133562822)
**1271**	*SPANXA1*	chrX (140677834-140678899)
**1272**	*L1CAM*	chrX (153126971-153141399)
**1273**	*RPL10*	chrX (153626405-153630680)
**1274**	*DKC1*	chrX (153991031-154005964)
**1275**	*MTCP1*	chrX (154292308-154299547)
**1276**	*OFD1*	chrX (13752831-13787480)

Supplementary Table S2. Number of aneuploidies and LOH of different chromosomes in B-ALL.

**Number of chromosomes**	Trisomy/ Tetrasomy	Single LOH	Combined LOH	All LOH
**1**	1/1q -8	1	6	7
**2**	6	0	2	2
**3**	6	1	5	6
**4**	76	1	0	1
**5**	17	0	4	4
**6**	72	1	0	1
**7**	14	0	3	3
**8**	40	3	3	6
**9**	**9**	**26**	**7**	**33**
**10**	64	0	2	2
**11**	9	2	7	9
**12**	18	1	3	4
**13**	3	2	7	9
**14**	**80**	**0**	**0**	**0**
**15**	8	0	7	7
**16**	8	1	6	7
**17**	**70**	**0**	**0**	**0**
**18**	**76**	**0**	**0**	**0**
**19**	2	1	5	6
**20**	1	1	4	5
**21**	**89**	**0**	**0**	**0**
**22**	9	1	1	2
**X**	85	0	0	0

Supplementary Table S3. The associations (only significant) between the dimension and the studied parameters in MCA in patients with B-ALL.

Dimension 1				*Dimension 2*		
Parameter	*R^2*	*p*		*Parameter*	*R^2*	*p*
LOH segments	0.63	**< 0.001**		LOH 9/9p	0.74	**< 0.001**
hyperdiploidy	0.59	**< 0.001**		del *CDKN2A*	0.72	**< 0.001**
del *CDKN2A*	0.62	**< 0.001**		LOH segments	0.61	**< 0.001**
LOH 9/9p	0.56	**< 0.001**		Age	0.09	**< 0.001**
response on prednisone	0.14	**< 0.001**		MRD FMC 15 day	0.07	**0.001**
Age	0.14	**< 0.001**		BM	0.05	**0.002**
MRD FMC 15 day	0.06	**0.031**		-	-	**-**

This table presents only statistically significant associations ($p < 0.05$) between the studied variables and the first two dimensions identified through Multiple Correspondence Analysis (MCA) in the B-ALL cohort. Dimension 1 is primarily associated with genetic variables, including LOH segments ($R^2 = 0.63$), del CDKN2A ($R^2 = 0.62$), hyperdiploidy ($R^2 = 0.59$), and LOH 9/9p ($R^2 = 0.56$). Among clinical variables, weaker but significant associations were observed with response to prednisone ($R^2 = 0.14$), age ($R^2 = 0.14$), and MRD by flow cytometry at day 15 ($R^2 = 0.06$). Dimension 2 is most strongly associated with LOH 9/9p ($R^2 = 0.74$), del *CDKN2A* ($R^2 = 0.72$), and LOH segments ($R^2 = 0.61$). Additionally, age ($R^2 = 0.09$), MRD on day 15 ($R^2 = 0.07$), and bone marrow blast percentage at diagnosis ($R^2 = 0.05$) were also significantly linked.
Abbreviations: MCA – Multiple Correspondence Analysis; B-ALL – B-cell acute lymphoblastic leukemia; LOH – loss of heterozygosity; del *CDKN2A* – deletion of the *CDKN2A* gene; MRD – minimal residual disease; FMC – flow cytometry; BM – percentage of blasts in bone marrow at diagnosis; R^2 – determination coefficient; p – p-value of statistical test.

Supplementary Table S4. Describing clusters by the individual variables (significant results only) in patients with B-ALL.

Parameter	*df*	*p*
Del *CDKN2A*	8	**< 0.001**
LOH 9/9p	10	**< 0.001**
LOH segments	8	**< 0.001**
Hyperdiploidy	4	**< 0.001**

Parameter	*df*	*p*
Age	2	**< 0.001**
Response of the prednisone	2	**0.001**

This table presents statistically significant associations between the identified clusters (from MCA-based clustering) and specific clinical or genetic parameters in the B-ALL cohort. Only variables showing significant differences between clusters are included ($p < 0.05$). The strongest associations were observed for key genetic features such as *CDKN2A* deletion ($p < 0.001$), LOH on chromosome 9 or 9p ($p < 0.001$), and LOH segments ($p < 0.001$), indicating their major role in differentiating between patient subgroups. Additionally, hyperdiploidy, age, and prednisone response were also significantly associated with cluster assignment, suggesting that both genomic alterations and early treatment response contribute to the biological stratification of B-ALL patients.
Abbreviations: MCA – Multiple Correspondence Analysis; B-ALL – B-cell acute lymphoblastic leukemia; LOH – loss of heterozygosity; del *CDKN2A* – deletion of the *CDKN2A* gene; df – degrees of freedom; p – p-value of the statistical test.

Supplementary Table S5. Describing clusters by the individual categories (significant results only) in patients with B-ALL.

Category	*Cla/Mod*	*Mod/Cla*	*Global*	*v-test*	*p*
Cluster 1					
LOH 9	87.88	96.67	27.50	9.73	**< 0.001**
CDKN2A: LOH	100.00	80.00	20.00	9.15	**< 0.001**
LOH segments: LOH 9	94.44	56.67	15.00	6.80	**< 0.001**
hyperdiploidy: high	34.48	100.00	72.50	4.38	**< 0.001**
Patients with interrupted 9p LOH	0.00	0.00	8.33	-1.97	**0.049**
LOH segments: LOH of one chromosome with exclusion of chromosome 9	0.00	0.00	10.00	-2.23	**0.026**
hyperdiploidy: low	0.00	0.00	10.00	-2.23	**0.026**
CDKN2A: duplication	0.00	0.00	11.67	-2.47	**0.014**
CDKN2A: del biallelic	0.00	0.00	15.83	-3.02	**0.002**
diploid karyotype	0.00	0.00	17.50	-3.24	**0.001**
LOH segmented	5.45	10.00	45.83	-4.70	**< 0.001**

Category	*Cla/Mod*	*Mod/Cla*	*Global*	*v-test*	*p*
CDKN2A: normal	0.00	0.00	32.50	-4.93	**< 0.001**
No evidence of LOH 9/9p	0.00	0.00	52.50	-7.16	**< 0.001**
Cluster 2					
No evidence of LOH 9/9p	74.60	87.04	52.50	7.03	**< 0.001**
CDKN2A: normal	82.05	59.26	32.50	5.69	**< 0.001**
hyperdiploidy: high	57.47	92.59	72.50	4.58	**< 0.001**
CDKN2A: duplication	100.00	25.92	11.67	4.58	**< 0.001**
LOH segments: LOH of one chromosome with exclusion of chromosome 9	100.00	22.22	10.00	4.15	**< 0.001**
LOH segments: LOH of several chromosomes	75.00	33.33	20.00	3.25	**0.001**
age: ≤ 5 yrs	59.01	66.67	50.83	3.10	**0.002**
response on prednisone: good	52.22	87.03	75.00	2.74	**0.006**
LOH segmented	32.73	33.33	45.83	-2.45	**0.014**
MRD FMC 15 day: ≥ 10%	9.09	1.85	9.17	-2.52	**0.012**
hyperdiploidy: low	8.33	1.85	10.00	-2.72	**0.006**
response on prednisone: poor	23.33	12.96	25.00	-2.74	**0.006**
age: > 5 yrs.	30.51	33.33	49.17	-3.10	**0.002**
Patients with interrupted 9p LOH	0.00	0.00	8.33	-3.11	**0.002**
diploid karyotype	14.29	5.55	17.50	-3.15	**0.002**
LOH segments: LOH 9	0.00	0.00	15.00	-4.51	**< 0.001**
CDKN2A: del biallelic	0.00	0.00	15.83	-4.67	**< 0.001**
CDKN2A: LOH	0.00	0.00	20.00	-5.42	**< 0.001**
LOH 9	3.03	1.85	27.50	-6.09	**< 0.001**
Cluster 3					
LOH segmented	61.81	94.44	45.83	7.28	**< 0.001**

Category	*Cla/Mod*	*Mod/Cla*	*Global*	*v-test*	*p*
CDKN2A: del biallelic	100.00	52.78	15.83	7.08	**< 0.001**
diploid karyotype	85.71	50.00	17.50	5.80	**< 0.001**
Patients with interrupted 9p LOH	100.00	27.78	8.33	4.73	**< 0.001**
hyperdiploidy: low	91.67	30.56	10.00	4.56	**< 0.001**
age: > 5 yrs.	47.46	77.78	49.17	4.10	**< 0.001**
response on prednisone: poor	56.67	47.22	25.00	3.50	**< 0.001**
BM: > 91%	38.98	63.89	49.17	2.07	**< 0.001**
CDKN2A: normal	17.95	19.44	32.50	-1.99	**< 0.001**
BM: ≤ 91%	21.31	36.11	50.83	-2.07	**< 0.001**
LOH segments: LOH of one chromosome with exclusion of chromosome 9	0.00	0.00	10.00	-2.55	**< 0.001**
LOH segments: LOH 9	5.56	2.78	15.00	-2.57	**< 0.001**
CDKN2A: duplication	0.00	0.00	11.67	-2.82	**< 0.001**
LOH 9	9.09	8.33	27.50	-3.19	**< 0.001**
response on prednisone: good	21.11	52.78	75.00	-3.50	**< 0.001**
LOH segments: LOH of several chromosomes	0.00	0.00	20.00	-4.01	**< 0.001**
CDKN2A: LOH	0.00	0.00	20.00	-4.02	**< 0.001**
age: ≤ 5 yrs	13.11	22.22	50.83	-4.10	**< 0.001**
hyperdiploidy: high	8.04	19.44	72.50	-8.40	**< 0.001**

Note: "Cla/Mod" – the prevalence of patients exhibiting a specific characteristic within the designated cluster, highlighting how common that trait is among individuals classified in that group. "Mod/Cla" indicates the number of individuals within the cluster who are characterized by the studied parameter, providing insight into the distribution of that parameter among the cluster's population. "Global" –the overall prevalence of the studied category across the entire sample. The "v-test" serves as a statistical measure that quantifies the strength of the association or difference observed between the clusters concerning the studied parameter. A higher v-test value signifies a more pronounced difference, suggesting a significant relationship between the parameter and the cluster classification. *P*-value (*p*) – the statistical significance of the finding

Supplementary Table S6. The associations (only significant) between the dimension and the studied parameters in MCA in patients with T-ALL.

Dimension 1				*Dimension 2*		
Parameter	R^2	*p*		*Parameter*	R^2	*p*
del *CDKN2A*	0.56	< 0.001		BM	0.42	< 0.001
LOH 9p	0.50	< 0.001		response on prednisone	0.35	< 0.001
MRD FMC 15 day	0.44	< 0.001		del *CDKN2A*	0.27	0.001
MRD PCR 33 day	0.37	< 0.001		MRD FMC 15 day	0.23	0.001
response on prednisone	0.22	< 0.001		MRD PCR 33 day	0.21	0.001
WBC	0.19	0.001		Age	0.16	0.002
Age	0.12	0.008		WBC	0.13	0.005
Follow-up	0.10	0.018		Relapse	0.17	0.007
Relapse	0.13	0.023		Gender	0.09	0.019

This table presents only statistically significant associations ($p < 0.05$) between the studied variables and the first two dimensions identified through Multiple Correspondence Analysis (MCA) in the T-ALL cohort.
Dimension 1 is primarily driven by genetic and early treatment response markers, including *CDKN2A* deletion ($R^2 = 0.56$), LOH on 9p ($R^2 = 0.50$), and MRD levels by flow cytometry on day 15 (FMC) ($R^2 = 0.44$). Additional contributors include MRD on day 33 (PCR), response to prednisone, WBC, age, relapse, and follow-up duration. Dimension 2 reflects a mix of clinical outcome and demographic variables, most notably bone marrow blast percentage at diagnosis ($R^2 = 0.42$), response to prednisone ($R^2 = 0.35$), *CDKN2A* deletion ($R^2 = 0.27$), and MRD values. Other significantly associated variables include age, WBC, relapse occurrence, and gender. These findings highlight that both dimensions are shaped by a combination of genetic alterations (e.g., del *CDKN2A*, LOH 9p) and clinical characteristics, particularly early treatment response indicators like MRD and prednisone sensitivity. The multidimensional structure provides insight into distinct patient subgroups within T-ALL.
Abbreviations: MCA – Multiple Correspondence Analysis; T-ALL – T-cell acute lymphoblastic leukemia; LOH 9p – loss of heterozygosity on the short arm of chromosome 9; del *CDKN2A* – deletion of the *CDKN2A* gene; MRD – minimal residual disease; FMC – flow cytometry; PCR – polymerase chain reaction; BM – percentage of blasts in bone marrow at diagnosis; WBC - white blood cell count; R^2 – determination coefficient; p – p-value of statistical test.

Supplementary Table S7. Describing clusters by the individual variables (significant results only) in patients with T-ALL.

Parameter	*df*	*p*
Del *CDKN2A*	6	**< 0.001**
MRD FMC 15 day	4	**< 0.001**
LOH 9p	6	**< 0.001**
MRD/PCR 33 day	4	**< 0.001**
Response of the prednisone	2	**0.007**
Gender	2	**0.025**
WBC	2	**0.037**

This table presents clinical and genetic variables that showed statistically significant associations ($p < 0.05$) with patient clusters identified in the T-ALL cohort. The associations were assessed using appropriate statistical tests, and only variables with significant results are listed. The most strongly associated features include: deletion of the *CDKN2A* gene ($p < 0.001$), MRD measured by flow cytometry at day 15 ($p < 0.001$), LOH on chromosome 9p ($p < 0.001$), MRD measured by PCR at day 33 ($p < 0.001$). Other significantly associated parameters include: response to prednisone ($p = 0.007$), gender ($p = 0.025$), white blood cell count at diagnosis (WBC) ($p = 0.037$). These results indicate that both genetic markers (del *CDKN2A*, LOH 9p) and treatment response indicators (MRD, prednisone response) play a key role in defining distinct molecular and clinical subgroups within the T-ALL cohort.

Abbreviations: T-ALL – T-cell acute lymphoblastic leukemia; LOH – loss of heterozygosity; del *CDKN2A* – deletion of the *CDKN2A* gene; MRD – minimal residual disease; FMC – flow cytometry; PCR – polymerase chain reaction; WBC - white blood cell count; df – degrees of freedom; p – p-value of the statistical test.

Supplementary Table S8. Describing clusters by the individual categories (significant results only) in patients with T-ALL.

Category	*Cla/Mod*	*Mod/Cla*	*Global*	*v-test*	*p*
Cluster 1					
MDR FMC 15 day: < 0.1%	90.00	83.33	19.97	5.68	**< 0.001**
Response on prednisone: good	33.33	100.00	62.07	3.19	**0.001**
MRD/PCR 33 day: negative	66.67	50.00	15.52	3.17	**0.001**
CDKN2A: LOH	100.00	25.00	5.17	2.69	**0.007**
MRD FMC 15 day: 0.1-10%	4.76	8.33	36.20	-2.25	**0.024**

Category	*Cla/Mod*	*Mod/Cla*	*Global*	*v-test*	*p*
No evidence of LOH at 9p	0.00	0.00	24.14	-2.26	**0.024**
MRD FMC 15 day: ≥10%	3.85	8.33	44.83	-2.86	**0.004**
response on prednisone: poor	0.00	0.00	37.93	-3,19	**0.001**
Cluster2					
del *CDKN2A*: biallelic	80.00	77.77	60.34	3.37	**0.001**
MRD/PCR 33 day: positive	78.37	80.55	63.79	3.28	**0.001**
MRD FMC 15 day: 0.1-10%	85.71	50.00	36.21	2.77	**0.005**
gender: male	76.47	72.22	58.62	2.60	**0.009**
CDKN2A: LOH	0.00	0.00	5.17	-1.97	**0.050**
No evidence of LOH at 9p	35.71	13.88	24.13	-2,21	**0.027**
MRD/PCR 33 day: negative	22.22	5.56	15.52	-2.50	**0.012**
gender: female	41.67	27.78	41.37	-2.60	**0.009**
CDKN2A: normal	0.00	0.00	13.79	-3.76	**< 0.001**
MRD FMC 15 day: < 0.1%	9.09	2.78	18.97	-3.87	**< 0.001**
Cluster 3					
CDKN2A: normal	100.00	90.00	13.79	5.58	**< 0.001**
No evidence of LOH at 9p	64.29	90.00	24.13	4.78	**< 0.001**
MRD/PCR 33 day: other (no marker)	50.00	60.00	20.69	2.93	**0.003**
MRD FMC 15 day: ≥10%	30.77	80.00	44.83	2.34	**0.019**
WBC: ≤ 117.86 10^3/uL	27.59	80.00	50.00	2.00	**0.045**
age: > 10.33 yrs.	27.59	80.00	50.00	2.00	**0.045**
WBC: > 117.86 10^3/uL	6.90	20.00	50.00	-2.00	**0.045**
age: ≤10.33 yrs.	6.90	20.00	50.00	-2.00	**0.045**

Category	*Cla/Mod*	*Mod/Cla*	*Global*	*v-test*	*p*
Patients with interrupted 9p LOH	4.34	10.00	39.66	-2.07	**0.038**
LOH 9p	0.00	0	25.86	-2.08	**0.037**
MRD/PCR 33 day: positive	8.11	30.00	63.79	-2.27	**0.023**
del *CDKN2A*: biallelic	0.00	0	60.34	-4.24	**< 0.001**

Note: "Cla/Mod" – the prevalence of patients exhibiting a specific characteristic within the designated cluster, highlighting how common that trait is among individuals classified in that group. "Mod/Cla" indicates the number of individuals within the cluster who are characterized by the studied parameter, providing insight into the distribution of that parameter among the cluster's population. "Global" –the overall prevalence of the studied category across the entire sample. The "v-test" serves as a statistical measure that quantifies the strength of the association or difference observed between the clusters concerning the studied parameter. A higher v-test value signifies a more pronounced difference, suggesting a significant relationship between the parameter and the cluster classification. P-value (p) – the statistical significance of the findings.

Supplementary Table S9. Karyotypes with chromothripsis in patients of both types of leukemia.

Type of ALL	B-ALL	T-ALL
Molecular karyotype	1) arr[GRCh37](X,4,5)x3,(7)cth,(8)x3,(9p)x2hmz,<br>(9q)x3,12p13.2p12.3(11734418_15697806)x1,<br>(14)x2~3.17p13.1p12(8750694_15178622)x1,<br>(18)x3,(21)x4<br>2) arr[GRCh37]3p25.1q29(16256377_197851986)x1,<br>5p15.33p13.2(113577_34578602)x1,<br>5p13.2q35.3(34453884_180719789)x3,8p23.3p11.1<br>(158049_43410101)x1,8q21.3q22.1<br>(87278555_96947892)x1,9p22.3p13.1<br>(15923449_38760557)x1,(13)x1,<br>17p13.3p11.2(526_21214496)x1,(21,X)x3	1) arr[GRCh37]1p36.33p36.31(849466_6445105)x1-2<br>hmz,1p36.22p31.1(10387929_76236452)x2-<br>3,1p31.1p12(79915304_120001330)x1-2<br>hmz,1q21.2q44(148514235_249224684)x2-<br>3.5q31.1q35.3(130697570_180719789)x1-2<br>hmz,7q31.1q36.3(108354488_159119707)cth,9p23p21.1(10407[illegible]<br>4_32282978)x1 hmz,9p21.1p13.2(32158191_38326473)x2-<br>3.9q21.11q33.2(71512667_123009219)x1-2<br>hmz,9q33.2q34.3(124629919_141020389)x2-<br>3.12q24.11q24.33(111548325_133778166)x2 hmz,13<br>q13.3q21.33(40010662_70747804)x1-2 hmz
Chromothripsis	1) 7p14.2q36.1 (35,962,828_148,970,666)<br>2) 12p13.2q24.33 (11,685,609__133,667,021)	chromosomes 1 and 9

Supplementary S2.2 Figures

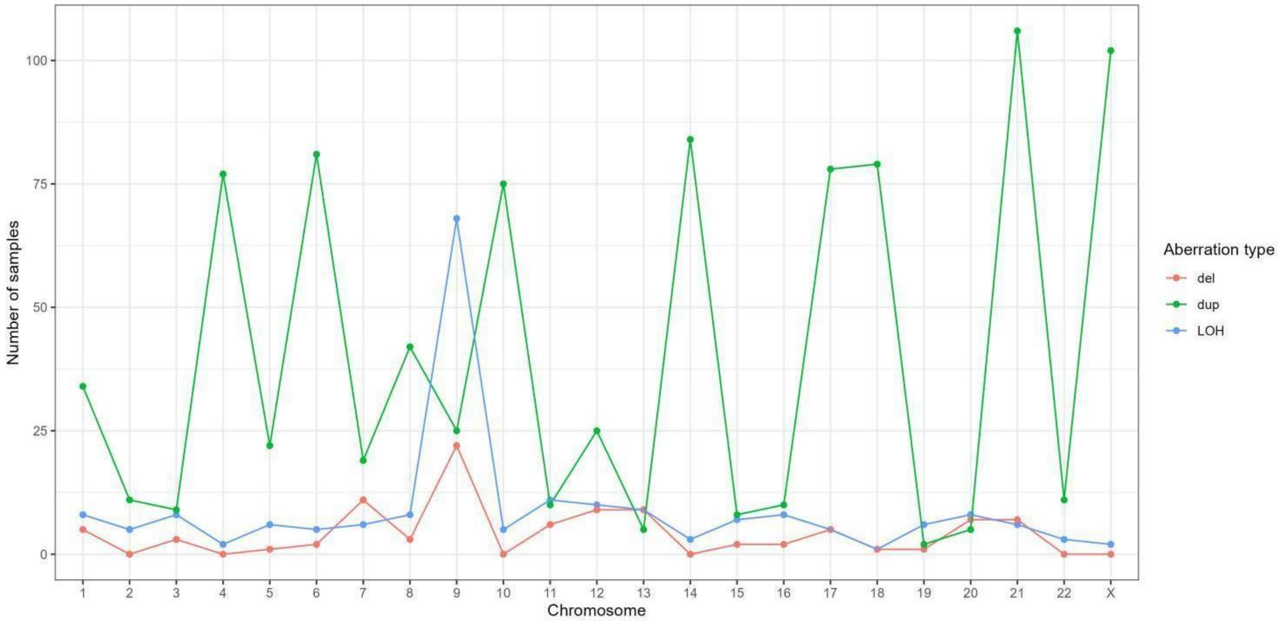

Supplementary Figure S1. Number of B-ALL samples with aberration in each chromosome.

This line plot illustrates the number of samples presenting specific types of chromosomal aberrations—deletions (del), duplications (dup), and loss of heterozygosity (LOH)—across chromosomes 1 to 22 and the X chromosome.

Green line (dup): Duplication events were the most frequent, particularly prominent on chromosomes 4, 6, 8, 10, 14, 16, 18, 21, and X, with some exceeding 100 samples.

Blue line (LOH): LOH was observed with highest frequency on chromosomes 9 and 10, and appeared consistently across the genome in a moderate number of cases.

Red line (del): Deletions occurred less frequently but were still present across nearly all chromosomes, with notable peaks on chromosomes 9 and 12.

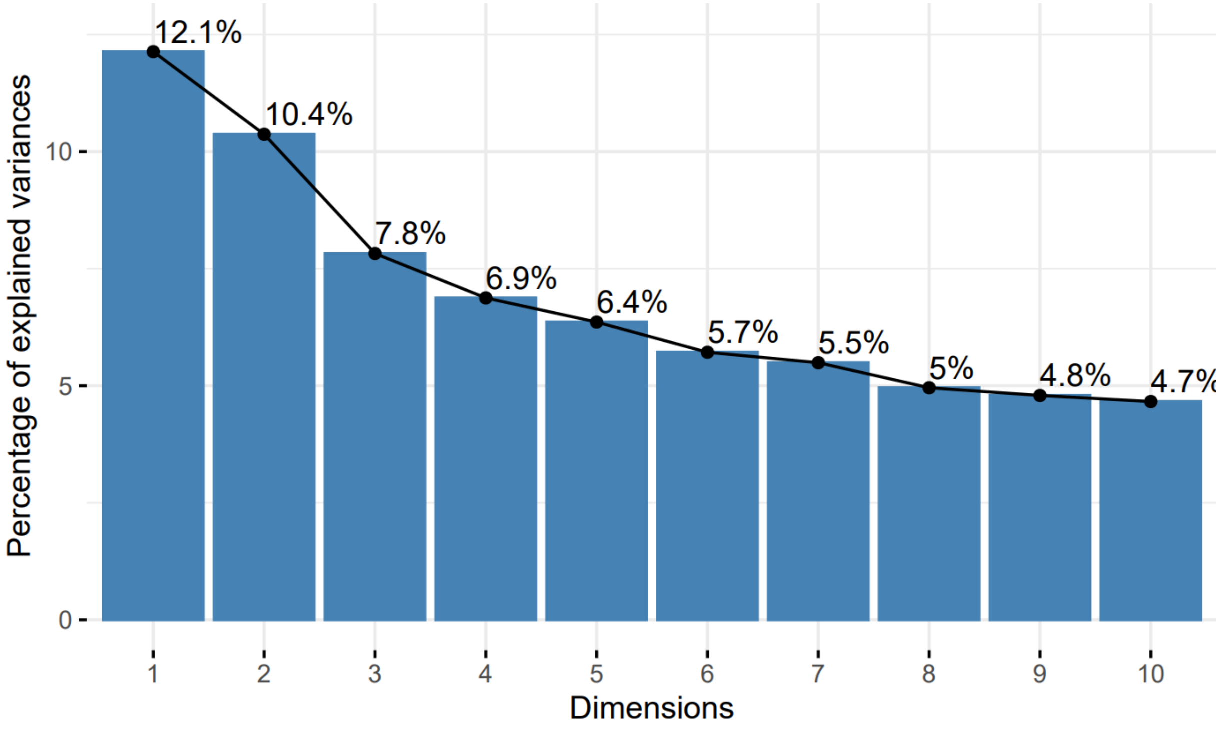

Supplementary Figure S2. The variance of the nine analyzed variables, through the application of Multiple Correspondence Analysis (MCA), was fully explained within a 23-dimensional framework. Scree plot showing explained variance percentages for the first 10 dimensions in patients with B-ALL.

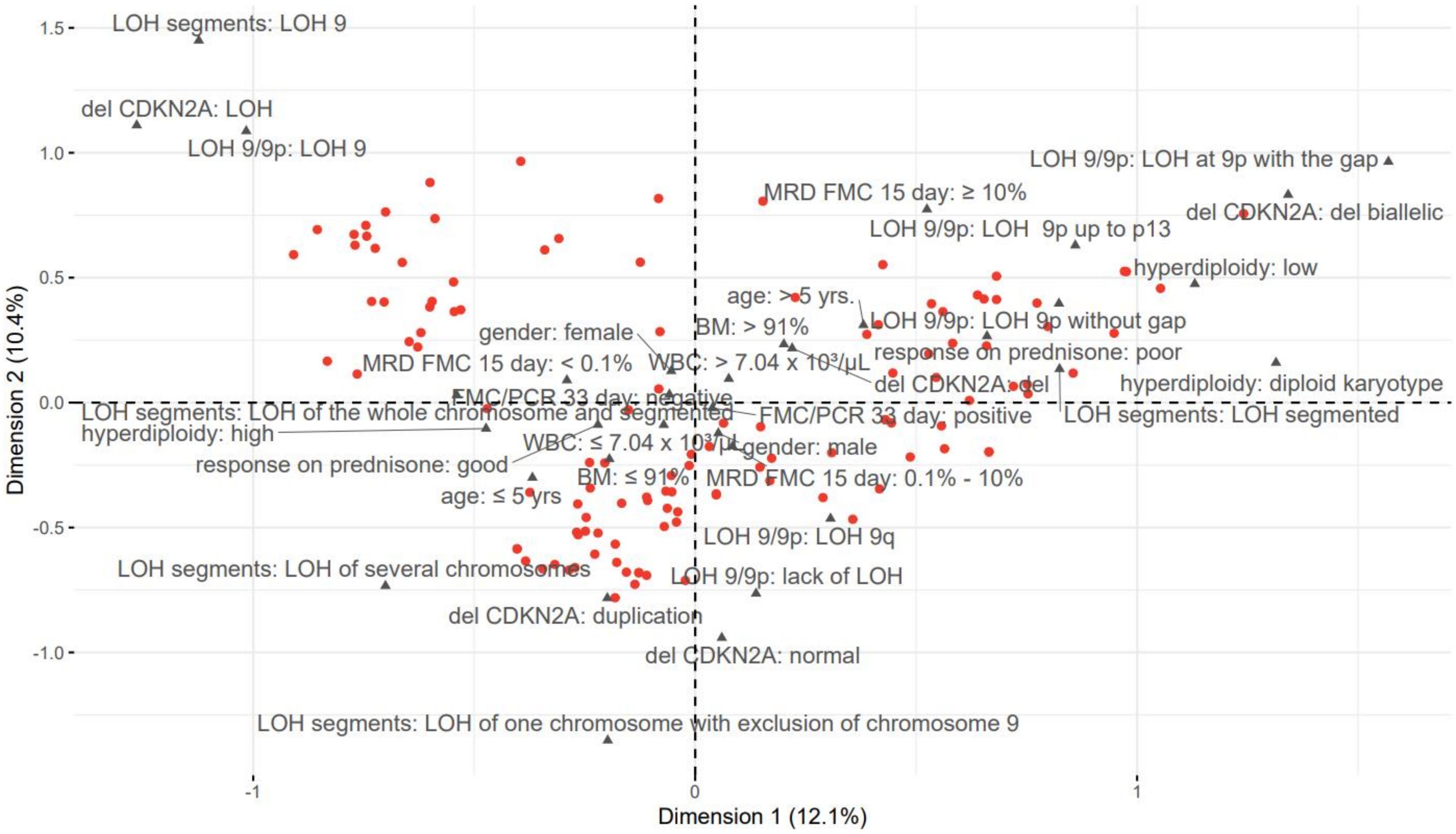

Supplementary Figure S3. Global data patterns in two-dimensional plot in patients with B-ALL (biplot): patient results (red points) and parameter categories (gray triangles).

Supplementary Figure S4-S7. These figures provide a visual representation of how patients are grouped based on their characteristics, allowing for a comprehensive understanding of the relationships between the parameters and the dimensions identified through the analysis with drawing confidence ellipses around categories.

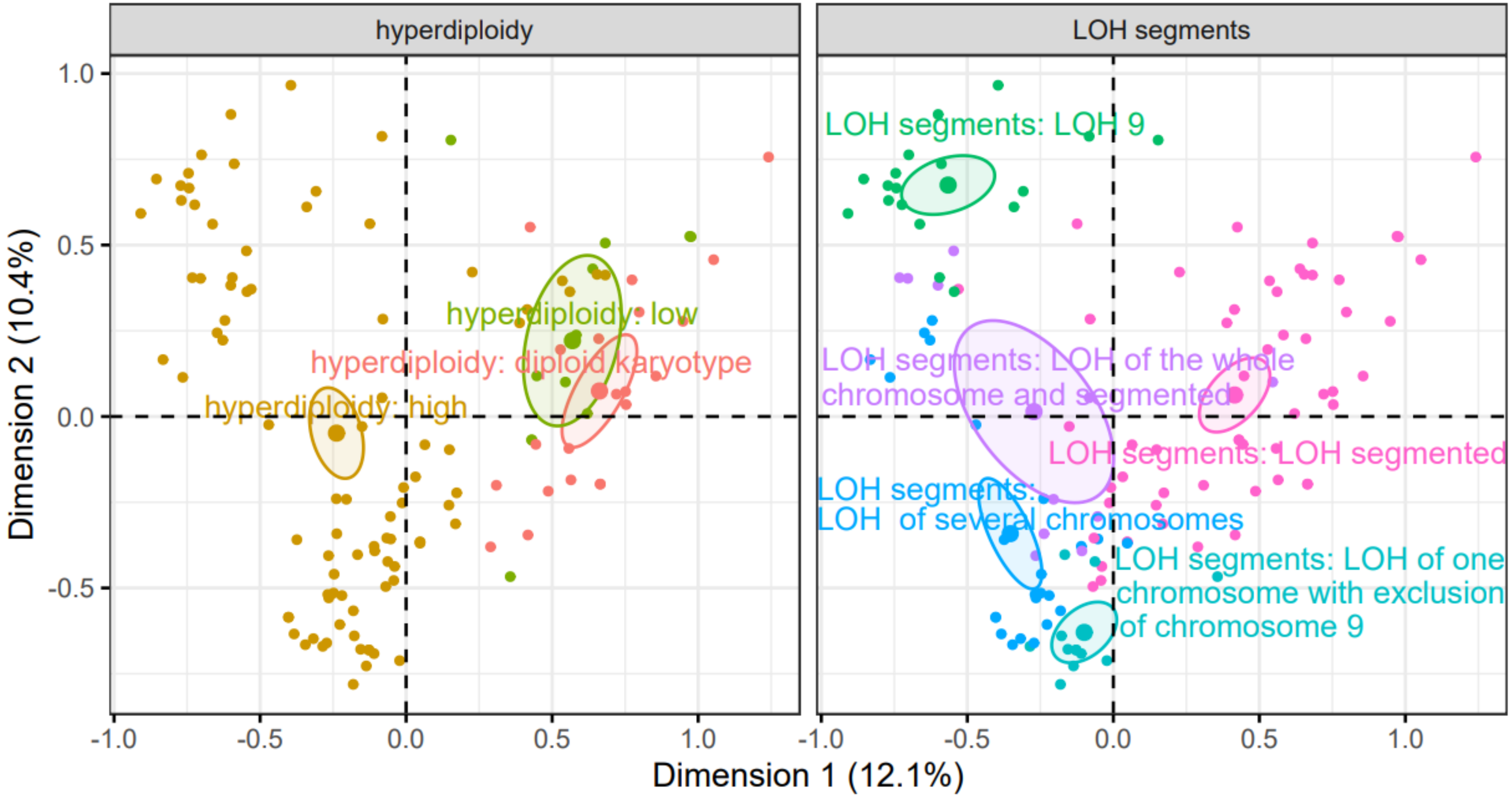

Supplementary Figure S4. The distribution of the patients on two-dimensional plot grouped by individual parameters in patients with B-ALL (hyperdiploidy, LOH segments) colored by the categories.

The figure displays the distribution of patients with B-ALL across the first two dimensions derived from Multiple Correspondence Analysis (MCA). Each dot represents a patient, and individuals are color-coded and grouped based on two genomic features: hyperdiploidy status (left panel) and LOH segment patterns (right panel). The percentages on each axis reflect the proportion of total variance explained by Dimension 1 (12.1%) and Dimension 2 (10.4%).

Left panel – hyperdiploidy: high hyperdiploidy (orange) clusters on the lower left, low hyperdiploidy (green) appears mostly in the upper right quadrant, diploid karyotype (red) groups centrally but trends toward the right side of Dimension 1.

Right panel – LOH segments: LOH 9 (green) localizes toward the upper right. Segmental LOH (pink) is widely distributed across the right half of the map. LOH of the whole chromosome and segmented (purple) and LOH of several chromosomes (blue) appear in the lower left. LOH of one chromosome with exclusion of chromosome 9 (turquoise) clusters distinctly below the horizontal axis. These plots demonstrate that Dimension 1 is primarily driven by genomic alterations such as hyperdiploidy and LOH profiles, while Dimension 2 contributes additional separation, particularly among specific LOH segment types. The ellipses indicate clustering trends within each genetic subgroup.

Abbreviations: B-ALL – B-cell acute lymphoblastic leukemia; LOH – loss of heterozygosity.

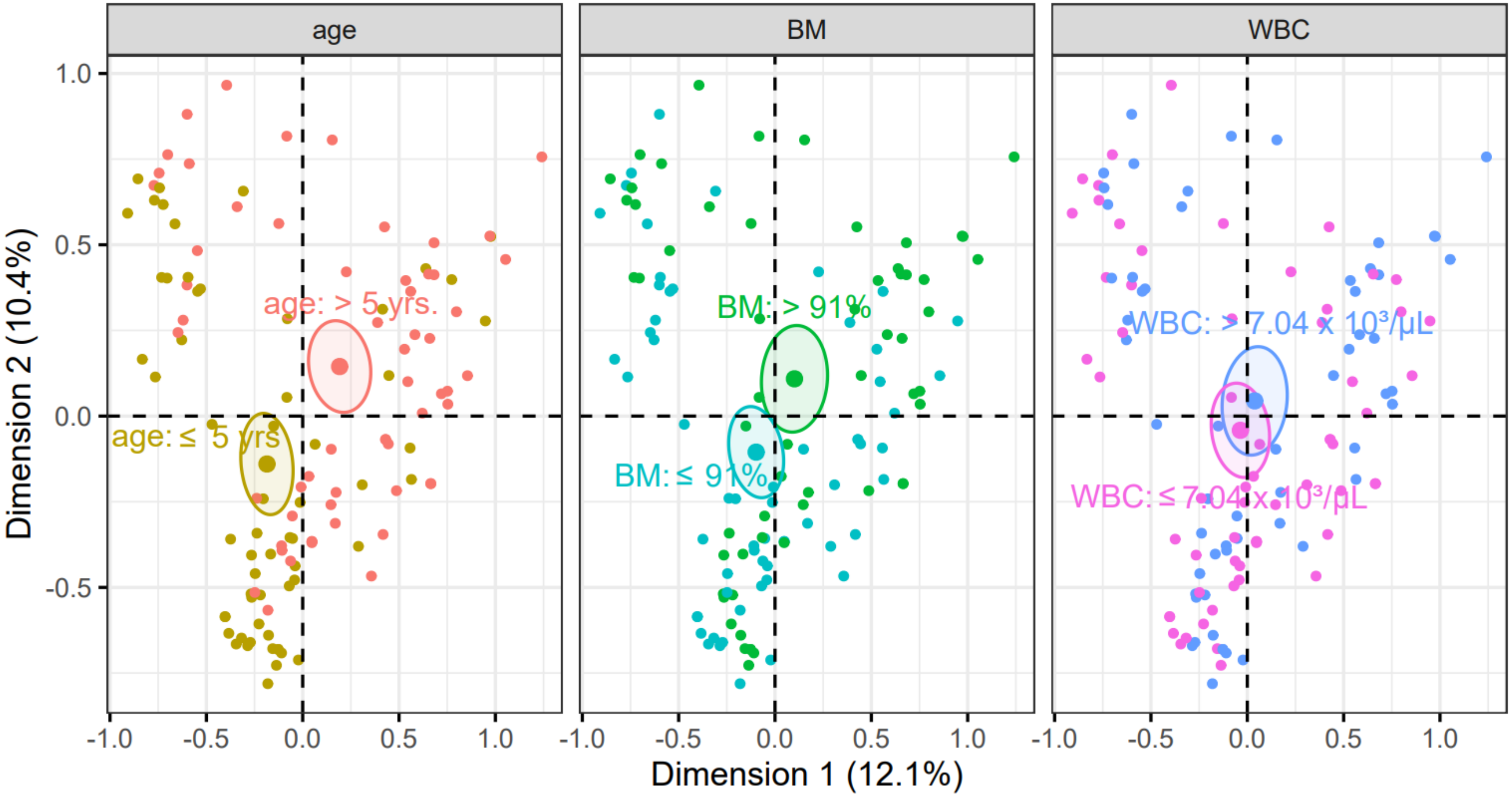

Supplementary Figure S5. The distribution of the patients on two-dimensional plot grouped by individual parameters in patients with B-ALL (age, BM, WBC) colored by the categories.

This figure presents factor maps showing the distribution of patients with T-ALL based on three clinical parameters: age, BM blast percentage at diagnosis, and WBC count at diagnosis. Each dot represents a patient, plotted across the first two MCA dimensions (Dimension 1: 12.1% of explained variance; Dimension 2: 10.4%).
Left panel (Age): patients aged ≤5 years (yellow) cluster toward the left side of Dimension 1, while those aged >5 years (red) are distributed more centrally and rightward.
Middle panel (BM): patients with BM blast percentage ≤91% (cyan) cluster to the lower left of the map, while those with >91% (green) tend to group near the origin, slightly right-shifted.
Right panel (WBC): patients with WBC count ≤7.04×10^3/μL (pink) are spread mostly across the left and central areas, while those with >7.04×10^3/μL (purple) tend to group around the center-right of Dimension 1.
The ellipses illustrate the spatial grouping of patients sharing the same parameter category. These visualizations help highlight potential associations between clinical variables and genetic dimensions in T-ALL patient stratification.
Abbreviations: B-ALL – B-cell acute lymphoblastic leukemia; WBC - white blood cell count; BM - bone marrow; yrs. - years.

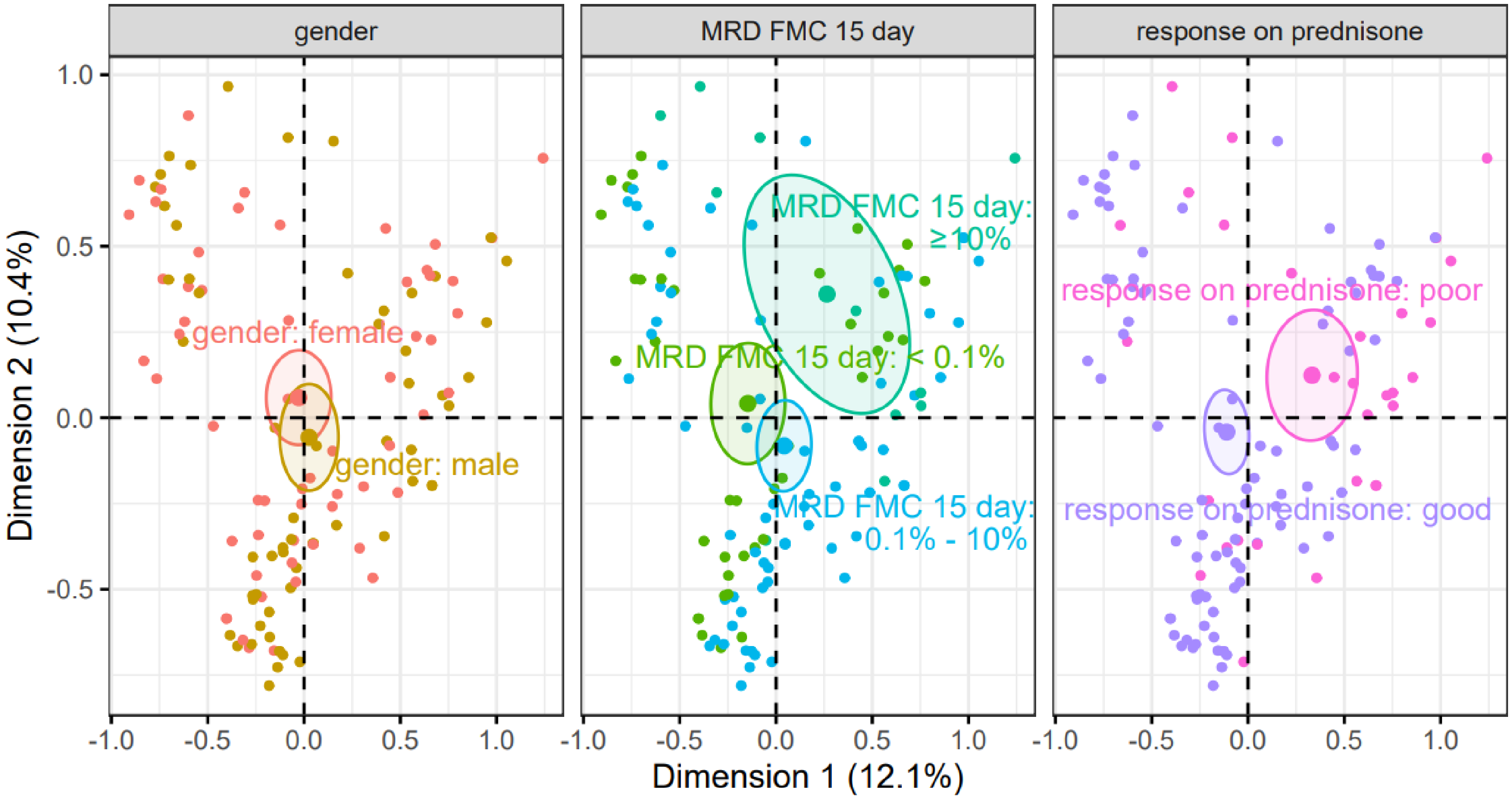

Supplementary Figure S6. The distribution of the patients on two-dimensional plot grouped by individual parameters in patients with B-ALL (gender, MRD FMC 15-day, response of prednisone) colored by the categories.

This figure presents three factor maps showing the distribution of T-ALL patients according to the following clinical parameters: gender, minimal residual disease (MRD) measured by flow cytometry on day 15, and response to prednisone. Each point represents an individual patient, positioned according to their profile along Dimension 1 (12.1% of variance) and Dimension 2 (10.4% of variance).
Left panel (Gender): patients are categorized by sex—female (pink) and male (orange). The distribution shows mild clustering around the center, with slight leftward grouping for males and rightward for females.
Middle panel (MRD FMC day 15): three groups are distinguished: MRD ≥10% (dark green), MRD between 0.1% and 10% (cyan), MRD <0.1% (light green). Higher MRD values are associated with positive values along Dimension 1, while lower MRD levels cluster more centrally or to the left, indicating their influence on patient stratification.
Right panel (Response to prednisone): patients with poor response (pink) show a wider spread, particularly toward the right half of the map. Good responders (purple) tend to group closer to the left side of Dimension 1, indicating a distinct pattern in early treatment sensitivity.
The ellipses indicate clustering tendencies within each subgroup, helping visualize how categorical clinical parameters relate to underlying molecular-genetic structure in T-ALL.
Abbreviations: T-ALL – T-cell acute lymphoblastic leukemia; FMC – flow cytometry; PCR – polymerase chain reaction.

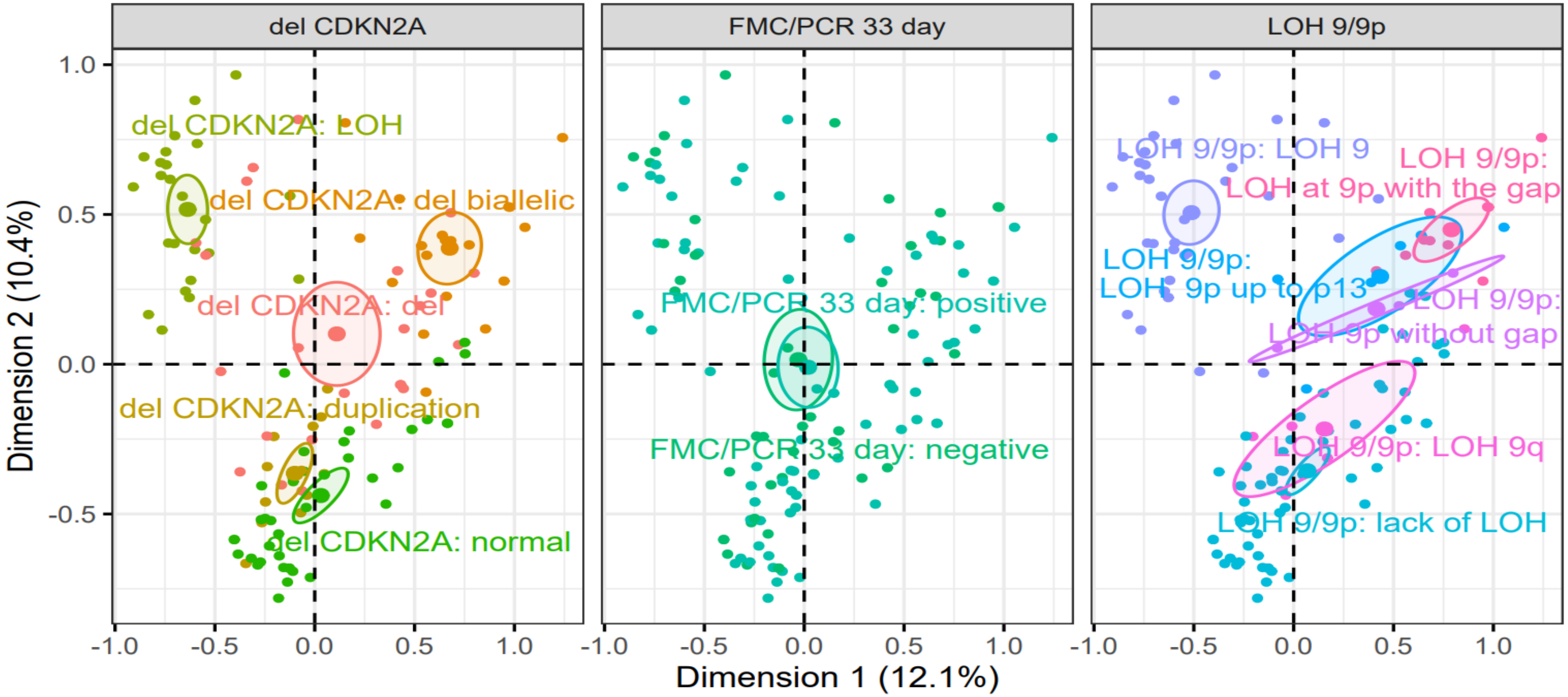

Supplementary Figure S7. The distribution of the patients on two-dimensional plot grouped by individual parameters in patients with B-ALL (del *CDKN2A*, MRD/PCR 33day, LOH 9/9p) colored by the categories.

This figure presents three factor maps derived from Multiple Correspondence Analysis (MCA), illustrating how patients with T-ALL are distributed across two dimensions (Dimension 1: 12.1%, Dimension 2: 10.4%) based on key genetic and molecular response variables: *CDKN2A* deletion status, MRD at day 33 (PCR/flow cytometry), and patterns of LOH on chromosome 9p.
Left panel (del *CDKN2A*): patients are grouped based on the status of the *CDKN2A* gene. Normal *CDKN2A* (dark green) clusters to the lower left. *CDKN2A* duplication (light green) is located below the x-axis, mid-left. Monoallelic deletion (del) (red) and biallelic deletion (orange) are centered but shift slightly rightward on Dimension 1. LOH with *CDKN2A* loss (olive) appears near the top-left quadrant. This pattern suggests strong dimension-based separation according to *CDKN2A* genetic alterations.
Middle panel (MRD/PCR at day 33): patients are divided by minimal residual disease status. Negative MRD (green) clusters on the left. Positive MRD (cyan) groups centrally and rightward on Dimension 1. This demonstrates the impact of molecular treatment response on patient stratification.
Right panel (LOH on 9p): this map shows distinct LOH patterns affecting chromosome 9p, categorized as: LOH at 9p with a gap (magenta) – upper right quadrant; LOH 9p up to p13 (purple) – mid-right; LOH 9q (pink) – lower right; LOH at 9 with no gap (blue) – top center; Lack of LOH on 9p (light blue) – clusters to the far left.
These distributions suggest that specific LOH patterns on chromosome 9p are highly informative for molecular subgrouping.
Abbreviations: MCA – Multiple Correspondence Analysis; T-ALL – T-cell acute lymphoblastic leukemia; del *CDKN2A* – deletion of the *CDKN2A* gene; LOH – loss of heterozygosity; MRD – minimal residual disease; FMC – flow cytometry; PCR – polymerase chain reaction.

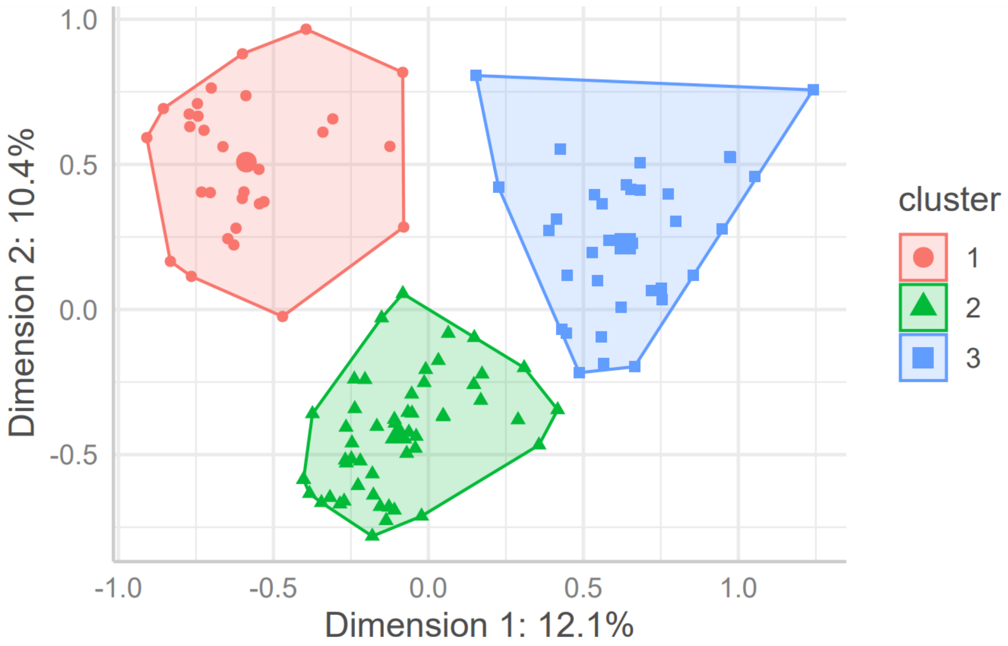

Supplementary Figure S8. Visualization of the positions of the identified clusters in a two-dimensional space, along with the results of individual patients with B-ALL represented by distinct shapes, where the larger shape denotes the center of each cluster.

Note: Clusters 1 and 3 are similarly positioned along the Y-axis but differ significantly on the X-axis. Cluster 1 is located in the negative X space, while Cluster 3 exclusively occupies positive X values. Regarding the Y-axis, both clusters extend into negative and positive spaces but are mostly in the positive region. In contrast, Cluster 2 is positioned between Clusters 1 and 3 on the X-axis, with distinctly lower values along the Y-axis.

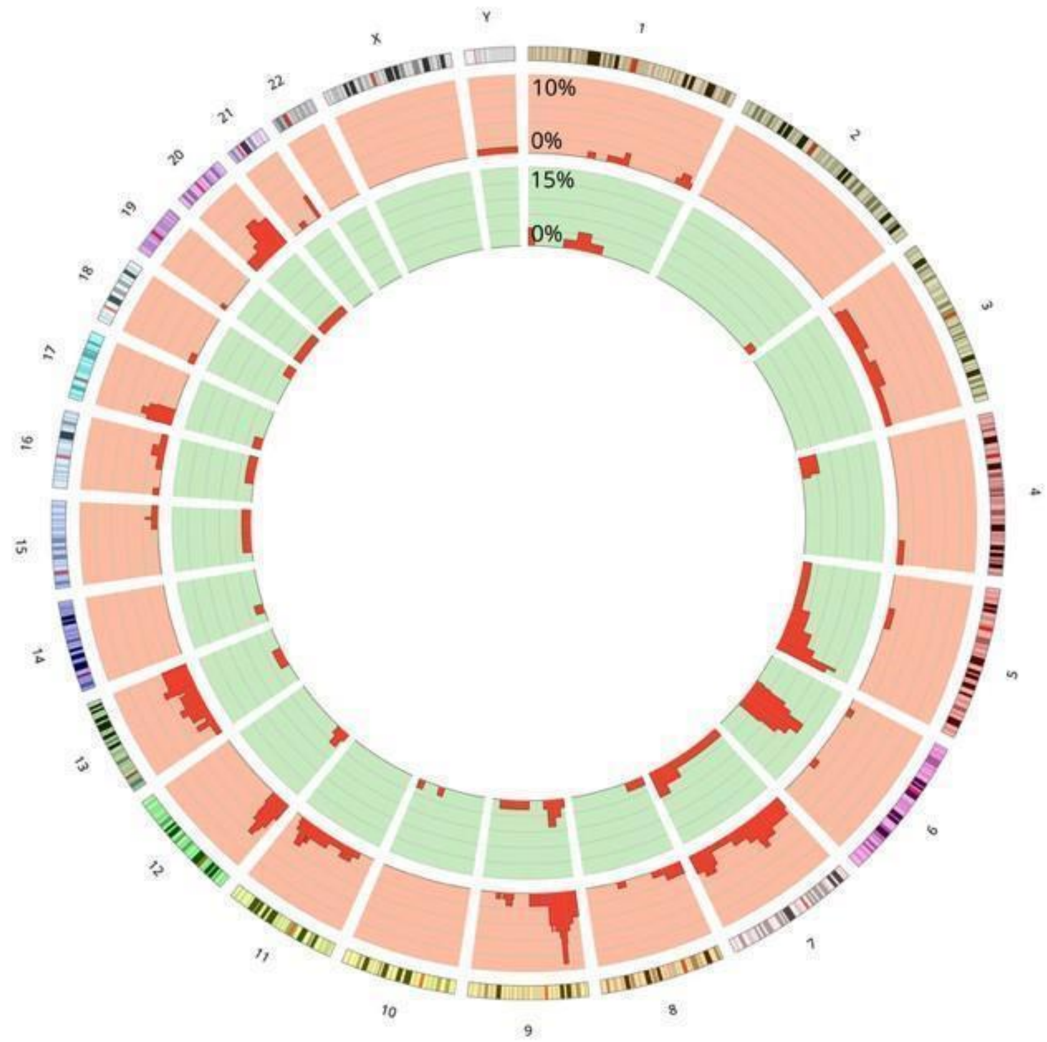

Supplementary Figure S9. Deletions in T-ALL (inner circle) and B-ALL (outer circle) divided into chromosomes.

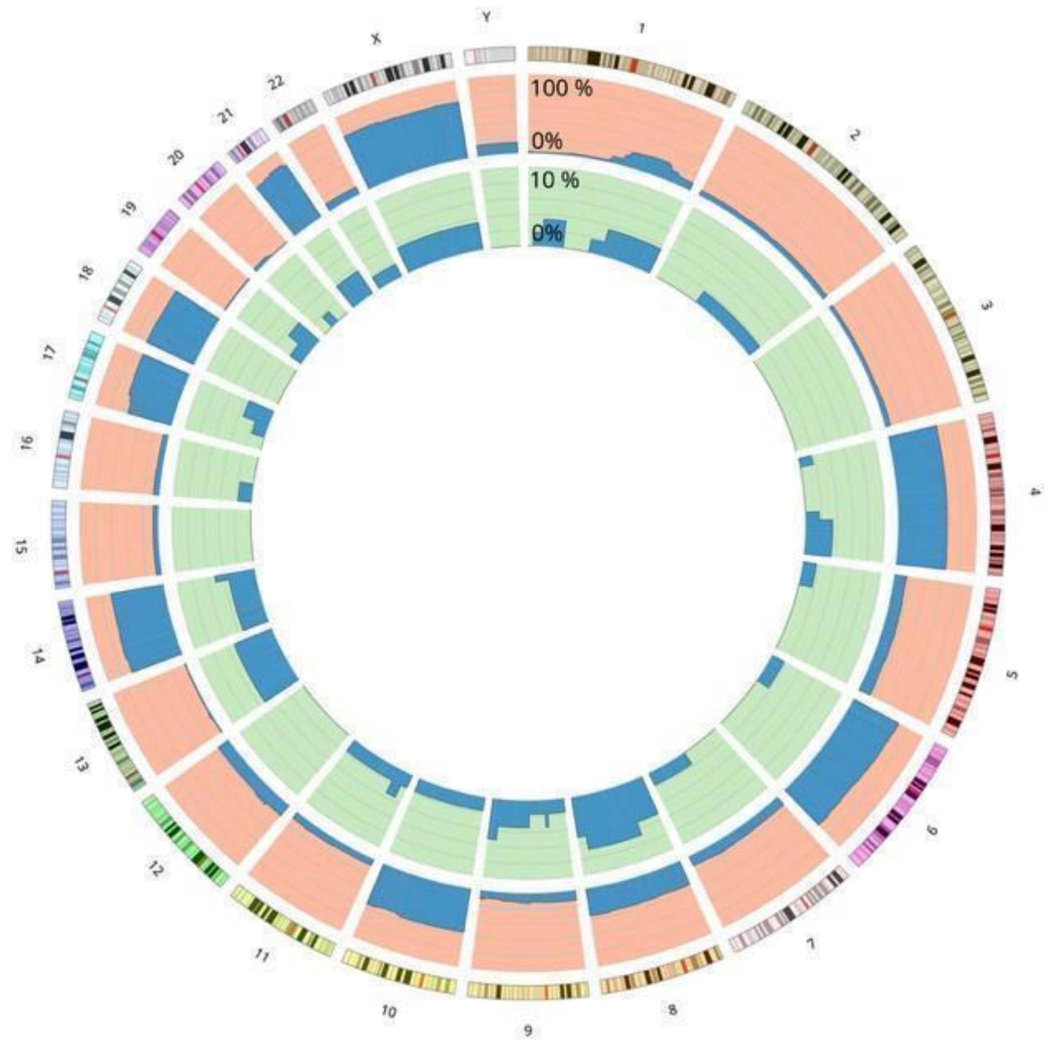

Supplement Figure S10. Duplications in T-ALL (inner circle) and B-ALL (outer circle) divided into chromosomes.

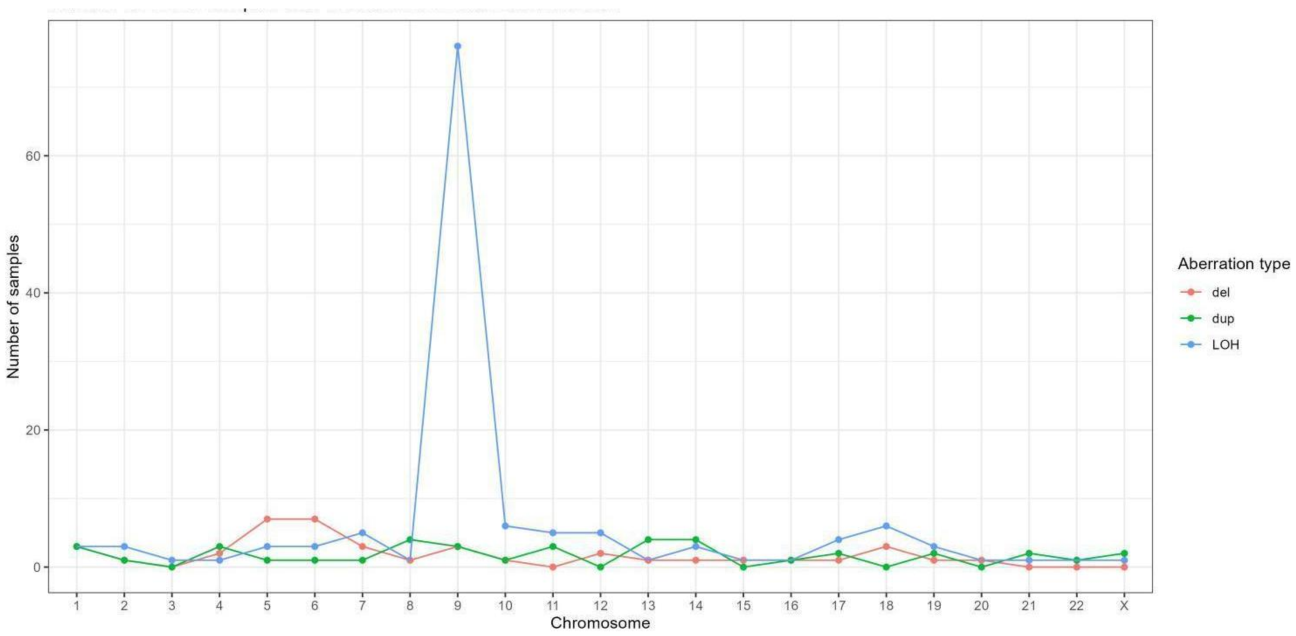

Supplement Figure S11. Number of T-ALL samples with aberration in each chromosome.

This line plot illustrates the number of samples presenting specific types of chromosomal aberrations—deletions (del), duplications (dup), and loss of heterozygosity (LOH)—across chromosomes 1 to 22 and the X chromosome.

LOH events (blue line) are most pronounced on chromosome 9, where a sharp peak represents over 65 samples, highlighting this region as the most frequently affected by LOH in the T-ALL cohort. Deletions (red line) and duplications (green line) occur at relatively low and consistent frequencies across all chromosomes, without any dominant peak, although slight elevations are noted on chromosomes 7, 12, and 21.

These findings reinforce the central role of chromosome 9 LOH, particularly on the short arm (9p), in the genetic landscape of T-ALL, while other chromosomal changes appear more sporadic and less frequent.

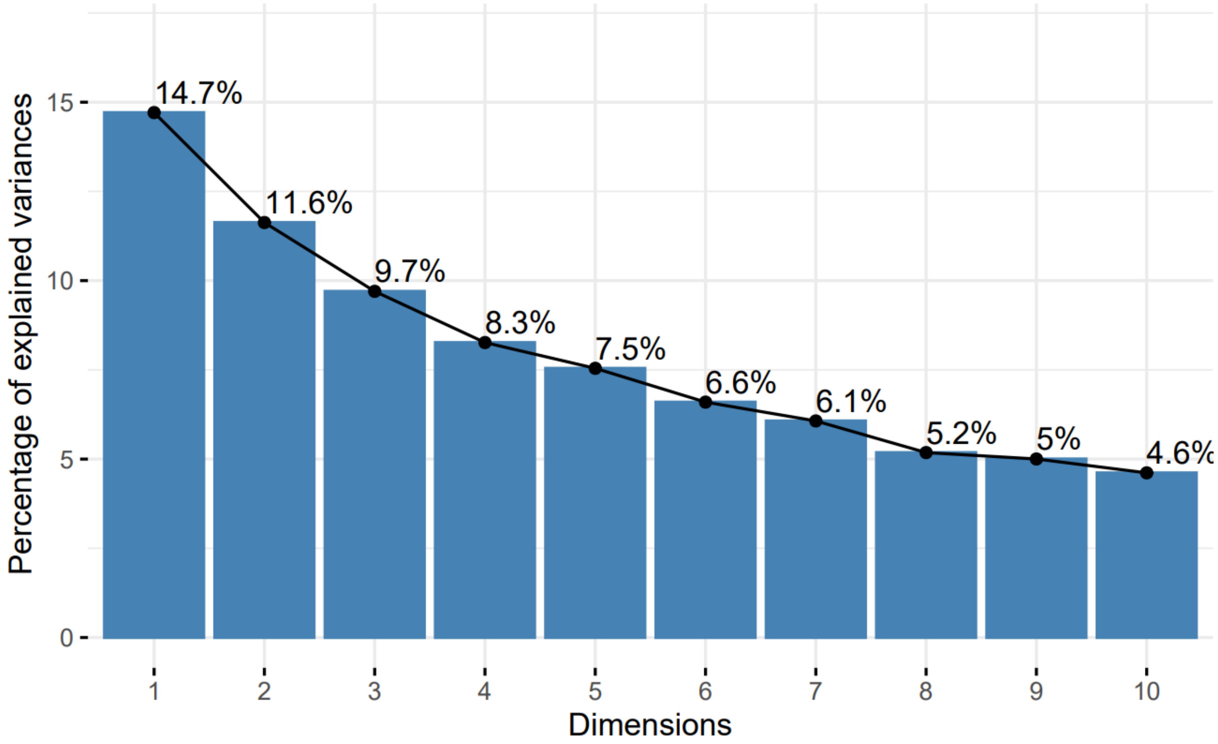

Supplementary Figure S12. The variance of the 11 analyzed variables, through the application of Multiple Correspondence Analysis (MCA), was fully explained within an 18-dimensional framework. Scree plot showing explained variance percentages for the first 10 dimensions in patients with T-ALL.

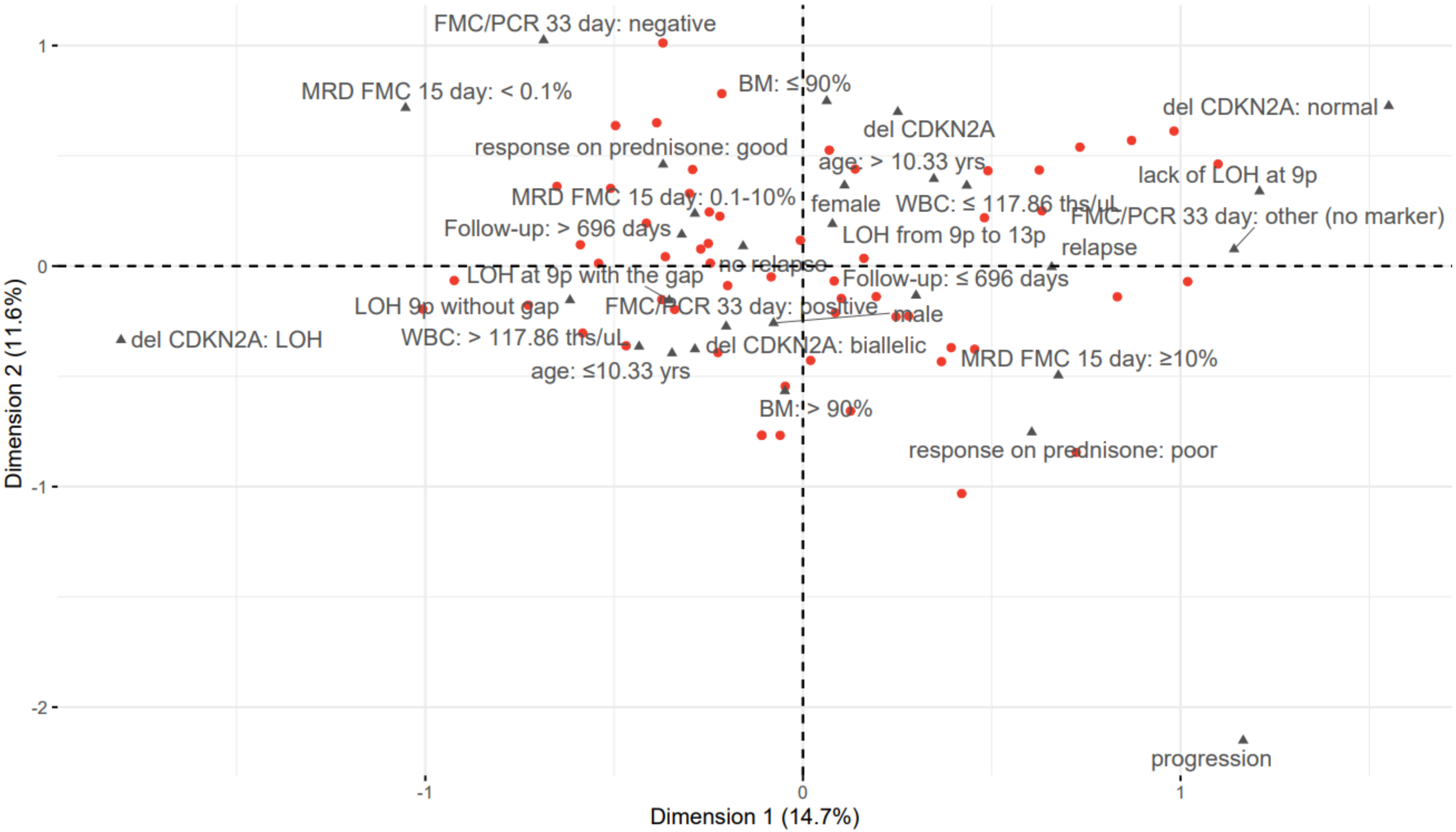

Supplementary Figure S13. Global data patterns in two-dimensional plot in patients with T-ALL (biplot): patient results (red points) and parameter categories (gray triangles).

Supplementary Figure S14-S15. These figures provide a visual representation of how patients are grouped based on their characteristics, allowing for a comprehensive understanding of the relationships between the parameters and the dimensions identified through the analysis with drawing confidence ellipses around categories.

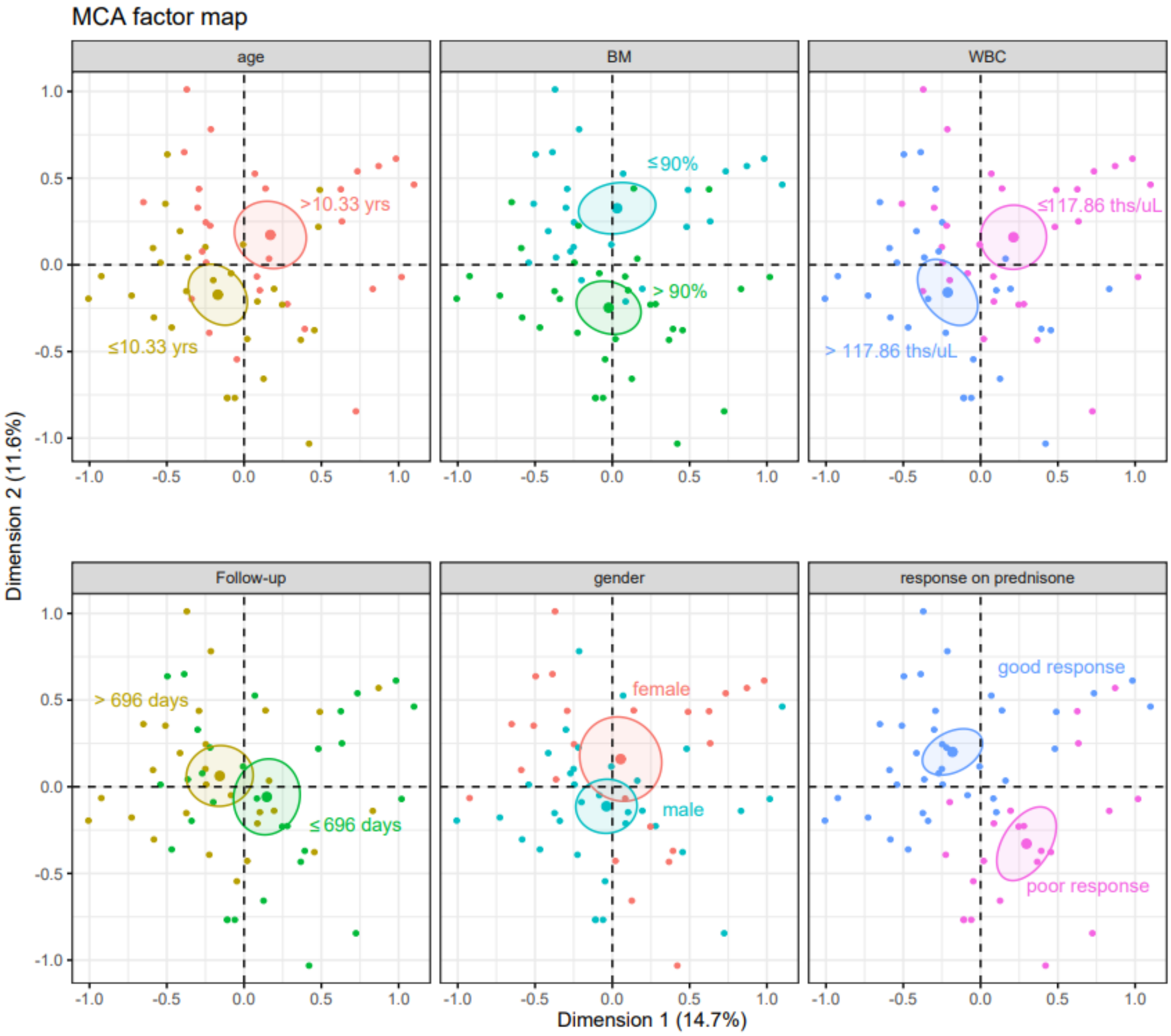

Supplementary Figure S14. The distribution of the patients on two-dimensional plot grouped by individual parameters in patients with T-ALL (age, BM, WBC, Follow-up, gender, response of prednisone) colored by the categories.

This figure presents factor maps for T-ALL patients, displaying the relationships between clinical parameters and the first two MCA dimensions (Dimension 1: 14.7%, Dimension 2: 11.6% of explained variance). Each subplot corresponds to a different clinical variable, with individual patients represented as dots and color-coded according to categorical groupings.

Top row:

Age: patients aged ≤10.33 years (yellow) tend to cluster to the left, while older patients >10.33 years (red) shift toward the right along Dimension 1.

BM blast percentage (BM): patients with BM ≤90% (cyan) and >90% (green) form overlapping but distinguishable groups along Dimension 2.

WBC count: patients with WBC ≤117.86×10³/μL (pink) are spread toward the center, while higher WBC counts >117.86×10³/μL (blue) slightly cluster toward the right side of Dimension 1.
Bottom row:
Follow-up duration: patients followed for ≤696 days (green) localize leftward, while those with longer follow-up ≥696 days (yellow) spread more widely, mostly above the x-axis.
Gender: male patients (blue) and female patients (red) are evenly distributed, though females slightly group rightward along Dimension 1.
Response to prednisone: good responders (blue) localize to the left of Dimension 1, while poor responders (pink) appear on the right, indicating clear separation along this axis.
The ellipses in each panel represent confidence regions that highlight the clustering tendencies of patients within each category. Together, the maps demonstrate how clinical variables contribute to patient heterogeneity and dimensional structure in T-ALL.
Abbreviations: MCA – Multiple Correspondence Analysis; T-ALL – T-cell acute lymphoblastic leukemia; BM – percentage of blasts in bone marrow at diagnosis; WBC - white blood cell count; ths/μL – thousands per microliter.

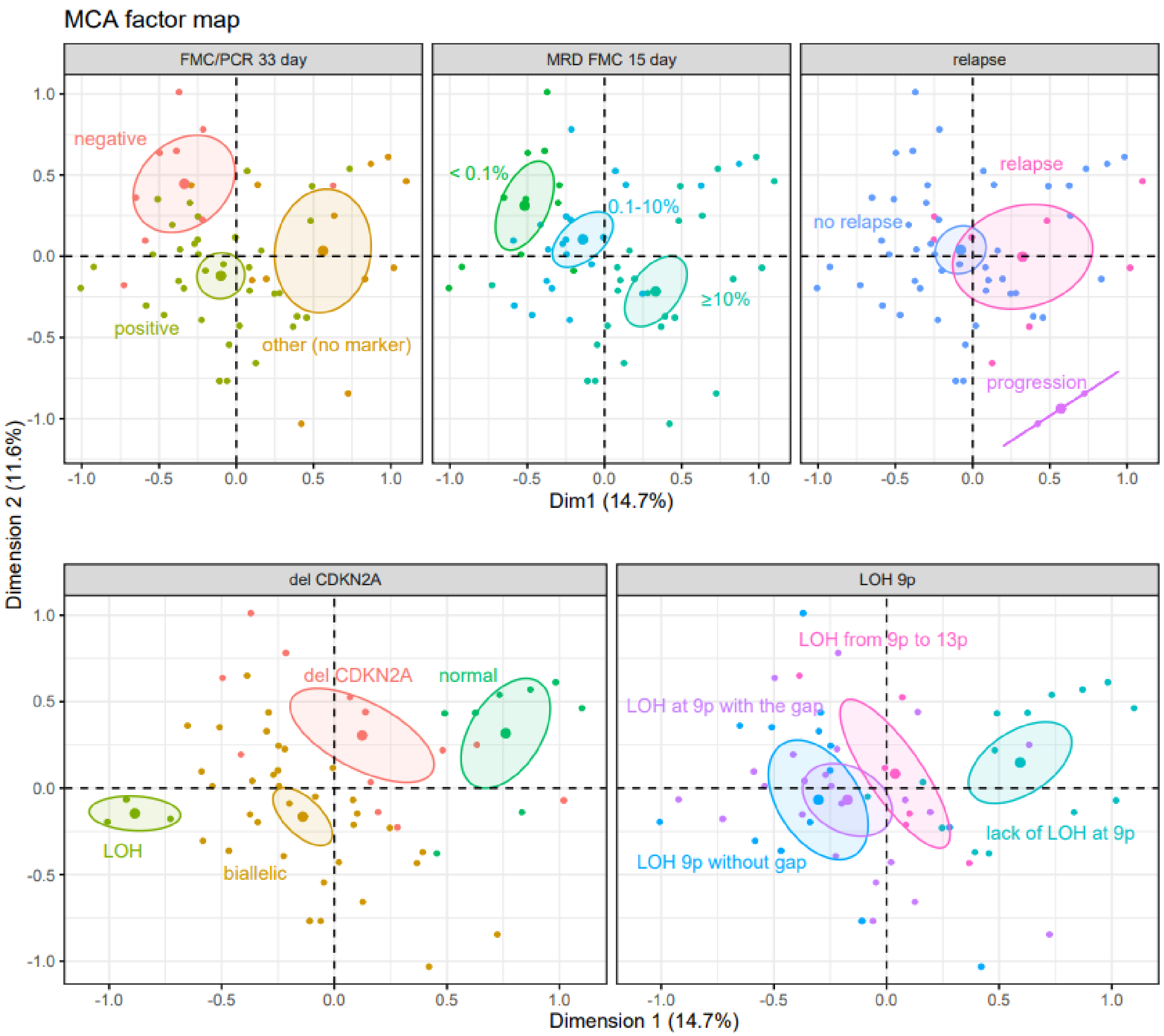

Supplementary Figure S15. The distribution of the patients on two-dimensional plot grouped by individual parameters in patients with T-ALL (MRD /PCR 33day, MRD FMC 15th day, relapse, del *CDKN2A*, LOH 9p) colored by the categories.

This figure presents factor maps displaying the distribution of T-ALL patients across the first two dimensions (Dimension 1: 14.7%, Dimension 2: 11.6%). Each subplot represents a different molecular or genetic parameter, with patient data points color-coded by subgroup and ellipses illustrating grouping trends.

Top row:

FMC/PCR at Day 33: patients are divided by PCR-based MRD results: negative (red) patients cluster toward the upper left, positive (green) are centered, other (no marker) (yellow) localize slightly rightward.

MRD by Flow Cytometry at Day 15: stratified into: <0.1% (dark green) – lower left cluster, 0.1–10% (light blue) – central group, ≥10% (cyan) – positioned further right, indicating poor early response.

Relapse Status: patients with: no relapse (blue) group around the origin, relapse (pink) shift toward the right side, progression (violet) is more sparsely distributed but trends even further right along Dimension 1.
Bottom row: *CDKN2A* Deletion Status: subgroups include: normal *CDKN2A* (green) – central-right cluster, monoallelic deletion (del *CDKN2A*) (red) – mid-right, biallelic deletion (orange) and LOH with deletion (yellow) – clustered leftward.
LOH at Chromosome 9p: divided into: LOH from 9p to 13p (pink) and LOH at 9p with the gap (violet) – upper and mid-right clusters, LOH 9p without gap (blue) – central cluster, Lack of LOH at 9p (aqua) – distinct group slightly toward Dimension 1 negative values.
These maps illustrate the spatial and dimensional separation of patient subgroups based on response and genetic features, showing that *CDKN2A* deletion, LOH patterns on chromosome 9p, and MRD significantly contribute to molecular stratification within T-ALL.
Abbreviations: MCA – Multiple Correspondence Analysis; T-ALL – T-cell acute lymphoblastic leukemia; MRD – minimal residual disease; FMC – flow cytometry; PCR – polymerase chain reaction; LOH – loss of heterozygosity.

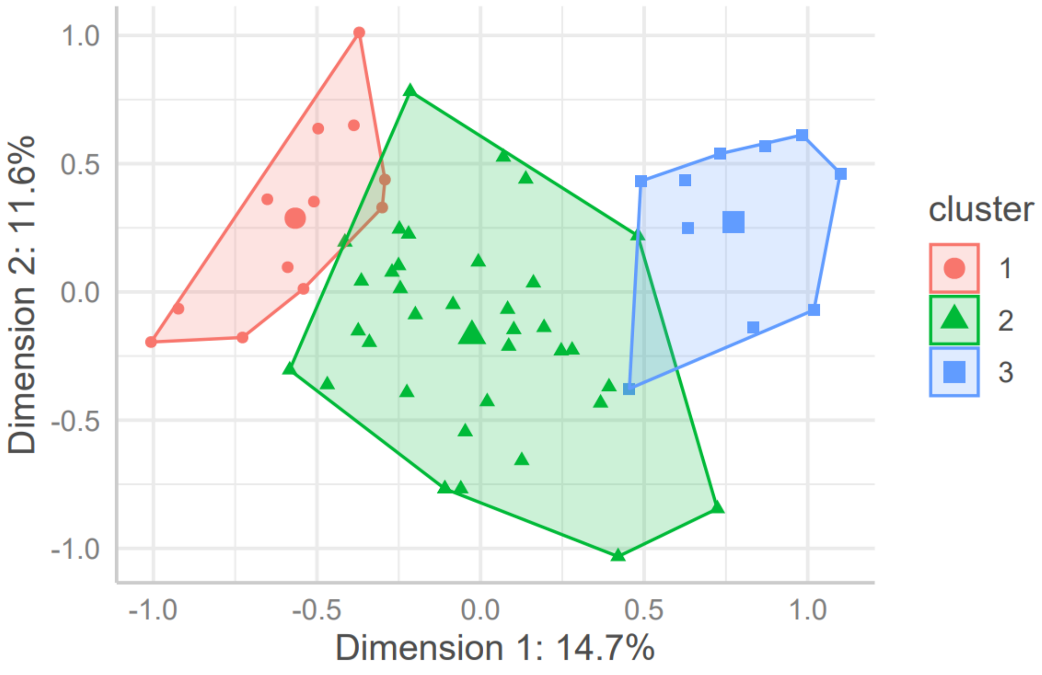

Supplementary Figure S16. Visualization of the positions of the identified clusters in a two-dimensional space, along with the results of individual patients with T-ALL represented by distinct shapes, where the larger figure denotes the center of each cluster.

Note: The clusters primarily discriminated patients according to the X-axis. Cluster 1 exhibited exclusively negative values along the X-axis, while predominantly positive values were observed along the Y-axis. This cluster was symmetrically positioned around the origin, with a spread of approximately 0.6 to 0.7 units on either side of zero. The center of Cluster 1 was located slightly below zero on the Y-axis. In contrast, Cluster 3 was characterized by the highest values along the X-axis, with a positive center along the Y-axis.

Supplementary S2.3 Description

Supplementary S2.3.1 Statistical description of results in B-ALL group

The results from the chi-square tests in Supplementary Table 5 indicate significant associations between key genetic and clinical variables and the clusters identified in HCPC analysis. The p-values are exceptionally low for most variables, suggesting that these factors play a critical role in characterizing distinct subgroups of pediatric B-cell leukemia patients across clusters. Based on the results, the most common recurrent genetic changes in B-ALL-LOH+ were observed, such as: *CDKN2A* deletion, loss of heterozygosity of various sizes within the short arm of chromosome 9 and the entire chromosome 9, as well as segmental and entire chromosome losses of heterozygosity appearing differently in patients with high hyperdiploidy (HEH), low hyperdiploidy (LH) and normal diploidy (ND). These data were correlated with the clinical data listed in Supplementary Table 6

Supplementary S2.3.2 Statistical description of results in T-ALL group

The p-values reveal a high level of statistical significance for several parameters. Specifically, deletion *CDKN2A* (del *CDKN2A*), MRD FMC 15 day, LOH 9p, and MRD PCR 33 day all exhibit $p < 0.001$, indicating a very strong association with the clusters. In contrast, the parameters "Response of the prednisone," "Gender," and "WBC" show moderate significance, with $p = 0.007$, 0.025, and 0.037, respectively. While these values are higher than those of the previously mentioned parameters, they still indicate statistically significant associations, suggesting that these variables also contribute to the differentiation of clusters, albeit to a lesser extent (Supplementary Table 8).The data presented in Supplementary Table 9 provides a detailed examination of clusters based on individual categories, highlighting significant results through the use of v-tests and p-values. Each category is analyzed in terms of its distribution across the clusters.
